# Anthelmintic Potential of Agelasine Alkaloids from the Australian Marine Sponge *Agelas axifera*

**DOI:** 10.3390/md23070276

**Published:** 2025-07-01

**Authors:** Kanchana Wijesekera, Aya C. Taki, Joseph J. Byrne, Darren C. Holland, Ian D. Jenkins, Merrick G. Ekins, Anthony R. Carroll, Robin B. Gasser, Rohan A. Davis

**Affiliations:** 1 Institute for Biomedicine and Glycomics, Griffith University, Brisbane, QLD 4111, Australia; kanchana.wijesekera@griffithuni.edu.au (K.W.); i.jenkins@griffith.edu.au (I.D.J.); a.carroll@griffith.edu.au (A.R.C.); 2 School of Environment and Science, Griffith University, Brisbane, QLD 4111, Australia; 3 Department of Veterinary Biosciences, Melbourne Veterinary School, Faculty of Science, The University of Melbourne, Parkville, VIC 3010, Australia; aya.taki@unimelb.edu.au (A.C.T.); byrnej1@unimelb.edu.au (J.J.B.); 4 School of Molecular Sciences, The University of Western Australia, Crawley, WA 6009, Australia; darren.holland@uwa.edu.au; 5 School of Environment and Science, Griffith University, Gold Coast, QLD 4222, Australia; 6 Biodiversity and Geosciences, Queensland Museum, South Brisbane BC, QLD 4101, Australia; merrick.ekins@qm.qld.gov.au; 7 NatureBank, Griffith University, Brisbane, QLD 4111, Australia

**Keywords:** *Agelas axifera*, agelasine, anthelmintic activity, *Haemonchus contortus*, *Caenorhabditis elegans*, deuterium exchange

## Abstract

A recent high-throughput screening of the NatureBank marine extract library (7616 samples) identified an extract from the Australian marine sponge *Agelas axifera* with in vitro activity against an economically important parasitic nematode, *Haemonchus contortus* (barber’s pole worm). The bioassay-guided fractionation of the CH_2_Cl_2_/MeOH extract from *A. axifera* led to the purification of a new diterpene alkaloid, agelasine Z (**1**), together with two known compounds agelasine B (**2**) and oxoagelasine B (**3**). Brominated compounds (–)-mukanadin C (**4**) and 4-bromopyrrole-2-carboxylic acid (**5**) were also isolated from neighbouring UV-active fractions. All compounds, together with agelasine D (**6**) from NatureBank’s pure compound library, were tested for in vitro anthelmintic activity against exsheathed third-stage (xL3s) and fourth-stage larvae (L4s) of *H. contortus* and young adult *Caenorhabditis elegans*. Compounds **1**, **2** and **6** induced an abnormal “skinny” phenotype, while compounds **2** and **6** also reduced the motility of *H. contortus* L4s by 50.5% and 51.8% at 100 µM, respectively. The minimal activity of agelasines against *C. elegans* young adults suggests a possible species-specific mechanism warranting further investigation. For the first time, the unexpected lability of agelasine H-8′ was explored using kinetic studies, revealing rapid deuterium exchange in MeOH-*d*_4_ at room temperature.

## 1. Introduction

Gastrointestinal nematodes pose a significant global health burden by causing severe diseases in livestock, significantly affecting productivity, and are considered one of the most important production-limiting diseases of grazing animals worldwide [1]. Among the nematodes of the order Strongylida, *Haemonchus contortus*, commonly known as barber’s pole worm, stands out as one of the most pathogenic parasites of small ruminants [2]. The control of these parasites has primarily relied on anthelmintic drugs, which have been crucial in combatting nematode infections. However, the effectiveness of these drugs is increasingly threatened by the emergence of anthelmintic resistance. Notably, *H. contortus* was the first species to develop resistance to commercial anthelmintic drugs [3]; more concerning is the emergence of resistance to monepantel, a next-generation drug typically reserved for resistant infections, emphasizing the need for new treatment options [4]. In light of this challenge, recent investigations have identified *H. contortus* as a valuable model for the identification and evaluation of anthelmintic natural products [5,6,7]. This approach not only addresses the immediate concern of drug resistance in *H. contortus* but also paves the way for discovering novel treatments against other parasitic nematodes.

As part of a drug discovery effort aimed at identifying new anthelmintic leads from natural resources, we recently screened the NatureBank [8] marine extract library collection (n = 7616) against larvae of *H. contortus* in a high-throughput screening (HTS) bioassay [9]. This effort revealed 58 hit extracts [10,11] one of which was derived from the Australian marine sponge *Agelas axifera*. This extract inhibited larval motility by 96% (90 h) and larval development by >80% (168 h) and induced a curved (*Cur*) phenotype in 80% of larvae [9].

Marine sponges from the genus *Agelas* (class Demospongiae, family Agelasidae) are widely distributed and are known for their structurally diverse diterpene alkaloids and bromopyrroles [12,13]. In the present study, we report the bioassay-guided fractionation and purification of an *A. axifera* extract with inhibitory activity against *H. contortus* larvae, yielding a new diterpene alkaloid, which we named agelasine Z (**1**), as well as two known compounds agelasine B (**2**) [14] and oxoagelasine B (**3**) [15]. Moreover, two other known pyrrole alkaloids (–)-mukanadin C (**4**) [16] and 4-bromopyrrole-2-carboxylic acid (**5**) [17] were also isolated. All of the isolated compounds (**1**–**5**), along with agelasine D (**6**) [14] from the NatureBank pure compound library, were individually tested in vitro for nematocidal or nematostatic activity against *H. contortus* and *Caenorhabditis elegans*.

## 2. Results and Discussion

### 2.1. Bioassay-Guided Fractionation of Compounds from A. axifera

**Figure 1:**
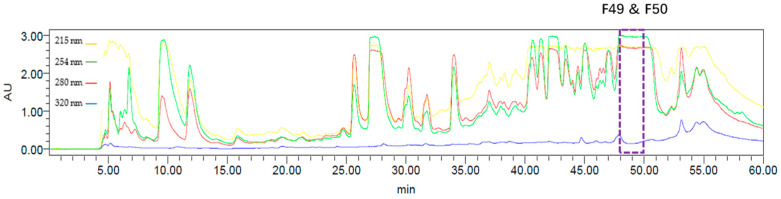
RP-HPLC (MeOH/H_2_O/0.1% TFA) chromatogram of CH_2_Cl_2_/MeOH extract of *Agelas axifera*; F49 and F50 displayed anthelmintic activity.

A freeze-dried and ground sample of *A. axifera* was extracted exhaustively with *n*-hexane, CH_2_Cl_2_ and MeOH; the CH_2_Cl_2_ and MeOH extracts were combined, and a portion of this material was subjected to reversed-phase HPLC (RP-HPLC, MeOH/H_2_O/0.1% TFA) (Figure 1) to produce 60 fractions that were then evaluated for anthelmintic activity using an established xL3 motility assay [11]. Fractions 49 (F49) and 50 (F50) showed moderate motility reduction (37.9% and 26.3%, respectively) at 168 h despite the strong activity observed for the original sponge extract. However, these fractions were able to induce an evisceration (*Evi*) phenotype in 19.6% and 12.5% of larvae, respectively. The *Evi* phenotype is lethal and displays an anterior protrusion in the affected larvae, as previously described [18], relative to the untreated control. Consequently, F49 and F50 fractions were selected for further purification and characterization studies.

**Figure 2:**
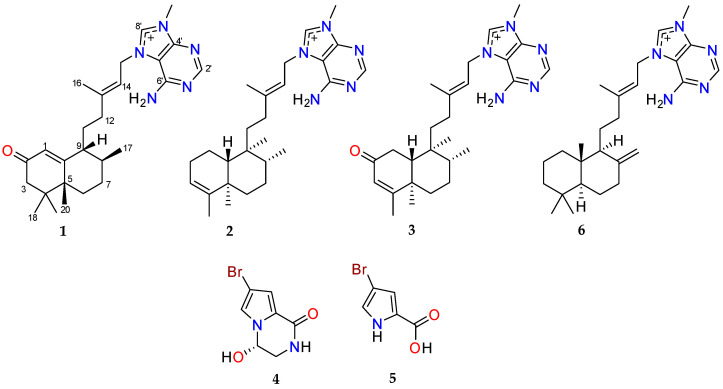
Chemical structures of agelasine Z (**1**), agelasine B (**2**), oxoagelasine B (**3**), (–)-mukanadin C (**4**), 4-bromopyrrole-2-carboxylic acid (**5**) and agelasine D (**6**). All alkaloids were isolated as their TFA salts.

An analysis of the ^1^H NMR data for F49 and F50 suggested that they contained a mixture of agelasine-type diterpenoid alkaloids [15]. Therefore, the two fractions were combined and subjected to purification by RP-HPLC using a different gradient system to yield one new compound agelasine Z (**1**) and two known alkaloids, agelasine B (**2**) and oxoagelasine B (**3**). An analysis of other UV-active HPLC fractions enabled the identification of the previously reported marine natural products (–)-mukanadin C (**4**) and 4-bromopyrrole-2-carboxylic acid (**5**). The known compounds were confirmed following a comparison of experimental NMR, MS and specific rotation data with their respective literature values [14,16,17,19]. Agelasine D (**6**) was obtained from the NatureBank pure compound library (Figure 2) following a substructure search of the NatureBank database [8].

The HRESIMS of compound **1** revealed an ion at *m*/*z* 436.3060 [M]^+^ (calcd for C_26_H_38_N_5_O, 436.3071) that allowed a molecular formula of C_26_H_38_N_5_O^+^ to be assigned. A comparison of the ^1^H and ^13^C NMR data of **1** with the previously reported agelasine alkaloid nakamusine C showed a high degree of similarity but was not identical, suggesting that **1** was an isomer of nakamusine C [20]. The ^1^H NMR data for **1** in MeOH-*d*_4_ (Table 1 and Table S1) exhibited resonances for two protons at δ_H_ 9.33 (H-8′) and 8.47 (H-2′) that were characteristic for *N*^9^-adeninium alkaloids [15].

A further analysis of the ^1^H NMR data showed that **1** contained two olefinic protons (*δ*_H_ 5.87 and 5.55), an allylic methylene unit (*δ*_H_ 5.21), an *N*-methyl group (*δ*_H_ 3.98), two methines (*δ*_H_ 2.19 and 1.48), five methylene units (*δ*_H_ 2.42/1.98, 2.11/1.43, 2.10/1.78, 2.08/1.50, 1.64), an olefinic methyl group (*δ*_H_ 1.88) and four upfield methyl groups (*δ*_H_ 1.20, 1.04, 1.01, 0.98). The ^13^C NMR data (Table 1) contained 26 carbon resonances, including six sp^2^ non-protonated carbons (*δ*_C_ 202.7, 176.4, 154.1, 150.9, 149.1, 111.2), four sp^2^ methines (*δ*_C_ 157.1, 141.9, 122.6, 115.8), two sp^3^ quaternary carbons (*δ*_C_ 46.1, 35.4), two sp^3^ methines (*δ*_C_ 47.5, 47.2), six sp^3^ methylenes (*δ*_C_ 48.6, 47.8, 35.2, 31.6, 31.0, 31.0) and six methyl groups, one of which was an *N*-methyl (*δ*_C_ 32.0, 28.8, 27.8, 23.2, 17.0, 16.4).

**Figure 3:**
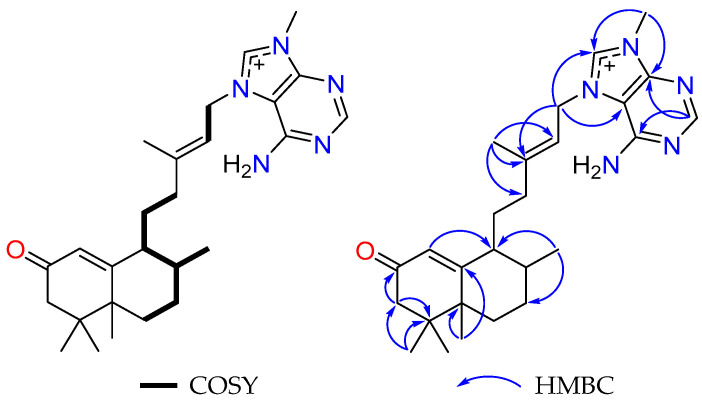
Key COSY and HMBC correlations of **1**.

The ^1^H-^1^H COSY data established two spin systems, which included -CH=CH_2_- and -CH_2_-CH_2_-CH(CH_3_)-CH-CH_2_-CH_2_-. Moreover, key HMBC correlations of H-1 to C9; H_2_-3 to C-2 and C-4; H_2_-15 to C-13, C-5’ and C-8’; H_3_-16 to C-12, C-13 and C-14; H_3_-17 to C-7 and C-9; H_3_-18 and H_3_-19 to C-3, C-4 and C-5; H_3_-20 to C-5, C-7 and C-10; H-2’ to C-4’ and C-6’; and H_3_-9’-Me to C-4’ and C-8’ (Figure 3) aided in the establishment of the planar structure of agelasine Z (**1**). Following a careful analysis of the 2D NMR data of **1**, it was identified that the C-20 methyl group of nakamusine C has been switched to C-5 in agelasine Z.

**Figure 4:**
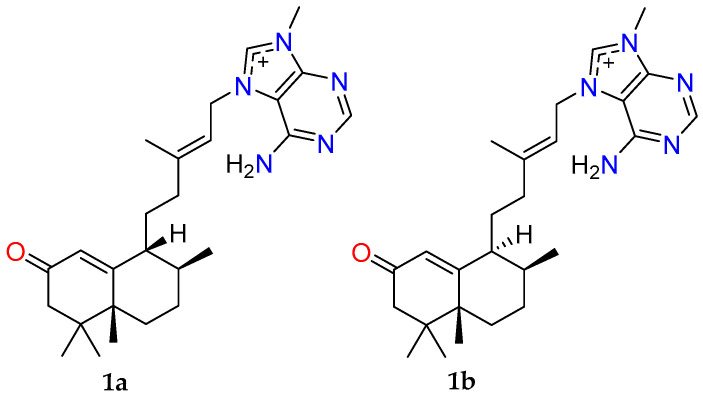
Two possible configurations for agelasine Z (**1**) following MeOH-*d*_4_ ROESY NMR analysis; (**1a**) 5R*, 8S*, 9R* and (**1b**) 5R*, 8S*, 9S*.

To determine the relative configuration of **1**, ROESY NMR data were acquired in MeOH-*d*_4_. However, poor signal dispersion led to the overlap of key proton resonances associated with the decalin core, thereby preventing the definitive assignment of the configuration at C-9. As a result, two potential diastereomers were proposed for agelasine Z (**1a** and **1b**, Figure 4).

**Figure 5:**
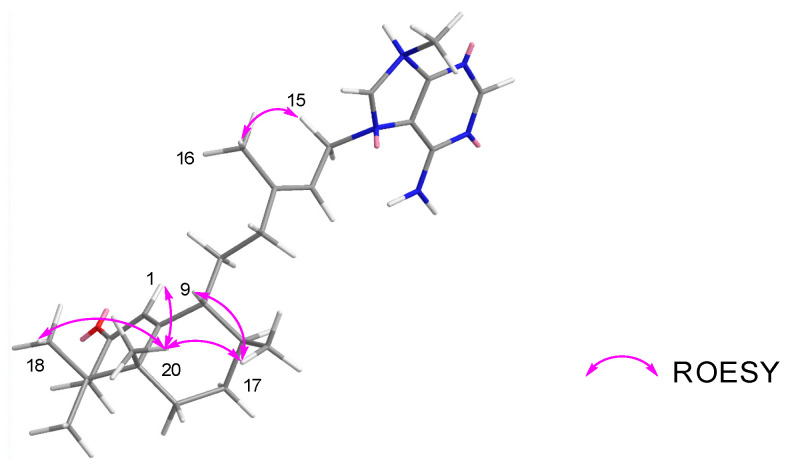
Key ROESY NMR correlations for **1** in MeCN-*d*_3_.

With the aim of improving the ^1^H NMR spectrum to facilitate more definitive ROESY-based relative configuration assignments, we undertook additional investigations of **1** in other deuterated NMR solvents (benzene-*d*_6_ and MeCN-*d*_3_). While compound **1** showed poor solubility in benzene-*d*_6_, it dissolved readily in MeCN-*d*_3_, with the resulting ^1^H NMR spectrum displaying superior signal dispersion compared to MeOH-*d*_4_ (Figure S9). Hence, COSY and ROESY NMR data were recorded in MeCN-*d*_3,_ and all proton chemical shifts were reassigned based on the analysis of the new 2D NMR data (Figures S10 and S11). Fortuitously, the ROESY NMR data in MeCN-*d*_3_ showed clear and strong correlations associated with protons situated around the decalin subunit that ultimately allowed us to resolve the relative configuration for **1**. Key ROESY correlations from methyl H_3_-17 (*δ*_H_ 0.95) to H-9 (*δ*_H_ 2.15) and methyl H_3_-20 (*δ*_H_ 1.16) suggested that they must reside on the same face of the decalin ring system. In addition, methyl H_3_-20 displayed further ROESY correlations with the alpha keto methine H-1 (*δ*_H_ 5.79), methylene proton H-6a (*δ*_H_ 2.04) and methyl H_3_-18 (*δ*_H_ 0.98), indicating that these protons also resided on the same face of **1**. These data allowed the relative configuration of the decalin moiety in **1** to be assigned as 5R*, 8S* and 9R* (Figure 5), consistent with candidate **1a** in MeOH-*d*_4_. Furthermore, the ∆^13^ geometry was assigned as *E* based on ROESY correlations between the olefinic methyl (H_3_-16, *δ*_H_ 1.80) and the methylene group (H_2_-15, *δ*_H_ 5.08).

**Figure 6:**
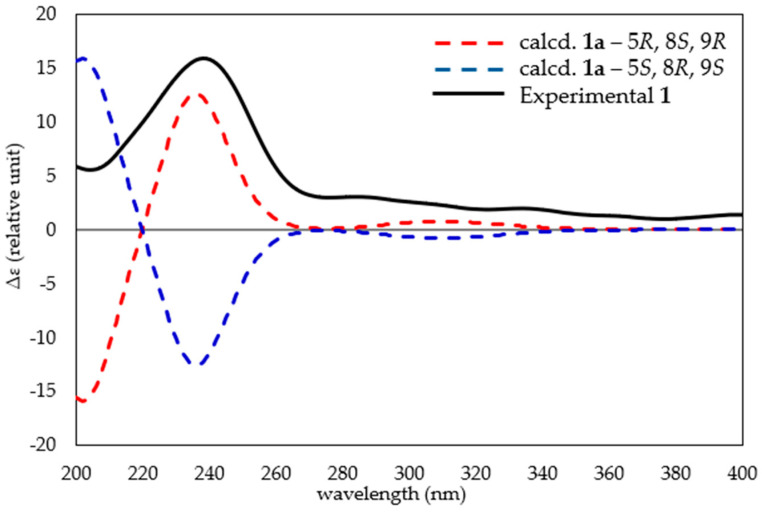
Comparative TDDFT-calculated (red dashed line **1a**—5*R*, 8*S* and 9*R*; blue dashed line **1a**—5*S*, 8*R* and 9*S*) and experimental ECD spectra for agelasine Z (**1**).

Quantum chemical NMR and time-dependent density functional theory (TDDFT) ECD calculations have found increasing utility in informing the structure elucidation of complex natural products [21,22,23]. Notably, density functional theory (DFT) techniques have been used to aid the absolute configurational assignment of related agelasines, isoagelasine C [13] and nakamusine A [20]. To support the experimentally assigned relative configuration of agelasine Z (**1**), gauge including atomic orbital (GIAO) DFT NMR chemical shift calculations were performed on truncated candidate diastereomers, **1a** and **1b** (Tables S4–S10). After DFT geometry optimization, the NMR shielding tensors for **1a** and **1b** were calculated at the mPW1PW91/6-311+G(d,p) level of theory and evaluated using DP4+ [21]. The DP4+ analysis returned 100% probabilistic support for the 5R*, 8S* and 9R* diastereomer (**1a**, Table S4), in excellent agreement with our experimentally ascribed relative configuration. Next, TDDFT ECD calculations were undertaken to examine the absolute configuration of **1**. The TDDFT-calculated ECD spectra for **1a**’s 5*R*, 8*S* and 9*R* enantiomer was found to match the experimental CD spectrum for agelasine Z (**1**, Figure 6), thereby allowing the absolute configuration of **1** to be assigned.

It is noteworthy that agelasines exhibit variability in their chiral centres, with multiple configurations observed within the same sponge material [15,24]. For example, agelasines B (**2**) and D (**6**), originally isolated from *Agelas* sp., have been reported with the absolute configurations 5*R*, 8*R*, 9*S*, 10*R* and 5*S*, 9*S*, 10*S*, respectively [14]. Given these observations, and the fact that oxoagelasine B (**3**) is a C-2 oxidized derivative of agelasine B (**2**) with matching optical rotation, it is likely that both alkaloids share the same absolute configuration.

### 2.2. Conversion of H-8’ to D-8’ of Agelasines in MeOH-d_4_

**Figure 7:**
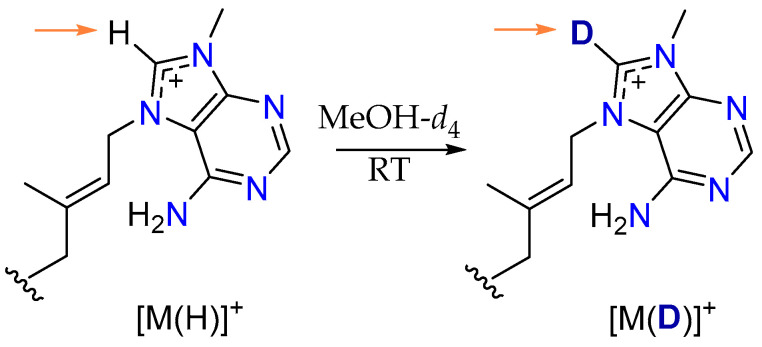
Conversion of H-8’ to D-8’ of the adenine moiety of compounds **1**–**3** and **6** during storage in MeOH-*d*_4_ at room temperature.

Deuteration is the process of replacing hydrogen atoms with their heavier isotope, deuterium (D), and has recently gained significant attention due to its potential to alter the physiochemical and biological properties of molecules [25]. Many deuteration methods involve metal catalysts [26], while in some instances, deuterated solvents trigger the reaction [27]. During this investigation, we observed a gradual decrease in the intensity of the adenine proton resonance H-8’ in agelasines **1**–**3** and **6** when NMR samples were stored in MeOH-*d*_4_ at room temperature while awaiting access to the NMR spectrometer for 1D/2D NMR data acquisition. Supported by LC-MS analysis, these data suggest that agelasines undergo deuterium exchange at H-8’ (Figure 7), with replacement by D occurring under mild storage conditions and within short timeframes (<16 h). To the best of our knowledge, this is the first report of D exchange for agelasine-type alkaloids, which is surprising given that the majority of these marine natural products have had their NMR data recorded in MeOH-*d*_4_.

**Figure 8:**
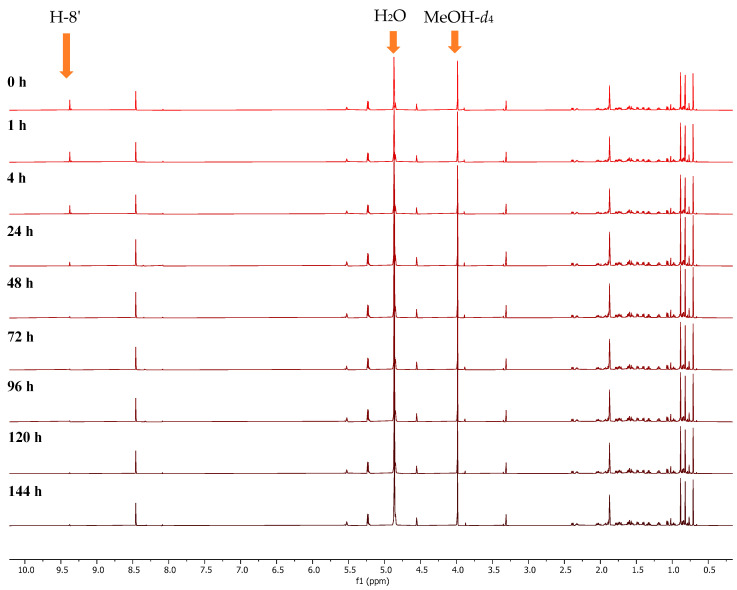
Stacked ^1^H NMR spectra of agelasine D (**6**) in MeOH-*d*_4_ at 25 °C recorded at 0, 1, 4, 24, 48, 72, 96, 120 and 144 h.

To gain a better understanding of the reaction, especially the kinetics of this conversion, we undertook more detailed studies on **6**, the most abundant agelasine available in our studies. The process began by dissolving **6** (10 mg) in MeOH-*d*_4_ (180 µL) in a 3 mm NMR tube. The ^1^H NMR spectrum of **6** was recorded at 0, 1, 4, 24, 48, 72, 96, 120 and 144 h (Figure 8), and the intensity of the H-8’ resonance was measured at each time point.

**Figure 9:**
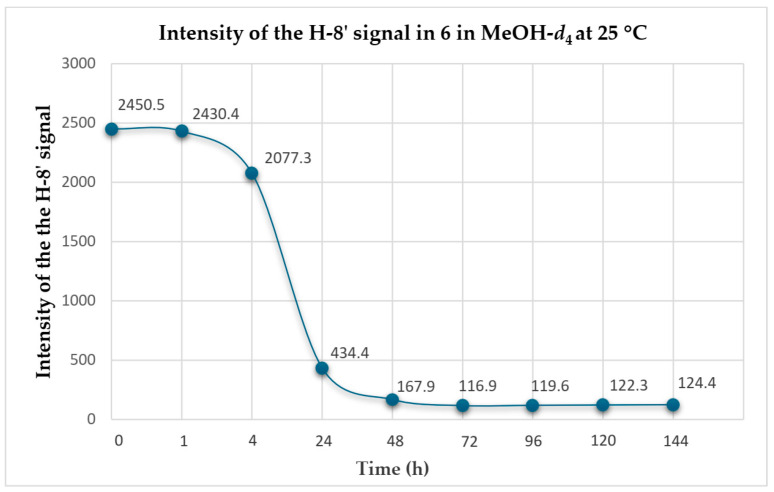
Conversion of H-8’ to D-8’ for agelasine D (**6**) in MeOH-*d*_4_ at 25 °C.

These detailed kinetic studies indicated that deuteration takes place rapidly with an almost full conversion of H-8’ to D-8’ during the first 24 h (96%), after which time there was no significant change (Figure 9).

Moreover, the addition of deuterium to agelasine D to produce compound **7** was further supported by 1D/2D NMR (Table 2) and LRESIMS data (Figure S32).

**Figure 10:**
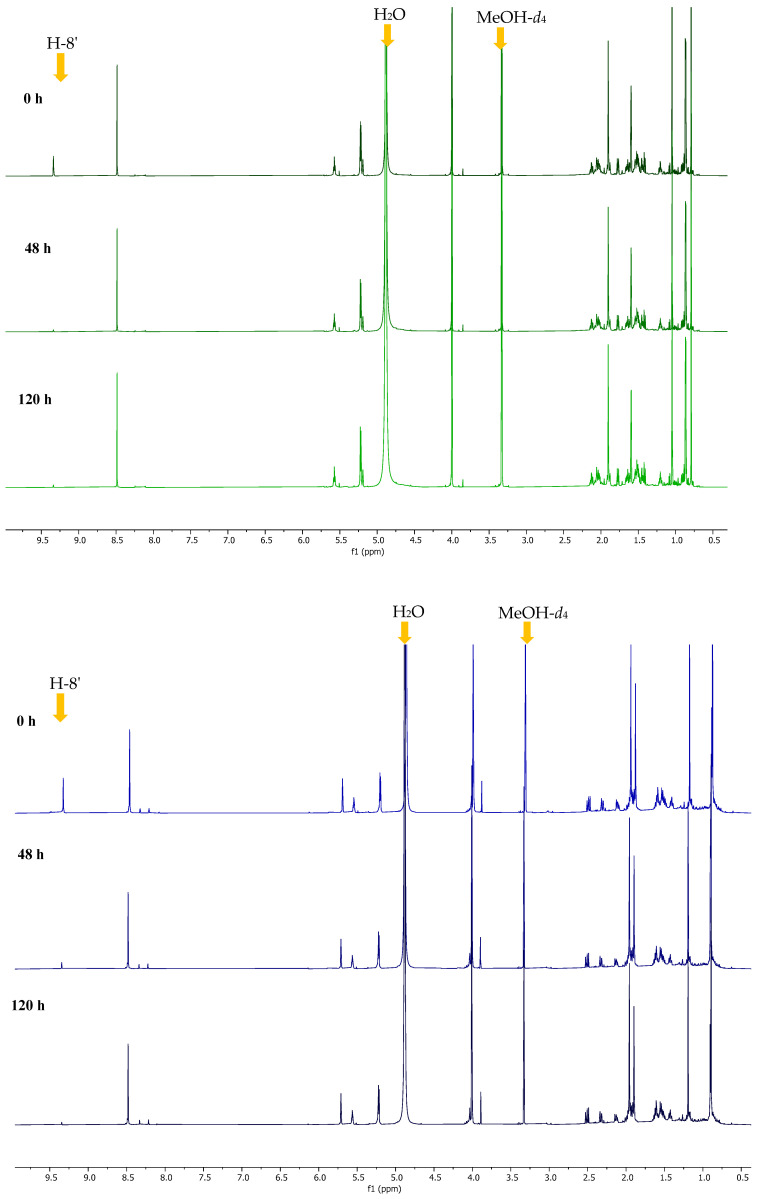
Stacked ^1^H NMR spectra of agelasine B (**2**, **top** spectra) and oxoagelasine B (**3**, **bottom** spectra) in MeOH-*d*_4_ at 25 °C recorded at 0, 48 and 120 h.

A similar trend was also observed in the deuteration of the two known compounds agelasine B (**2**) and oxoagelasine B (**3**) with the ^1^H NMR spectra in MeOH-*d*_4_ and H-8’ intensity for both compounds recorded at the 0, 48 and 120 h time points (Figure 10). Unfortunately, deuteration kinetic studies could not be performed on the new compound **1** due to the small quantities available.

**Figure 11:**
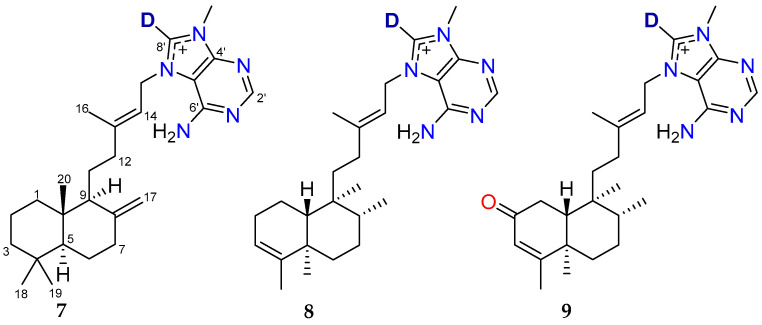
Chemical structures of deuterated compounds **7**–**9**.

The deuteration of **2** and **3** was further confirmed by LRESIMS data with the respective deuterated molecular ion observed (Figure S35 and Figure S38, respectively). The chemical structures of deuterated agelasine D (**7**), agelasine B (**8**) and oxoagelasine B (**9**) are shown below (Figure 11).

**Figure 12:**
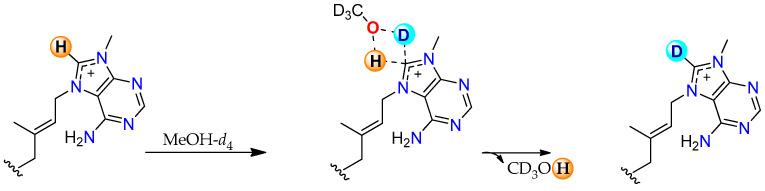
Proposed mechanism for H/D exchange of agelasines in MeOH-*d*_4_.

Based on these results, we decided to explore the mechanism by which this exchange takes place. After literature analysis, we proposed that the H-8’ bearing ring of agelasines may undergo H/D exchange as this atom is labile enough to undergo deprotonation with MeOH-*d*_4_ acting as the deuterium source [28]. There are a few research reports on the H/D exchange, and the mechanism involves a carbene intermediate [29]. Recently, Cerro et al. reported a mechanism of H/D exchange through a concerted and carbene-free intermediate supported by DFT calculations [30]. In agelasines, we hypothesize that H/D exchange occurs through an analogous transition state, mediated by H-bonded MeOH-*d*_4_ molecules (Figure 12).

In order to investigate the stability of the deuterated alkaloids, compound **7** was dissolved in MeOH-*d*_3_, and ^1^H NMR spectra were recorded at the 0, 1, 4 and 24 h time points. The lability of the proton (or deuteron) on the C-8’ carbon was apparent as over 95% D-8’ to H-8’ conversion was observed in the first 1 h. We suggest that this facile deuteration process is a general one for agelasines, and it is noteworthy that it does not appear to have been reported previously.

### 2.3. Anthelmintic Activity of Compounds ***1***–***6***

Compounds **1**–**6** were tested for their anthelmintic effect on *H. contortus* xL3s (infective) and on in vitro-raised *H. contortus* L4s (parasitic), using an established phenotypic in vitro bioassay at 100 µM [31,32]. The activity of each compound was assessed based on the reduction in larval motility (xL3 and L4) and/or the induction of an abnormal phenotype.

**Figure 13:**
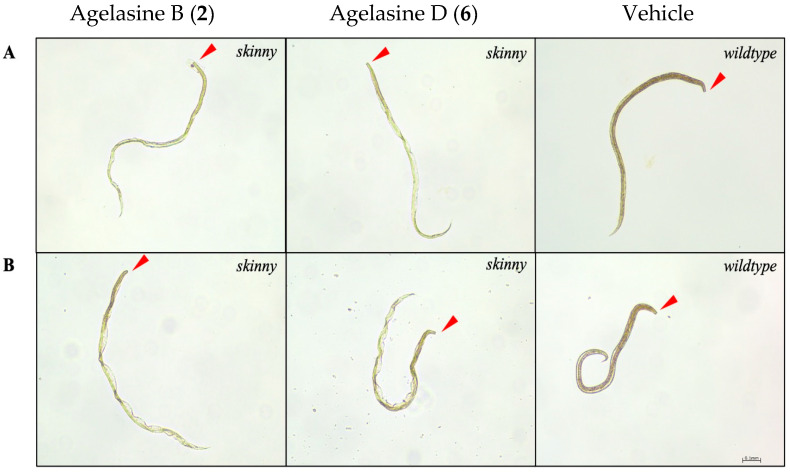
Representative images of *H. contortus* L4s following incubation with agelasine B (**2**) and agelasine D (**6**) at 100 µM displaying skinny (*Ski*) phenotype—compared with untreated control with wildtype phenotype (vehicle, containing 0.25% DMSO; right). (**A**) xL3s treated with compound for 168 h, (**B**) in vitro-raised L4s treated with compound for 90 h. Red arrow indicates anterior end of larvae. Scale bar represents 0.1 mm.

First, compounds were tested for activity on xL3s. None of the compounds (**1**–**6**) significantly reduced xL3 motility at 100 µM at 168 h. The lack of motility reduction observed for compounds **1**–**3**, which were isolated from active fractions, was anticipated since these fractions did not show a reduction in xL3 motility. Agelasine B (**2**) and agelasine D (**6**) induced a skinny (*Ski*) phenotype in 4% and 34% of affected larvae at 168 h, respectively (Figure 13A). Compounds that induce a *Ski* phenotype often exhibit effects on L4s [6,10,11,33]. Therefore, we proceeded to evaluate the activity of these compounds on the in vitro-raised L4s of *H. contortus*. Compounds **2** and **6** reduced L4 motility by 50.5% and 51.8%, respectively, at 100 µM after 90 h of incubation. Furthermore, compounds **2** and **6** induced 54% and 50% of a *Ski* phenotype in L4s (Figure 13B). While compound **1** did not reduce L4 motility, it induced a *Ski* phenotype (25%) after 90 h. Compounds **3**, **4** and **5** showed no effects on larval (xL3 or L4) motility or phenotypes.

Secondary metabolites from the genus *Agelas* exhibit antimicrobial [34,35], cytotoxic [17,36] and antiplasmodial activities [37,38]. In the literature, several agelasines have been reported to exhibit promising antiparasitic activities. Notably, agelasine D (**6**) has been shown to exhibit significant antiparasitic activity against *Plasmodium falciparum*, *Trypanosoma brucei*, *Leishmania infantum* and *Trypanosoma cruzi* [39,40]. Recently, agelasine B (**2**) isolated from a NatureBank *Agelas* sp. sample showed nematocidal activity against the model and free-living nematode *C. elegans* [41]. Thus, we assessed the activity of all six compounds (**1**–**6**) against *C. elegans* L4/young adults in an established bioassay [32]. Despite earlier findings suggesting the nematocidal activity of agelasine B (**2**) against *C. elegans*, as reported by Risi et al. in 2024 [41], the recent assessment of four compounds, including **2**, did not show them to have significant activity in reducing the motility of L4/young adult *C. elegans* after 40 h of incubation.

The observed discrepancy in the *C. elegans* assay results may be attributed to variations in experimental conditions, including differences in assay protocols, the *C. elegans* strains utilized or the range of compound concentrations tested. Such factors can significantly influence the outcomes of bioactivity studies and underscore the importance of standardized methodologies in comparative analyses.

## 3. Materials and Methods

### 3.1. General Experimental Procedures

Specific rotations were recorded using a JASCO P-2000 polarimeter (JASCO, Tokyo, Japan). ECD spectra were recorded on a JASCO J-1500 CD spectrophotometer (JASCO, Tokyo, Japan). NMR spectra were recorded at 25 °C on a Bruker AVANCE III 800 MHz NMR spectrometer (Bruker Corporation, Billerica, MA, USA). equipped with a cryoprobe. The ^1^H and ^13^C NMR chemical shifts were referenced to solvent peaks for MeOH-*d*_4_ at *δ*_H_ 3.31/*δ*_C_ 49.00 and MeCN-*d*_3_ at δ_H_ 3.31/*δ*_C_ 118.26. LRESIMS data was recorded on an Ultimate 3000 RS UHPLC (Thermo Fisher Scientific, Waltham, MA, USA) connected with a Thermo Scientific Accurose C_18_-bonded silica column (2.6 μm, 80 Å, 150 × 2.1 mm) coupled to a Thermo Fisher Scientific ISQEC single quadruple ESI mass spectrometer (Thermo Fisher Scientific, Waltham, MA, USA). HRESIMS data were acquired on a Bruker maXis II ETD ESI-qTOF mass spectrometer (Bruker Corporation, Billerica, MA, USA). GRACE Davisil (35–70 µm, 60 Å) C_18_-bonded silica was used for pre-adsorption work before reversed-phase (RP) HPLC separations. The pre-adsorbed material was subsequently packed into a GRACE stainless steel guard cartridge (10 × 30 mm) and then attached to an HPLC column prior to fractionation. A Waters 600 pump fitted with a Waters 996 photodiode array detector (Milford, MA, USA) fitted with a Gilson 717-plus autosampler was used for RP-HPLC separations (Middleton, WI, USA). Thermo Electron Betasil C_18_-bonded silica (5 μm, 100 Å, 150 × 21.2 mm) or Phenomenex Luna C_18_-bonded silica (5 μm, 100 Å, 250 × 10 mm) columns were used for RP-HPLC separations. Frozen marine biota was dried using a Dynamic FD12 freeze dryer (Buchi, Vineyard, NSW, AUS) and ground using a Fritsch Universal Cutting Mill Pulverisette 19 (FRITSCH Milling, Pittsboro, NC, USA). Solvents were removed with a Buchi R-144 rotary evaporator (Buchi, Billerica, MA, USA) and from HPLC fractions using a GeneVac XL4 centrifugal evaporator (Biopharma, Ipswich, UK). All solvents used for chromatography, UV, MS, ECD and [α]_D_ were Honeywell Burdick & Jackson or Lab-Scan HPLC-grade. H_2_O was filtered using a Sartorius Stedium Arium^®^ Pro VF ultrapure water system (Sartorius, Göttingen, Germany). All NMR spectra were processed using MestReNova version 14.3.0 (Mestrelab Research, Santiago de Compostela, Spain) software. All chemical structures were drawn using ChemDraw version 23.1.1.3 software (Revvity, Waltham, MA, USA). All HPLC and LC-MS results were analyzed by Waters Millennium^32^ version 4.0 and Chromeleon™ 7.2 software, respectively. 

### 3.2. Animal Material

The sponge *Agelas axifera* was collected on the 25th of January 1999 from Stanley Reef, Great Barrier Reef, Australia. A voucher specimen (G314522) was deposited at the Queensland Museum, South Brisbane, Queensland, Australia.

### 3.3. Extraction and Isolation

For initial anthelmintic bioassay-guided fractionation, the freeze-dried and ground specimen of *A. axifera* (1 g) was extracted sequentially with *n*-hexane (21 mL), CH_2_Cl_2_:MeOH (8:2, 21 mL) and MeOH (39 mL); the *n*-hexane extract was discarded as it only contained highly lipophilic fatty acid-derived material, while the CH_2_Cl_2_ and MeOH extracts were combined and dried under reduced pressure to produce a dark brown gum-like extract (226.1 mg). A portion of this extract (203 mg) was pre-adsorbed to C_18_-bonded silica (1 g), then packed into a guard cartridge and subjected to semipreparative HPLC using a Betasil C_18_-bonded silica HPLC column. Isocratic solvent conditions of 90% H_2_O (0.1% TFA)/10% MeOH (0.1% TFA) were initially employed for the first 10 min; then a linear gradient to 100% MeOH (0.1% TFA) was run over 40 min, followed by isocratic conditions of 100% MeOH (0.1% TFA) for a further 10 min, all at a flow rate of 9 mL/min. Sixty fractions were collected from 0 to 60 min (60 × 1 min fractions), concentrated in a centrifugal evaporator and prepared for anthelmintic screening. Fractions 49 (F49) and 50 (F50) that displayed eviscerated phenotypes in xL3 were combined (16.3 mg) and subsequently purified via semipreparative HPLC using a Luna C_18_-bonded silica HPLC column. Isocratic solvent conditions of 50% H_2_O (0.1% TFA)/50% MeOH (0.1% TFA) were initially employed for the first 10 min; then a linear gradient to 100% MeOH (0.1% TFA) was run over 40 min, followed by isocratic conditions of 100% MeOH (0.1% TFA) for a further 10 min, all at a flow rate of 4 mL/min to afford the TFA salts of agelasine Z (**1**, 0.9 mg, *t*_R_ 19–20 min, 0.009% dry wt), oxoagelasine B (**3**, 1.0 mg, *t*_R_ 22–23 min, 0.01% dry wt) and agelasine B (**2**, 1.5 mg, *t*_R_ 35–36 min, 0.015% dry wt).

In order to obtain more of the agelasine-type compounds and potentially other pure compounds, an additional 5 g of freeze-dried sponge sample was extracted with CH_2_Cl_2_:MeOH (80:20, 125 mL, 2 h) and MeOH (125 mL, 2 h). Both extracts were combined and dried under reduced pressure to give a dark brown gum extract (1.74 g). The crude extract was subjected to RP-HPLC using Betasil C_18_-HPLC with isocratic solvent conditions of 90% H_2_O (0.1% TFA)/10% MeOH (0.1% TFA) for the first 10 min, then a linear gradient to 100% MeOH (0.1% TFA) over 40 min, followed by isocratic conditions of 100% MeOH (0.1% TFA) for a further 10 min, all at a flow rate of 9 mL/min to afford 60 fractions. (–)-Mukanadin C (**4**, 6.1 mg, 0.12% dry wt) and 4-bromopyrrole-2-carboxylic acid (**5**, 14.5 mg, 0.29% dry wt) were isolated at *t*_R_ 25–26 min and *t*_R_ 28–29 min, respectively. Fractions containing targeted agelasines (F49 and F50, 118.8 mg) were combined and repurified using Betasil C_18_-bonded silica HPLC using 50% H_2_O (0.1% TFA)/50% MeOH (0.1% TFA) the first 10 min followed by a linear gradient to 100% MeOH (0.1% TFA) over 40 min and then isocratic conditions of 100% MeOH (0.1% TFA) for a further 10 min, all at a flow rate of 9 mL/min to afford additional agelasine Z (**1**, 2.6 mg, *t*_R_ 21–22 min, 0.052% dry wt), oxoagelasine B (**3**, 16.7 mg, *t*_R_ 25–27 min, 0.33% dry wt) and agelasine B (**2**, 41.2 mg, *t*_R_ 35–40 min, 0.82% dry wt).

Agelasine Z (**1**): pale yellow gum; ${\left[\alpha \right]}_{D}^{26}$ –16.5 (*c* 0.09, MeOH); UV (MeOH) λmax (log ε) 208 (0.30), 274 (0.08) nm; ECD (MeOH) *λ*_ext_ 242 nm (Δε +15.8), 291 (Δε +2.9); ^1^H and ^13^C NMR data, see Table 1 and Table S1; LRESIMS *m*/*z* 436 [M]^+^; HRESIMS *m*/*z* 436.3060 [M]^+^ (calcd for C_26_H_38_N_5_O, 436.3071).

Agelasine B (**2**): white amorphous solid; ${\left[\alpha \right]}_{D}^{26}$ –11.6 (*c* 0.06, MeOH), lit. ${\left[\alpha \right]}_{D}^{20}$ –21.5 (*c* 0.1, MeOH) [14]; ECD (MeOH) *λ*_ext_ 288 nm (Δε +6.8); ); ^1^H and ^13^C NMR spectra, see Figures S12 and S13; LRESIMS *m*/*z* 422 [M]^+^.

Oxoagelasine B (**3**): pale yellow gum; ${\left[\alpha \right]}_{D}^{26}$ –11.6 (*c* 0.01, MeOH), lit. ${\left[\alpha \right]}_{D}^{20}$—8.7 (*c* 0.3, MeOH) [15]; ECD (MeOH) *λ*_ext_ 220 nm (Δε +11.7), 248 (Δε −6.1), 272 nm (Δε +1.7), 289 nm (Δε −0.5), 326 nm (Δε +4.0); ^1^H and ^13^C NMR spectra, see Figures S14 and S15; LRESIMS *m*/*z* 436 [M]^+^.

(–)-Mukanadin C (**4**): amorphous white solid; ${\left[\alpha \right]}_{D}^{26}$ −10.7 (*c* 0.1, MeOH), lit. ${\left[\alpha \right]}_{D}^{20}$—19 (*c* 1.9, MeOH) [16]; ^1^H and ^13^C NMR spectra, see Figures S19 and S20; LRESIMS *m*/*z* 231 [M–^79^Br]^+^ and 233 [M–^81^Br]^+^.

4-Bromopyrrole-2-carboxylic acid (**5**): amorphous white solid; ^1^H and ^13^C NMR spectra, see Figures S21 and S22; LRESIMS *m*/*z* 189 [M–^79^Br]^+^ and 191 [M–^81^Br]^+^.

Agelasine D (**6**): amorphous white solid; ${\left[\alpha \right]}_{D}^{26}$ +12.9 (*c* 0.2, MeOH), lit. ${\left[\alpha \right]}_{D}^{20}$ + 15.4 (*c* 0.02, MeOH)15; ^1^H and ^13^C NMR spectra, see Figures S16 and S17; LRESIMS *m*/*z* 422 [M]^+^.

Deuterated agelasine D (**7**): pale yellow gum; ^1^H and ^13^C NMR data, see Table 2 and Table S3; LRESIMS *m*/*z* 423 [M(D)]^+^.

Deuterated agelasine B (**8**): pale yellow gum; ^1^H and ^13^C NMR spectra, see Figures S33 and S34; LRESIMS *m*/*z* 423 [M(D)]^+^.

Deuterated oxoagelasine B (**9**): pale brown gum; ^1^H and ^13^C NMR spectra, see Figures S36 and S37; LRESIMS *m*/*z* 437 [M(D)]^+^.

### 3.4. Production and Maintenance of H. contortus for Bioassays

The Haecon-5 strain of *H. contortus* was maintained in experimental sheep according to established protocols [42,43], in compliance with institutional animal ethics guidelines (permit no. 23983; University of Melbourne) and Australian regulations. Helminth-free Merino sheep (six months old; male) were orally inoculated with 7000 third-stage larvae (L3s) of *H. contortus*. Fecal samples, containing *H. contortus* eggs, were collected daily from sheep with patent infection, commencing four weeks post-infection. These eggs were incubated at 27 °C and >90% relative humidity for seven days to develop into L3 larvae. The developed larvae were then collected, suspended in tap water and filtered through two layers of nylon mesh (pore size: 20 µm; Sefar, Australia) to eliminate debris or dead larvae. Filtered larvae were stored in 0.9% NaCl saline solution (at a density of 2000 L3s per mL) at 11 °C for up to six months [33]. Immediately prior to use, L3s were exsheathed and sterilized by incubation in 0.15% (*v*/*v*) sodium hypochlorite (NaClO) at 38 °C for 20 min with gentle shaking [43]. Post-treatment, exsheathed L3s (xL3s) were immediately washed five times in 50 mL of sterile saline by centrifugation at 800× *g* (5 min) at room temperature (22–24 °C). Following the final wash, xL3s were suspended in sterile lysogeny broth (LB) [44] supplemented with 100 IU/mL of penicillin, 100 µg/mL of streptomycin and 0.25 µg/mL of amphotericin B (Fungizone^®^, Thermo Fisher Scientific, Waltham, MA, USA)—referred to as LB*. To develop in vitro fourth-stage larvae (L4) of *H. contortus*, xL3s were incubated at 38 °C, 10% (*v*/*v*) CO_2_ and >90% relative humidity for 168 h prior to incubation with each compound [6].

### 3.5. Production and Synchronization of C. elegans for Bioassays

The N2 strain (Bristol; wildtype) of *C. elegans* was cultured under standard laboratory conditions at 20 °C on nematode growth medium (NGM) agar plates seeded with *Escherichia coli* OP50 as a food source [45,46]. Gravid adult worms were harvested from NGM plates, washed with sterile M9 buffer and then treated with a sterile 0.4% (*v*/*v*) NaOCl solution with 170 mM sodium hydroxide for 4–8 min at room temperature (22–24 °C) to release eggs, with intermittent shaking [46,47]. Upon release, eggs were immediately collected and washed five times in 15 mL of sterile M9 buffer by centrifugation at 500× *g* for 2 min. Washed eggs were suspended in 8 mL of sterile M9 buffer and incubated in a 15 mL tube with gentle agitation for 24 h at room temperature (22–24 °C), allowing first-stage larvae (L1s) to hatch and enter diapause. Approximately 36 h before setting up assays, synchronized L1s were transferred to NGM agar plates (10 cm) previously seeded with 500 µL of *E. coli* OP50 (~3000 larvae per plate) and allowed to synchronously develop to fourth-stage larvae (L4s) at 20 °C. On the day of assay, L4s were collected from NGM agar plates and washed twice with sterile M9 buffer by centrifugation at 500× g (2 min) to remove *E. coli* OP50 and then resuspended in LB*.

### 3.6. Bioassay for Assessment of Anthelmintic Activity of A. axifera-Derived HPLC Fractions

Individual HPLC fractions (*n* = 60) were tested for their anthelmintic effect on the larvae (xL3s) of *H. contortus* using an established bioassay [11]. The assay was performed in triplicate. In brief, fractions were diluted to 12 μge/μL in LB*, and 40 μL of diluted fractions was dispensed into the wells of sterile 368-well flat-bottom microtiter plates (cat. no. 3680; Corning, Corning, NY, USA) containing 80 xL3s; quadruplicate wells with no compound (LB* + 0.6% DMSO; negative control) or monepantel (Zolvix™; Elanco, Greenfield, IN, USA), moxidectin (Cydectin®; Virbac, Carros, France) and compound MIPS-0018666 (abbreviated here as M-666) [48] were used as positive controls (20 µM). The motility of xL3s was measured at 90 h, and the development and phenotypic alterations of xL3s were assessed at 168 h. At 168 h, larvae in individual wells were fixed with 40 µL of 1% iodine and microscopically examined (using an M80 light microscope; Leica, Wetzlar, Germany) at 60-times magnification to assess their development morphology (phenotype) [9,32].

### 3.7. Bioassay for Evaluation of Anthelmintic Activity of Pure Compounds Using Larvae of H. contortus or C. elegans

These evaluations were carried out for compounds **1**–**6** against *H. contortus* xL3s/L4 or *C. elegans* L4s with reference to the two positive control compounds (monepantel and moxidectin) using established assays [6,9]. *H. contortus* xL3s/L4s or *C. elegans* L4s were dispensed in 96-well plates (cat. no. 3596; Corning, USA) at a density of 300 *H. contortus* xL3s/L4s or 100 *C. elegans* L4s per well, respectively, with individual, serially diluted compounds (9 points; 2-fold serial dilution in LB*; 100 µM to 0.39 nM), in a final volume of 100 µL of LB*. Plates containing *H. contortus* xL3s/L4s were incubated at 38 °C, 10% (*v*/*v*) CO_2_ and >90% relative humidity. Plates containing *C. elegans* L4s were incubated at 20 °C. Compounds were tested by three independent experiments in triplicate. The motility of *H. contortus* xL3s and L4s was measured following 168 h and 90 h of incubation with the compound, respectively. The motility of *C. elegans* young adults was measured following 40 h of incubation with the compound. A one-way analysis of variance (ANOVA) with Tukey’s multiple comparison test or an unpaired *t*-test was used to establish statistically significant differences in larval motility.

### 3.8. Computational Methods

Conformer searches for GIAO NMR and TDDFT ECD calculations were performed using the Schrödinger Macromodel software (version 10.7) software suite, Monte Carlo Minimum method (MCMM) molecular mechanics, MMFF forcefield and a 21.0 kJ/mol energy window. The step count for establishing accurate conformer representation was set so that all low-energy conformers were found at least 10 times. For GIAO DFT NMR calculations, each conformer sets for candidate structures **1a** and **1b** underwent an initial gas-phase geometry optimization (GO) at the B3LYP/6-31G* level of theory using Gaussian 16 (Revision C.01) [49]. The resulting GO sets were filtered for duplicate and high-energy conformers (>3.0 kcal/mol above the energy minimum removed, Tables S5–S8). ^1^H and ^13^C NMR chemical shifts were calculated at the mPW1PW91/6-311+G(d,p) level of theory with the PCM solvent model for methanol [50]. The Boltzmann-averaged NMR data for each candidate stereoisomer were then analyzed using the Bayesian probabilistic DP4+ method [21]. For TDDFT ECD calculations, two DFT geometry optimization (GO) calculations were performed on **1** using Gaussian 16 software. The first GO was calculated at the B3LYP-31G(d) level of theory in the gas phase, while the second GO and frequency calculation were performed at the higher B3LYP/6-311G(d,p) level of theory with the PCM solvent model for methanol. The second GO set was filtered for imaginary frequencies, redundant conformers and energies > 3.0 kcal/mol (Tables S9 and S10). Following this, electronic transition and rotational strength calculations were performed using the TDDFT method at the CAM-B3LYP/6-311+G(d,p) level of theory (inclusive of the PCM solvent model for methanol) within Gaussian 16. The resultant TDDFT-calculated UV and ECD spectra for **1** were Boltzmann-weighted and visualized using the freely available SpecDis software (version 1.71) [51]. TDDFT-calculated and experimental UV spectra for **1** were matched to accurately align experimental and TDDFT-calculated ECD spectra. A Gaussian band shape and sigma gamma value of 0.29 eV and a +5nm UV correction were applied to experimental spectra for **1**. Automated processes with the HPC were performed using a Windows 11 PC with modified Python scripts originally sourced from the Willoughby protocol [52]. SpectroIBIS (v1.01) was used to scale NMR shielding tensors and to generate tabulated DFT energies and output data [53].

## 4. Conclusions

In summary, the bioassay-guided fractionation of an *A. axifera* extract afforded one new and two known agelasine alkaloids. Despite the initial activity observed in the crude extract, neither the fractions nor the isolated compounds demonstrated significant activity in subsequent assays. A challenge associated with the isolation of natural products from bioactive extracts is the loss of activity or inability to isolate the bioactive compounds during bioassay-guided fractionation. Several factors may potentially contribute to this issue, including the degradation of the bioactive constituents during purification, insufficient amounts of compounds for effective separation and/or the observed activity actually being a result of the synergistic interaction of several different compounds [54]. The current findings from our research suggest that bioactivity may be attributed to the synergistic effects of the compounds within the fractions. Whilst additional studies could explore potential synergistic interactions and optimize the isolation process to identify other minor and active constituents, limited sponge material currently prevents such investigations. Finally, it is interesting to note that the H-8’ of the adenine moiety for the three agelasines studied here was unexpectedly labile and underwent a facile deuterium exchange reaction in MeOH-*d*_4_ at room temperature.

## Figures and Tables

**Table 1:** ^1^H (800 MHz) and ^13^C (200 MHz) NMR data for the TFA salt of agelasine Z (**1**) in MeOH-*d*_4_.

Position	*δ*_C_, Type	*δ*_H,_ mult., (*J* in Hz)
1	122.6, CH	5.87, s
2	202.7, C	-
3a	47.8, CH_2_	2.42, d (16.1)
3b		1.98, d (16.1)
4	35.4, C	-
5	46.1, C	-
6a	31.0, CH_2_	2.11, m
6b		1.43, m
7a	31.6, CH_2_	1.64, m
7b		
8	47.5, CH	1.48, m
9	47.2, CH	2.19, dd (12.8, 3.7)
10	176.4, C	-
11a	31.0, CH_2_	2.08, m
11b		1.50, m
12a	35.2, CH_2_	2.10, m
12b		1.78, m
13	149.1, C	-
14	115.8, CH	5.55, t (7.0)
15a	48.6, CH_2_	5.21, d (7.0)
15b		
16	17.0, CH_3_	1.88, s
17	16.4, CH_3_	0.98, d (6.8)
18	27.8, CH_3_	1.01, s
19	28.8, CH_3_	1.04, s
20	23.2, CH_3_	1.20, s
2’	157.1, CH	8.47, s
4’	150.9, C	-
5’	111.2, C	-
6’	154.1, C	-
8’	141.9, CH	9.33, s
NH_2_	-	n.d.
9-*N*CH_3_	32.0, CH_3_	3.98, s

n.d.: not detected.

**Table 2:** ^1^H (800 MHz) and ^13^C (200 MHz) NMR data of deuterated agelasine D (**7**) in MeOH-*d*_4_.

Position	*δ*_C_, Type	*δ*_H,_ mult., (*J* in Hz)
1a	40.3, CH_2_	1.78, m
1b		0.98, td (12.8, 3.5)
2a	20.4, CH_2_	1.74, m
2b		1.57, m
3a	43.3, CH_2_	1.40, m
3b		1.19, td (13.5, 3.7)
4	34.5, C	-
5	57.4, CH	1.06, dd (12.6, 2.8)
6a	25.6, CH_2_	1.75, m
6b		1.33, m
7a	39.4, CH_2_	2.39, m
7b		1.93, m
8	149.7, C	-
9	56.9, CH	1.60, m
10	40.7, C	-
11a	22.6, CH_2_	1.71, m
11b		1.55, m
12a	39.4, CH_2_	2.33, m
12b		2.04, m
13	149.5, C	-
14	115.7, CH	5.52, t (7.2)
15a	48.6, CH_2_	5.23, d (7.2)
15b		
16	17.0, CH_3_	1.87, s
17a	107.0, CH_2_	4.88, m
17b		4.59, brs
18	34.1, CH_3_	0.87, s
19	22.1, CH_3_	0.82, s
20	15.0, CH_3_	0.71, s
2’	157.2, CH	8.47, s
4’	150.9, C	-
5’	111.1, C	-
6’	145.4, C	-
8’	142.0, CD	-
NH_2_	-	n.d.
9-*N*CH_3_	32.0, CH_3_	3.98, s

n.d.: not detected.

## Data Availability

The original data presented in the study are included in the article/Supplementary Materials; further inquiries can be directed to the corresponding author.

## Supplementary Materials

The following supporting information can be downloaded at https://www.mdpi.com/article/10.3390/md23070276/s1, Tables S1–S3: NMR data of compounds **1** and **7**; Table S4: DP4+ output for **1**; Tables S5–S10: Computational data for **1**; Figures S1–S38: NMR, LC-MS, HRESIMS and ECD spectra of compounds **1**–**9**.
