# Supplementary material for: Anthelmintic Potential of Agelasine Alkaloids from the Australian Marine Sponge Agelas axifera
Source: Mar Drugs. 2025 Jul 1;23(7):276. doi: 10.3390/md23070276 (PMC12299395; doi:10.3390/md23070276)
Supplement: Supplementary file 1 [file marinedrugs-23-00276-s001.zip › marinedrugs-3714838-supplementary.pdf]

Supplementary Materials

Anthelmintic Potential of Agelasine Alkaloids from the Australian Marine Sponge *Agelas axifera*

Kanchana Wijesekera,[1,2] Aya C. Taki,[3] Joseph J. Byrne,[3] Darren C. Holland,[4,5] Ian D. Jenkins,[1,2] Merrick G. Ekins,[6] Anthony R. Carroll,[1,5] Robin B. Gasser,[3]* Rohan A. Davis[1,2,7]*

[1] Institute for Biomedicine and Glycomics, Griffith University, Brisbane, QLD 4111, Australia

[2] School of Environment and Science, Griffith University, Brisbane, QLD 4111, Australia

[3] Department of Veterinary Biosciences, Melbourne Veterinary School, Faculty of Science, The University of Melbourne, Parkville, VIC 3010, Australia

[4] School of Molecular Sciences, The University of Western Australia, Crawley, WA 6009, Australia

[5] School of Environment and Science, Griffith University, Gold Coast, QLD 4222, Australia

[6] Biodiversity and Geosciences, Queensland Museum, South Brisbane BC, QLD 4101, Australia

[7] NatureBank, Griffith University, Brisbane, QLD 4111, Australia

***Robin B. Gasser** robinbg@unimelb.edu.au

***Rohan A. Davis** r.davis@griffith.edu.au

Table of Contents

Table S1 NMR data of agelasine Z (**1**) in MeOH-d_4

Table S2 NMR data for agelasine Z (**1**) in MeCN-d_3

Table S3 NMR data for deuterated agelasine D (**7**) in MeOH-d_4

Table S4 DP4+ output for DFT GIAO ^{1}H and ^{13}C NMR shielding tensors for candidate diastereomers (**1a** = isomer **1** and **1b** = isomer 2) compared with experimental NMR data for agelasine Z (**1**) recorded in MeOH-d_4 (C-1 to C-10, C-17 to C-20 and H-1 to H-9, H_3-17 to H_3-20 highlighted red)

Table S5 Agelasine Z (**1**) truncated diastereomer **1a** energies and Boltzmann Factors for geometry optimized (GO) conformers calculated at the B3LYP/6-31* level of theory for GIAO NMR calculations (relative energies >3.0 kcal/mol not shown)

Table S6 Agelasine Z (**1**) truncated diastereomer **1b** energies, and Boltzmann Factors for geometry optimized (GO) conformers calculated at the B3LYP/6-31* level of theory for GIAO NMR calculations (relative energies >3.0 kcal/mol not shown)

Table S7 Agelasine Z (**1**) truncated isomer **1a** geometry optimized (GO) conformers (x, y, z coordinates, Gibbs Free energies, % population) calculated at the B3LYP/6-31* level of theory for GIAO NMR calculations

Table S8 Agelasine Z (**1**) truncated isomer **1b** geometry optimized (GO) conformers (x, y, z coordinates, Gibbs Free energies, % population) calculated at the B3LYP/6-31* level of theory for GIAO NMR calculations

Table S9 Agelasine Z (**1a** - 5*R*, 8*S* and 9*R*) energies and Boltzmann Factors for geometry optimized (GO) truncated conformers calculated at the B3LYP/6-311G(d,p) level of theory for TDDFT ECD calculations (relative energies >3.0 kcal/mol not shown)

Table S10 Agelasine Z (**1a** - 5*R*, 8*S* and 9*R*) geometry optimized (GO) truncated conformers (x, y, z coordinates, Gibbs Free energies, % population) calculated at the B3LYP/6-311G(d,p) level of theory for TDDFT ECD calculations

Figure S1 ^{1}H NMR (800 MHz) spectrum of agelasine Z (**1**) in MeOH-d_4

Figure S2 ^{13}C NMR (200 MHz) spectrum of agelasine Z (**1**) in MeOH-d_4

Figure S3 COSY spectrum of agelasine Z (**1**) in MeOH-d_4

Figure S4 HSQC spectrum of agelasine Z (**1**) in MeOH-d_4

Figure S5 HMBC spectrum of agelasine Z (**1**) in MeOH-d_4

Figure S6 ROESY spectrum of agelasine Z (**1**) in MeOH-d_4

Figure S7 LC-MS data of agelasine Z (**1**)

Figure S8 HRESIMS data of agelasine Z (**1**)

Figure S9 ^{1}H NMR (800 MHz) spectrum of agelasine Z (**1**) in MeCN-d_3

Figure S10 COSY spectrum of agelasine Z (**1**) in MeCN-d_3

Figure S11 ROESY spectrum of agelasine Z (**1**) in MeCN-d_3

Figure S12 ^{1}H NMR (800 MHz) spectrum of agelasine B (**2**) in MeOH-d_4

Figure S13 ^{13}C NMR (200 MHz) spectrum of agelasine B (**2**) in MeOH-d_4

Figure S14 ^{1}H NMR (800 MHz) spectrum of oxoagelasine B (**3**) in MeOH-d_4

Figure S15 ^{13}C NMR (200 MHz) spectrum of oxoagelasine B (**3**) in MeOH-d_4

Figure S16 ^{1}H NMR (800 MHz) spectrum of agelasine D (**6**) in MeOH-d_4

Figure S17 ^{13}C NMR (200 MHz) spectrum of agelasine D (**6**) in MeOH-d_4

Figure S18 ECD spectra of compounds **1-3** and **6**

Figure S19 ^{1}H NMR (800 MHz) spectrum of mukanadin C (**4**) in MeOH-d_4

Figure S20 ^{13}C NMR (200 MHz) spectrum of mukanadin C (**4**) in MeOH-d_4
Figure S21 ^{1}H NMR (800 MHz) spectrum of 4-bromopyrrole-2-carboxylic acid (**5**) in MeOH-d_4
Figure S22 ^{13}C NMR (200 MHz) spectrum of 4-bromopyrrole-2-carboxylic acid (**5**) in MeOH-d_4
Figure S23 Stacked ^{1}H NMR spectra of agelasine D (**6**) in MeOH-d_4 at 25 °C recorded at 0, 1, 4, 24, 48, 72, 96, 120 and 144 h
Figure S24 Stacked ^{1}H NMR spectra of agelasine B (**2**) in MeOH-d_4 at 25 °C recorded at 0, 48 and 120 h
Figure S25 Stacked ^{1}H NMR spectra of oxoagelasine B (**3**) in MeOH-d_4 at 25 °C recorded at 0, 48 and 120 h
Figure S26 ^{1}H NMR (800 MHz) spectrum of deuterated agelasine D (**7**) in MeOH-d_4
Figure S27 ^{13}C NMR (200 MHz) spectrum of deuterated agelasine D (**7**) in MeOH-d_4
Figure S28 COSY spectrum of deuterated agelasine D (**7**) in MeOH-d_4
Figure S29 HSQC spectrum of deuterated agelasine D (**7**) in MeOH-d_4
Figure S30 HMBC spectrum of deuterated agelasine D (**7**) in MeOH-d_4
Figure S31 ROESY spectrum of deuterated agelasine D (**7**) in MeOH-d_4
Figure S32 LC-MS data of deuterated agelasine D (**7**)
Figure S33 ^{1}H NMR (800 MHz) spectrum of deuterated agelasine B (**8**) in MeOH-d_4
Figure S34 ^{13}C NMR (200 MHz) spectrum of deuterated agelasine B (**8**) in MeOH-d_4
Figure S35 LC-MS data of deuterated agelasine B (**8**)
Figure S36 ^{1}H NMR (800 MHz) spectrum of deuterated oxoagelasine B (**9**) in MeOH-d_4
Figure S37 ^{13}C NMR (200 MHz) spectrum of deuterated oxoagelasine B (**9**) in MeOH-d_4
Figure S38 LC-MS data of deuterated oxoagelasine B (**9**)

Table S1 ^{1}H (800 MHz) and ^{13}C (200 MHz) NMR data for the TFA salt of agelasine Z (**1**) in MeOH-d_4.

Position	δ_C, type	δ_H, mult., (J in Hz)	COSY	HMBC	ROESY
1	122.6, CH	5.87, s	-	9	20
2	202.7, C	-	-	-	-
3a	47.8, CH_2	2.42, d (16.1)	3b	2	-
3b		1.98, d (16.1)	3a	2	-
4	35.4, C	-	-	-	-
5	41.6, C	-	-	-	-
6a	31.0, CH_2	2.11, m	7a, 7b	5, 8, 10, 20	-
6b		1.43, m	7a, 7b		-
7a	31.6, CH_2	1.64, m	6a, 6b, 8	5, 8, 9	-
7b			6a, 6b, 8		-
8	47.5, CH	1.48, m	7a, 7b, 9	9, 10	9, 19
9	47.2, CH	2.19, dd (12.8, 3.7)	8, 11a, 11b	10, 11	-
10	176.4, C	-	-	-	-
11a	31.0, CH_2	2.08, m	9, 12a, 12b	8, 10, 13	-
11b		1.50, m	9, 12a, 12b		-
12a	35.2, CH_2	2.10, m	11a, 11b	14, 16	-
12b		1.78, m	11a, 11b		-
13	149.1, C	-	-	-	-
14	115.8, CH	5.55, t (7.0)	15a, 15b	12, 15, 16	-
15a	48.6, CH_2	5.21, d (7.0)	14	13, 14, 5', 8',	-
15b					-
16	17.0, CH_3	1.88, s	-	-	-
17	16.4, CH_3	0.98, d (6.8)	8	7, 9	11b, 20
18	27.8, CH_3	1.01, s	-	3, 4, 5, 19	-
19	28.8, CH_3	1.04, s	-	3, 4, 5, 18	-
20	23.2, CH_3	1.20, s	-	5, 6, 10, 18, 19	-
2'	157.1, CH	8.47, s	-	4', 6'	1, 18
4'	150.9, C	-	-	-	-
5'	111.2, C	-	-	-	-
6'	154.1, C	-	-	-	-
8'	141.9, CH	9.33, s	-	4', 6'	-
NH_2	-	n.d.	-	-	-
9-*N*CH_3	32.0, CH_3	3.98, s	-	4', 8'	-

n.d.: not detected

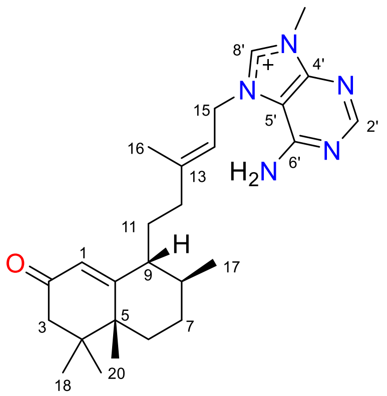

Figure S1 ^{1}H NMR (800 MHz) spectrum of agelasine Z (**1**) in MeOH-d_4

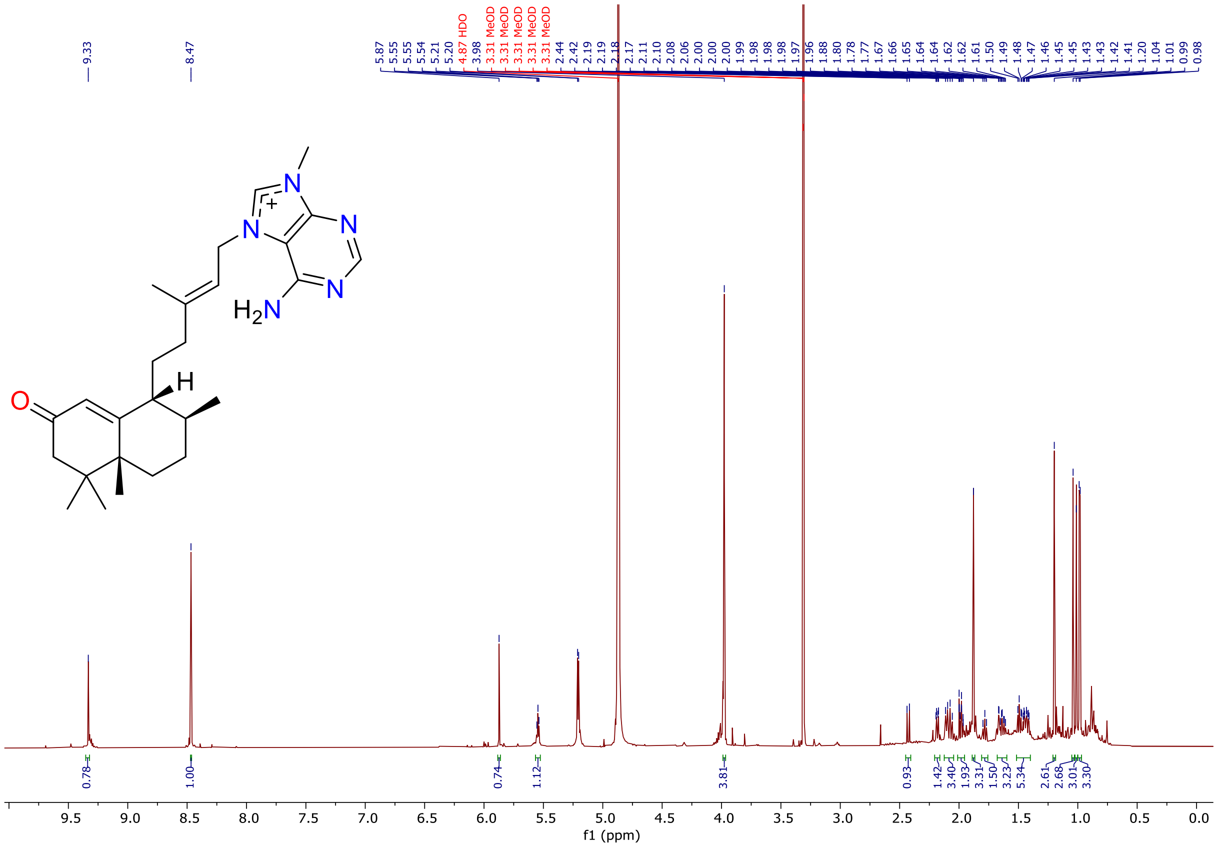

Figure S2 ^{13}C NMR (200 MHz) spectrum of agelasine Z (**1**) in MeOH-d_4

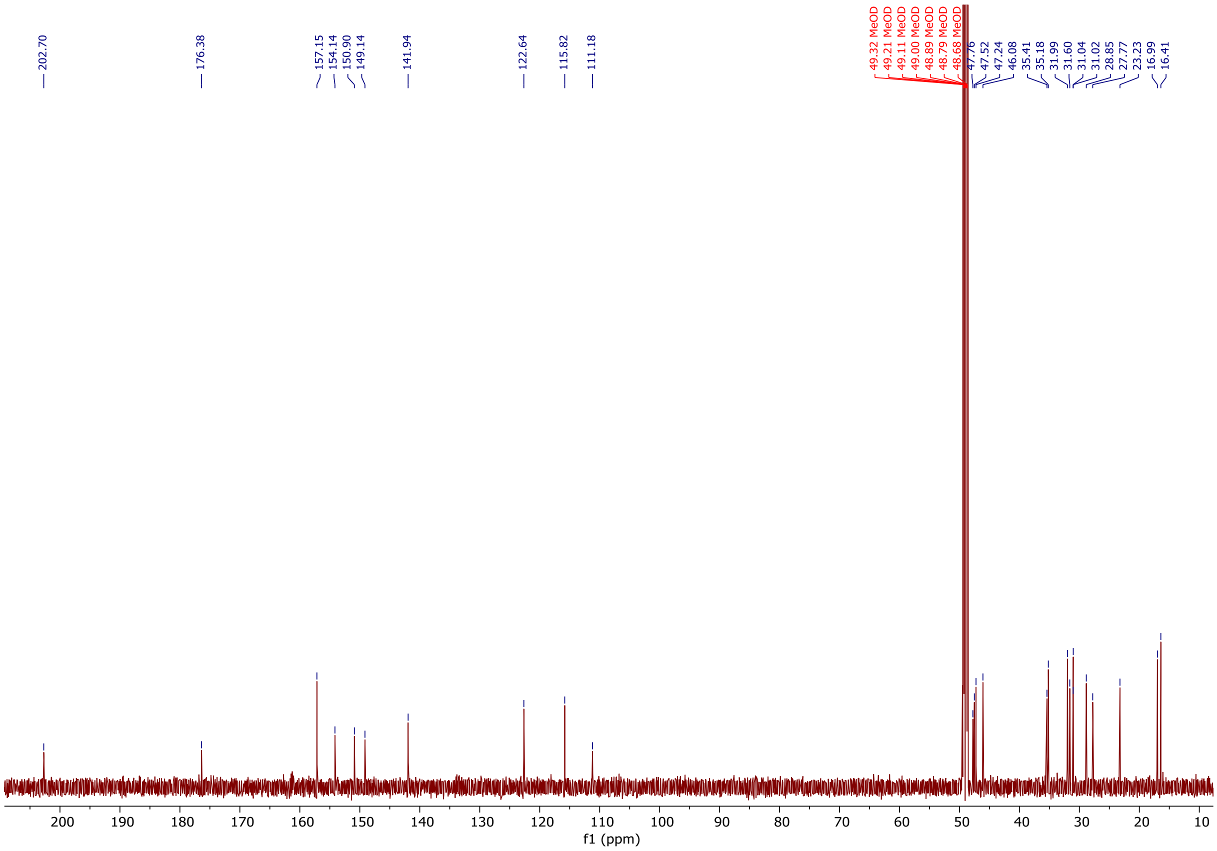

Figure S3 COSY spectrum of agelasine Z (**1**) in MeOH-d_4

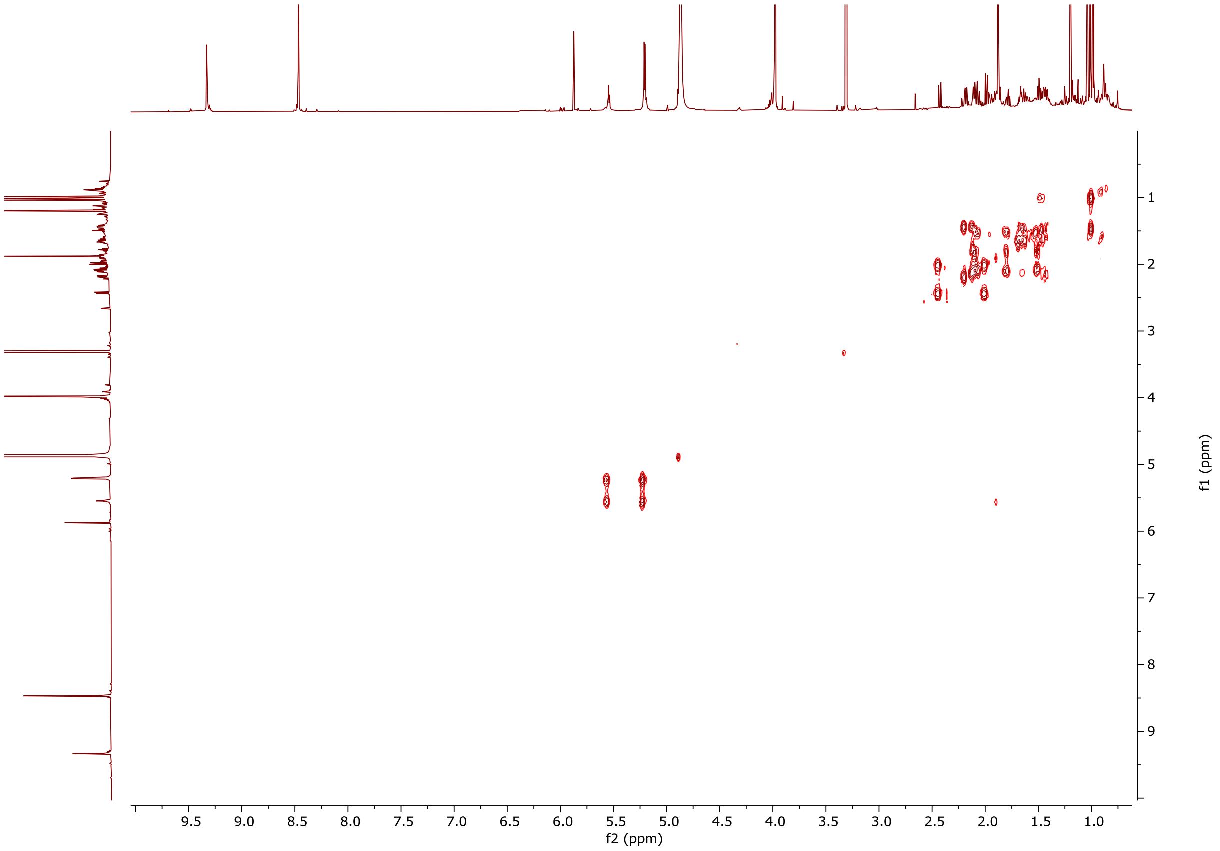

Figure S4 HSQC spectrum of agelasine Z (**1**) in MeOH-d_4

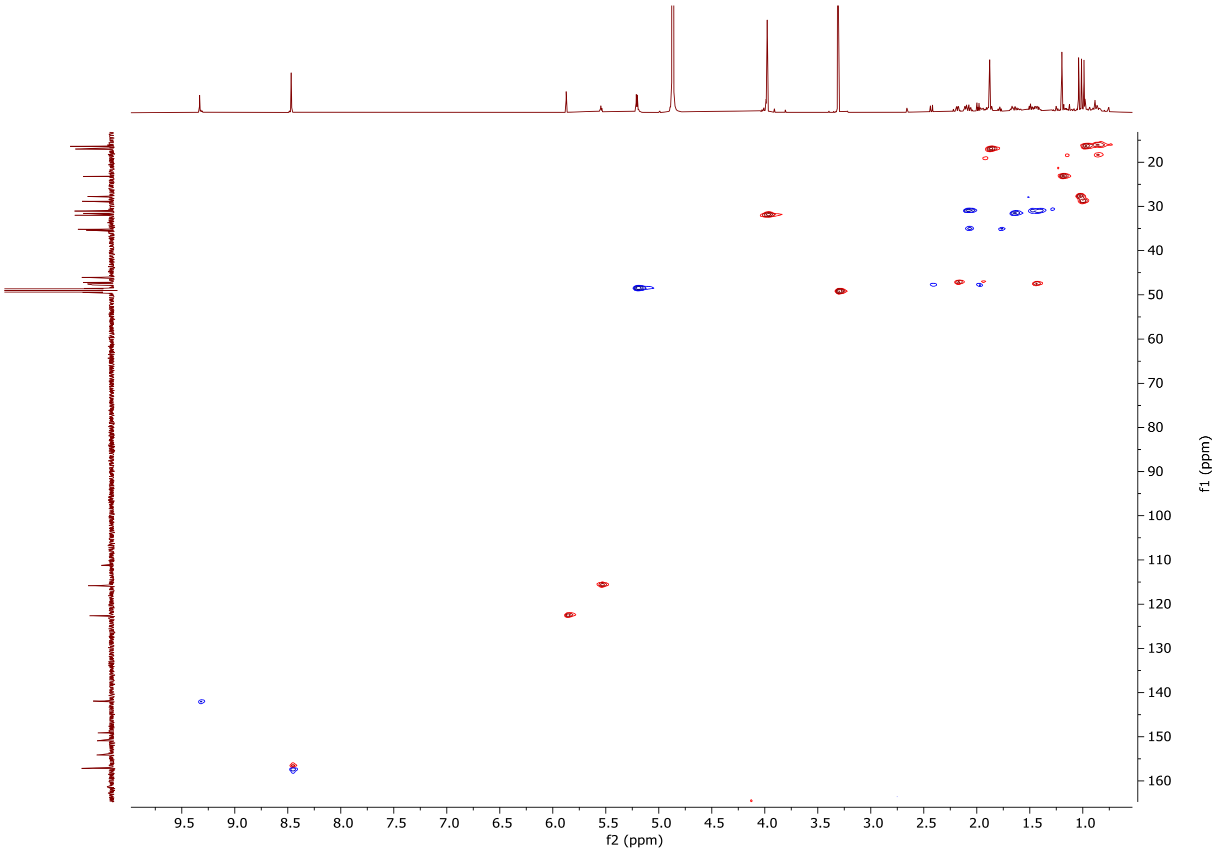

Figure S5 HMBC spectrum of agelasine Z (**1**) in MeOH-d_4

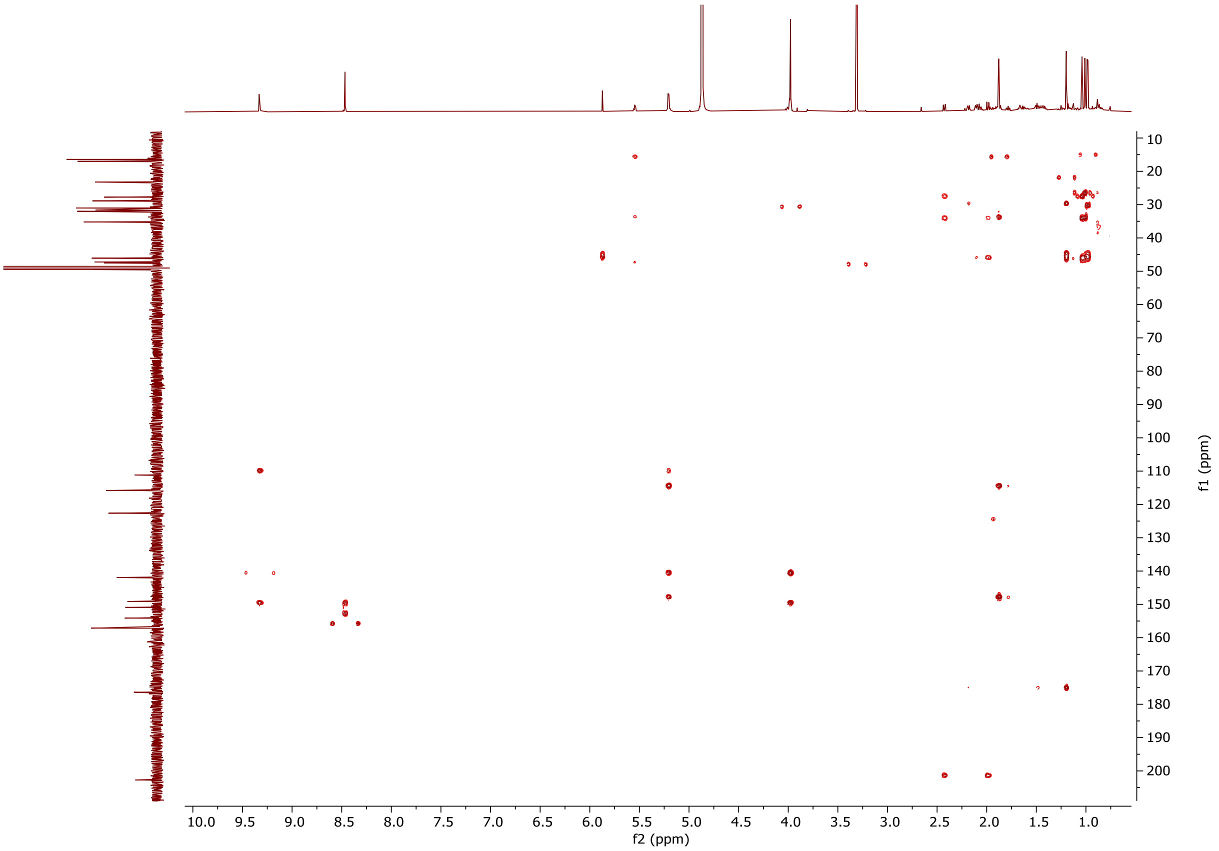

Figure S6 ROESY spectrum of agelasine Z (**1**) in MeOH-d_4

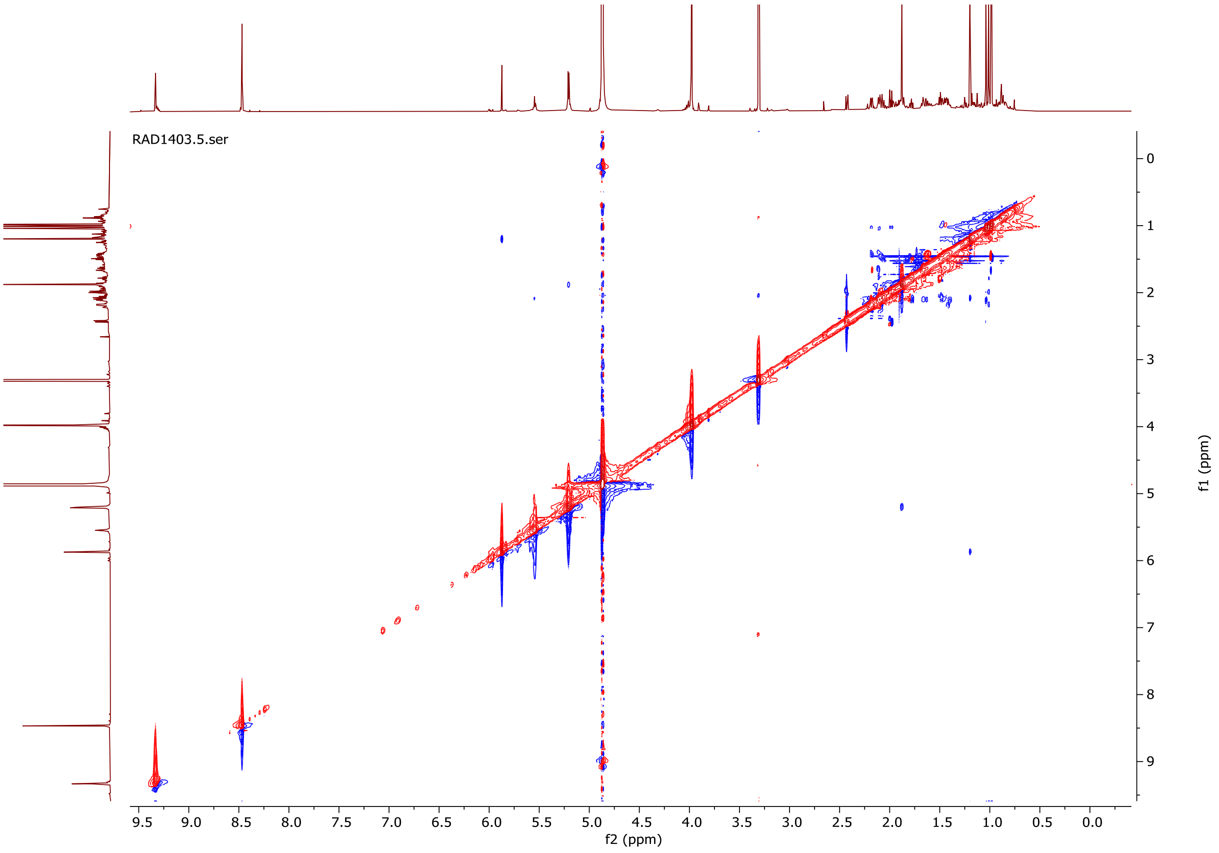

Figure S7 LC-MS data of agelasine Z (**1**)

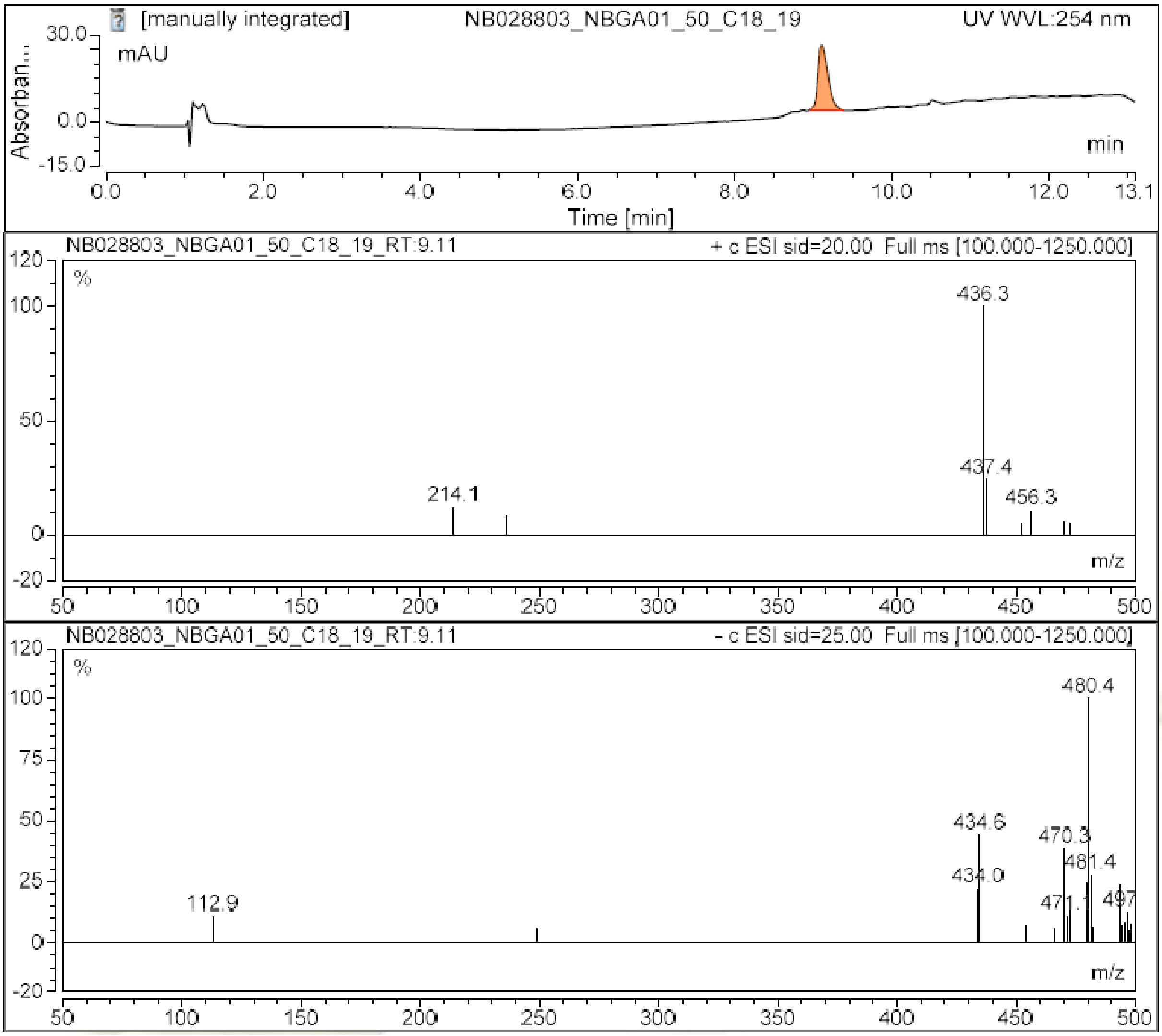

Figure S8 HRESIMS data of agelasine Z (**1**)

Mass Spectrum SmartFormula Report

Analysis Info

Analysis Name	D:\Data\Kanchana\20240108\RAD1403000001.d	Acquisition Date	1/9/2024 11:59:33 AM
Method	DirectInfusion_2018_pos.m	Operator	Demo User
Sample Name	RAD1403	Instrument	maXis II ETD 1823391.22321
Comment			

Acquisition Parameter

Source Type	ESI	Ion Polarity	Positive	Set Nebulizer	0.4 Bar
Focus	Not active	Set Capillary	4500 V	Set Dry Heater	180 °C
Scan Begin	50 m/z	Set End Plate Offset	-500 V	Set Dry Gas	4.0 l/min
Scan End	1300 m/z	Set Collision Cell RF	500.0 Vpp	Set Divert Valve	Waste

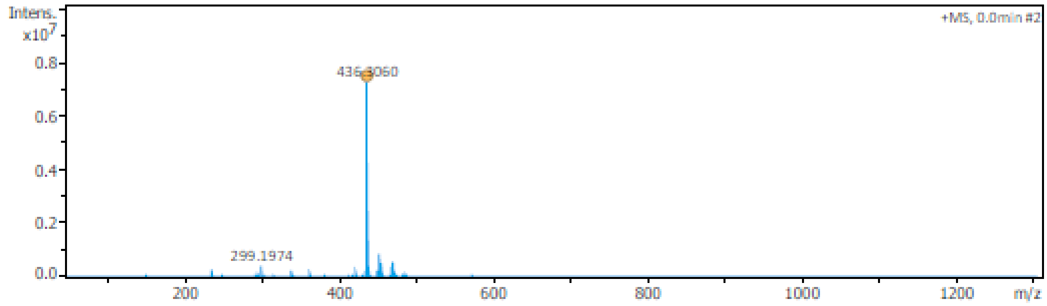

Meas. m/z	#	Ion Formula	m/z	err [ppm]	mSigma	# mSigma	Score	rdb	e¯ Conf	N-Rule
436.3060	1	C26H38N5O	436.3071	2.5	21.2	1	100.00	11.0	even	ok

Table S2 NMR data for agelasine Z (**1**) in MeCN-d_3

Position	δ_H, mult., (*J* in Hz)	HMBC	COSY	ROESY
1	5.79, s	9	-	20
2	-	-	-	-
3a	2.34, d (15.4)	2	3b	-
3b	1.95, m	2	3a	-
4	-	-	-	-
5	-	-	-	-
6a	2.04, m	5, 8, 10, 20	7a, 7b	-
6b	1.27, m		7a, 7b	-
7a	1.61, m	5, 8, 9	6a, 6b, 8	-
7b			6a, 6b, 8	-
8	1.44, m	9, 10	7a, 7b, 9	9, 19
9	2.15, dd (13.0, 4.0)	10, 11	8, 11a, 11b	-
10	-	-	-	-
11a	1.44, m	8, 10, 13	9, 12a, 12b	-
11b	1.39, m		9, 12a, 12b	-
12a	2.01, m	14, 16	11a, 11b	-
12b	1.74, td (13.0, 3.5)		11a, 11b	-
13	-	-	-	-
14	5.45, t (7.0)	12, 15, 16	15a, 15b	-
15a	5.08, d (7.0)	13, 14, 5', 8',	14	-
15b				-
16	1.80, s	-	-	-
17	0.95, d (7.0)	7, 9	8	9, 20
18	0.98, s	3, 4, 5, 19	-	-
19	1.00, s	3, 4, 5, 18	-	-
20	1.16, s	5, 6, 10, 18, 19	-	-
2'	8.47, s	4', 6'	-	1, 18
4'	-	-	-	-
5'	-	-	-	-
6'	-	-	-	-
8'	8.90, s	4', 6'	-	-
NH_2	n.d.	-	-	-
9-*N*CH_3	3.90, s	4', 8'	-	-

n.d.: not detected

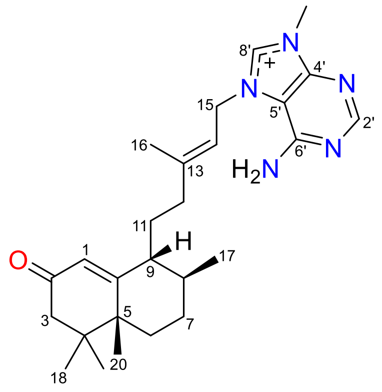

Figure S9 ^{1}H NMR (800 MHz) spectrum of agelasine Z (1) in MeCN-d_3

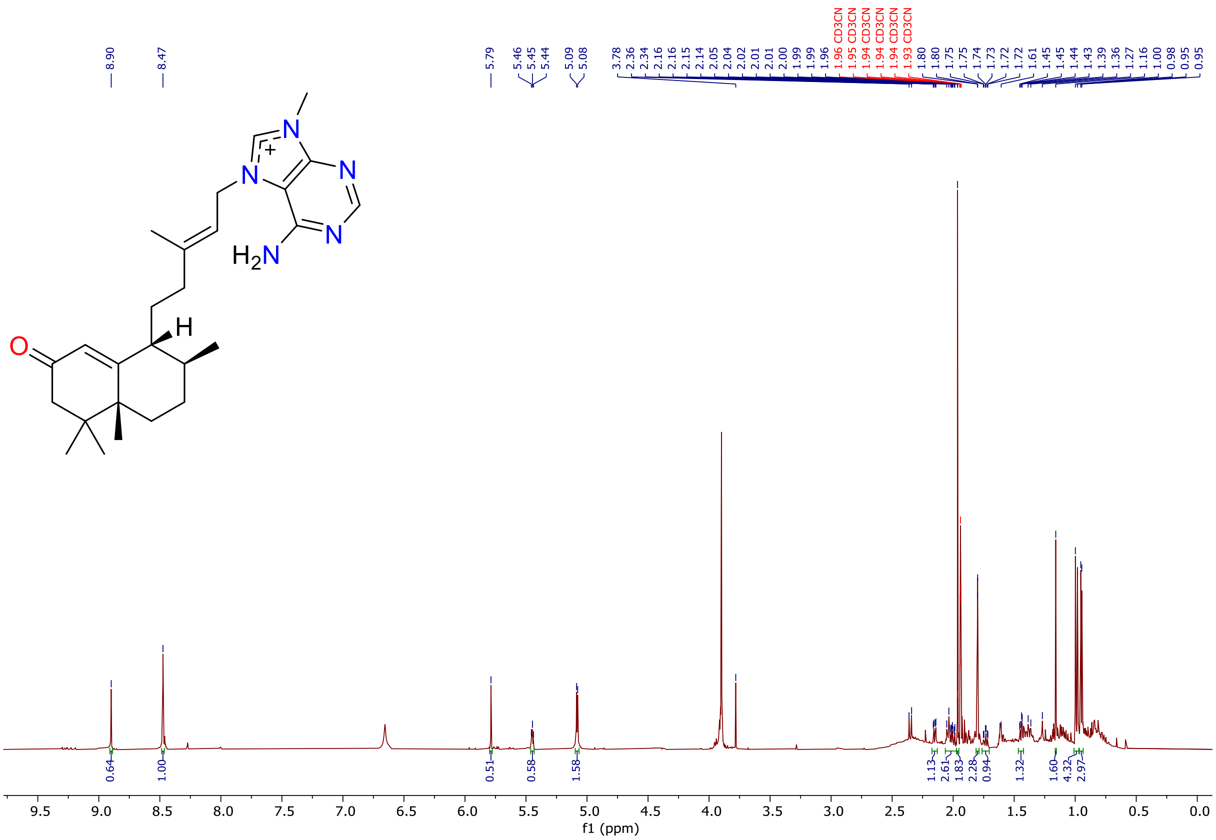

Figure S10 COSY spectrum of agelasine Z (1) in MeCN-d_3

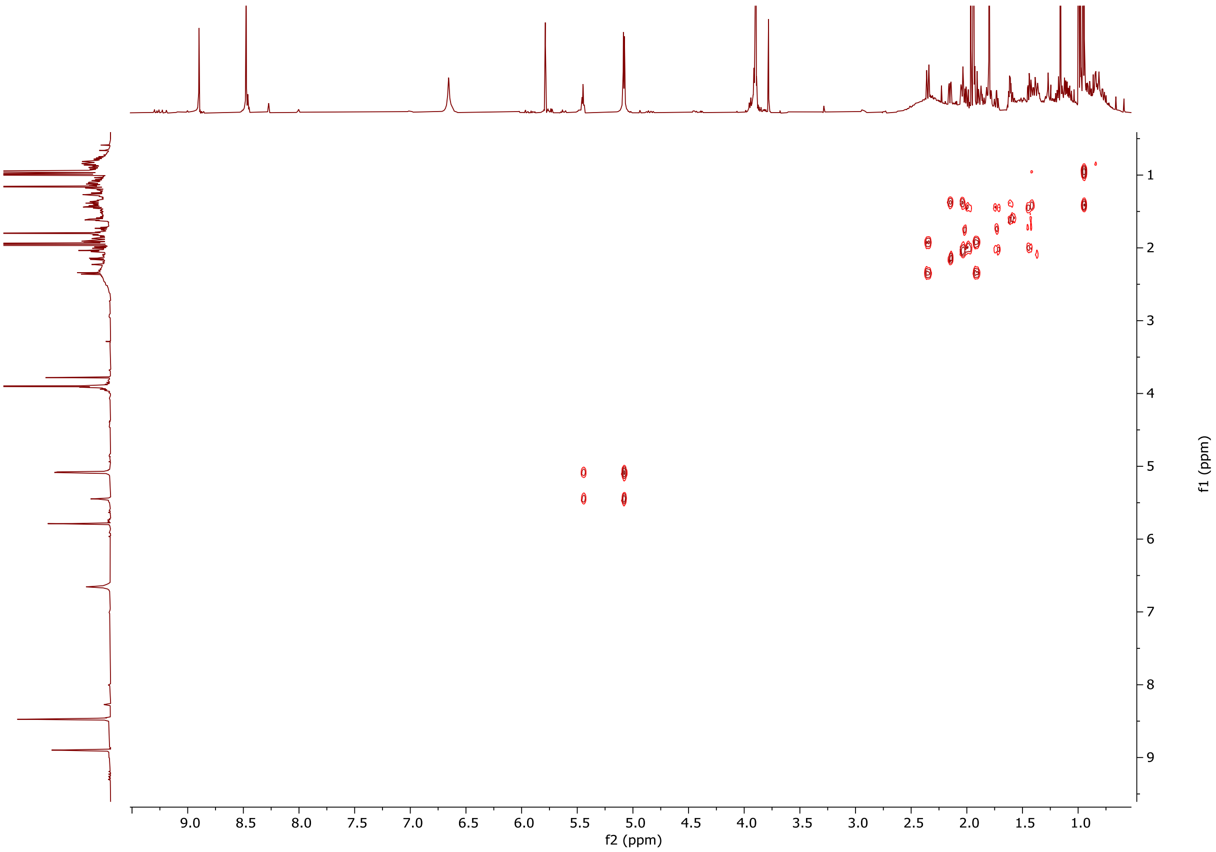

Figure S11 ROESY spectrum of agelasine Z (**1**) in MeCN-*d*3

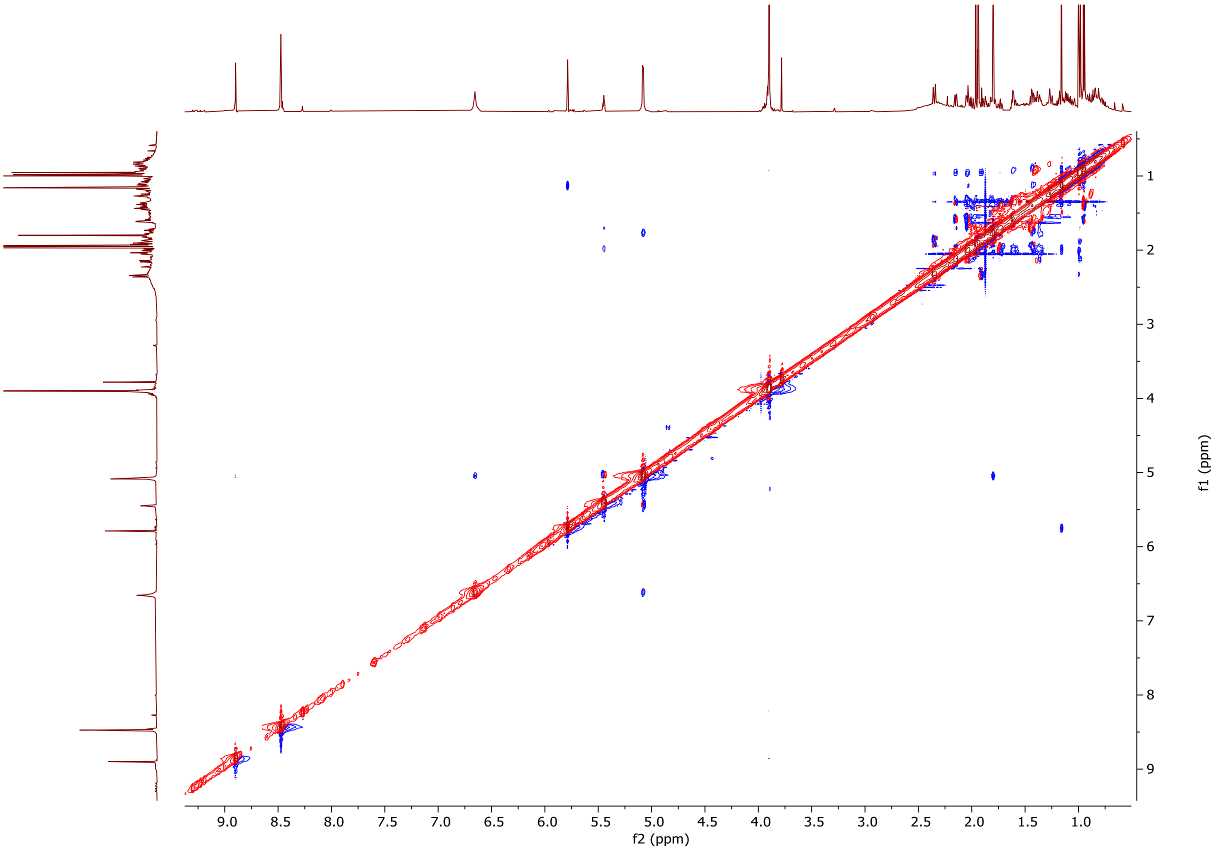

Figure S12 ^{1}H NMR (800 MHz) spectrum of agelasine B (**2**) in MeOH-*d*4

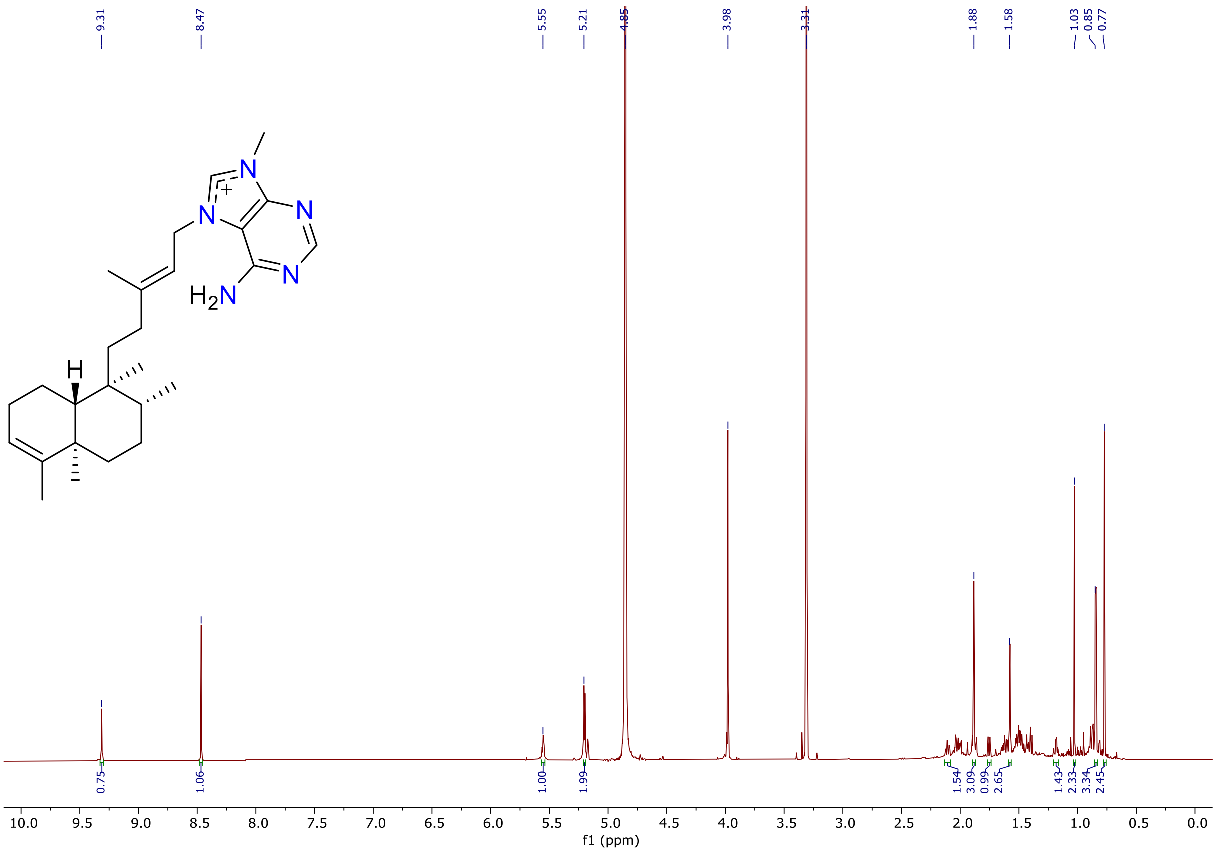

Figure S13 ^{13}C NMR (200 MHz) spectrum of agelasine B (**2**) in MeOH-d_4

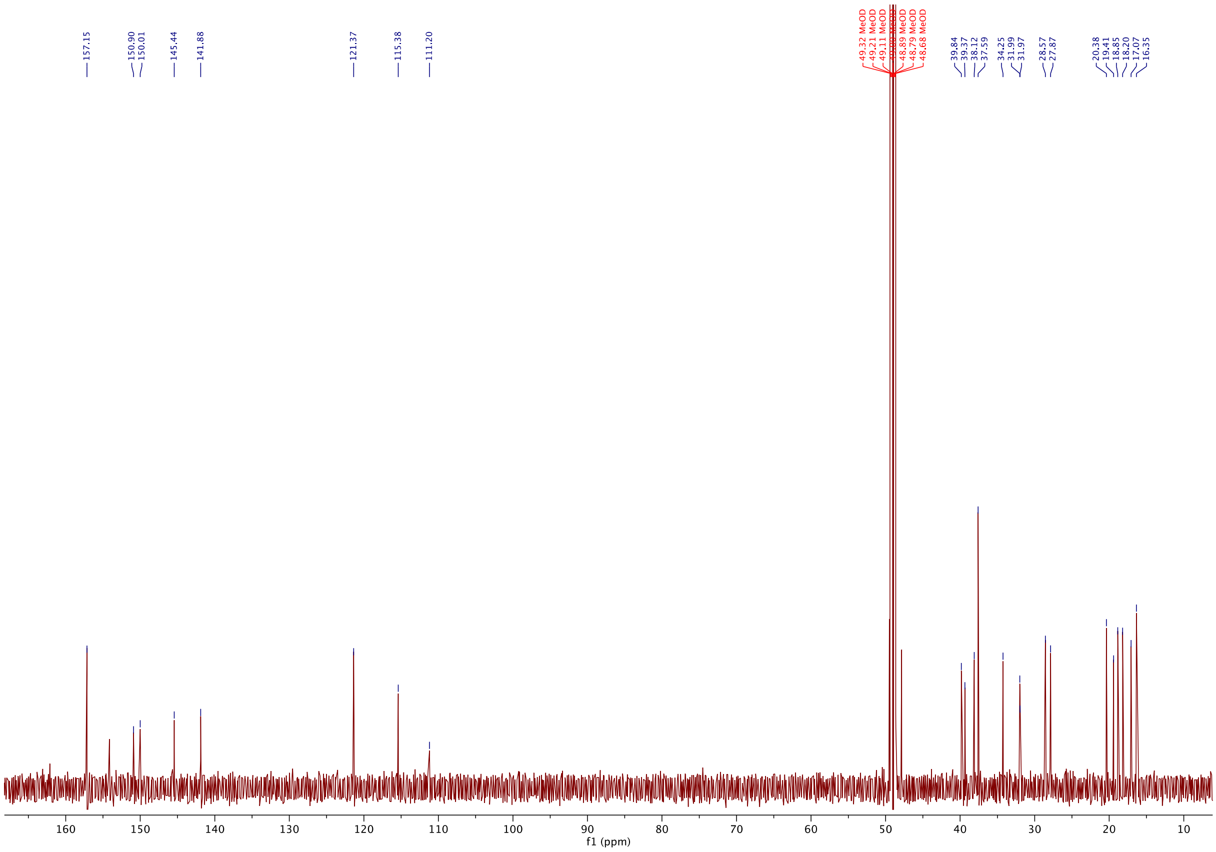

Figure S14 ^{1}H NMR (800 MHz) spectrum of oxoagelasine B (**3**) in MeOH-d_4

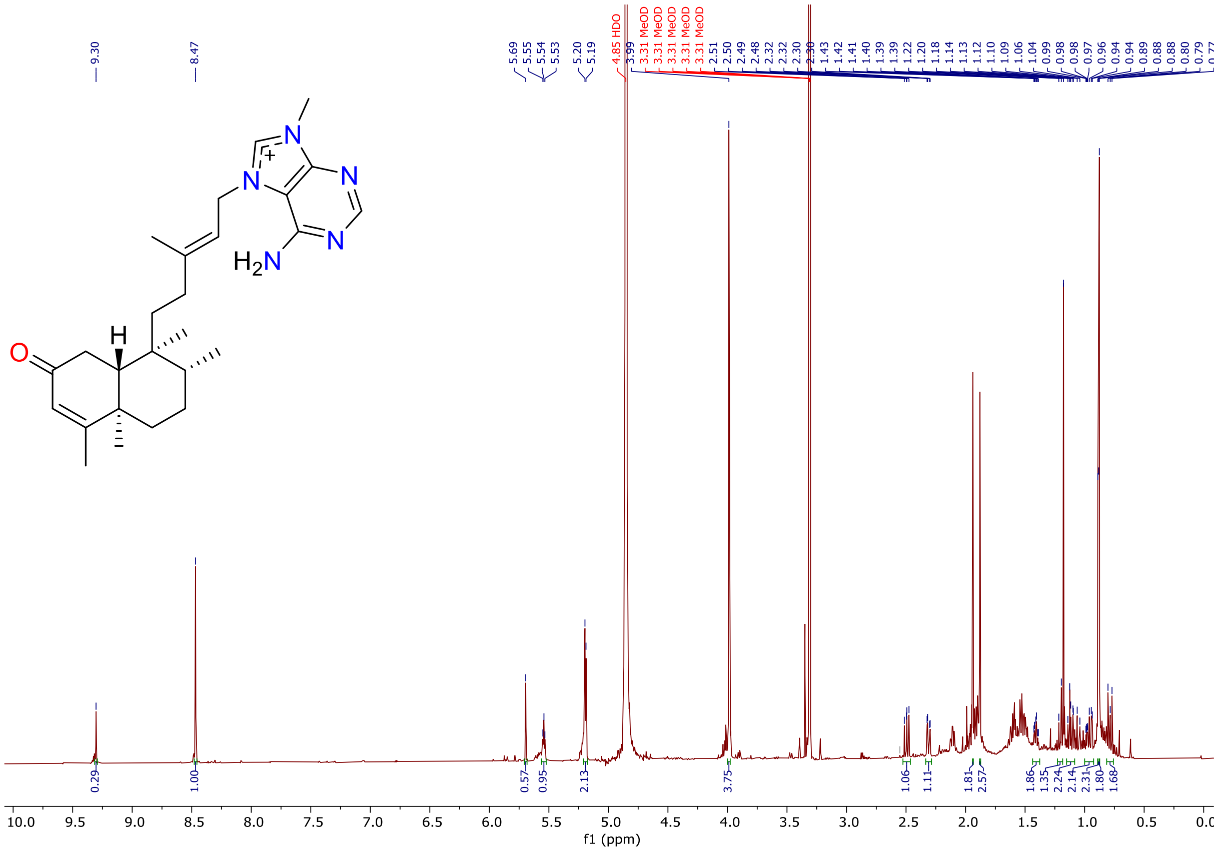

Figure S15 ^{13}C NMR (200 MHz) spectrum of oxoagelasine B (**3**) in MeOH-d_4

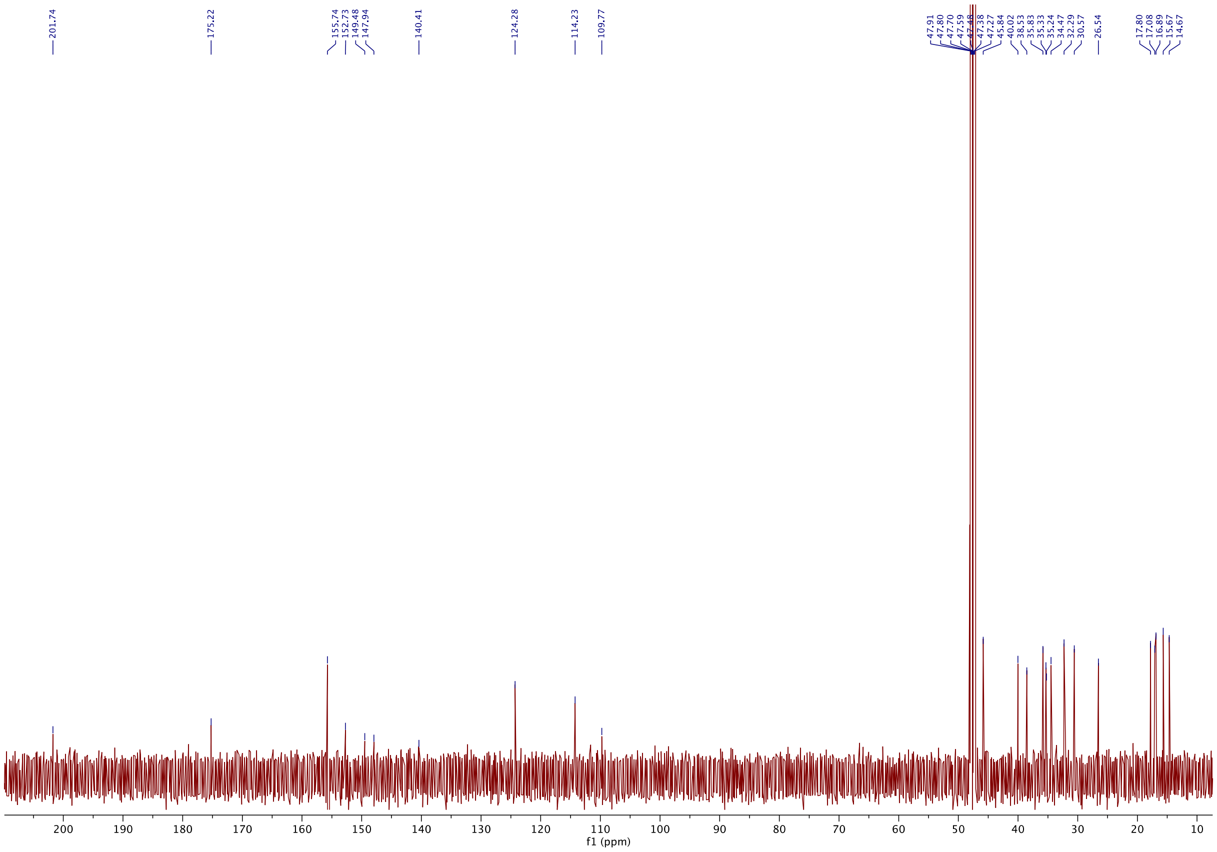

Figure S16 ^{1}H NMR (800 MHz) spectrum of agelasine D (**6**) in MeOH-d_4

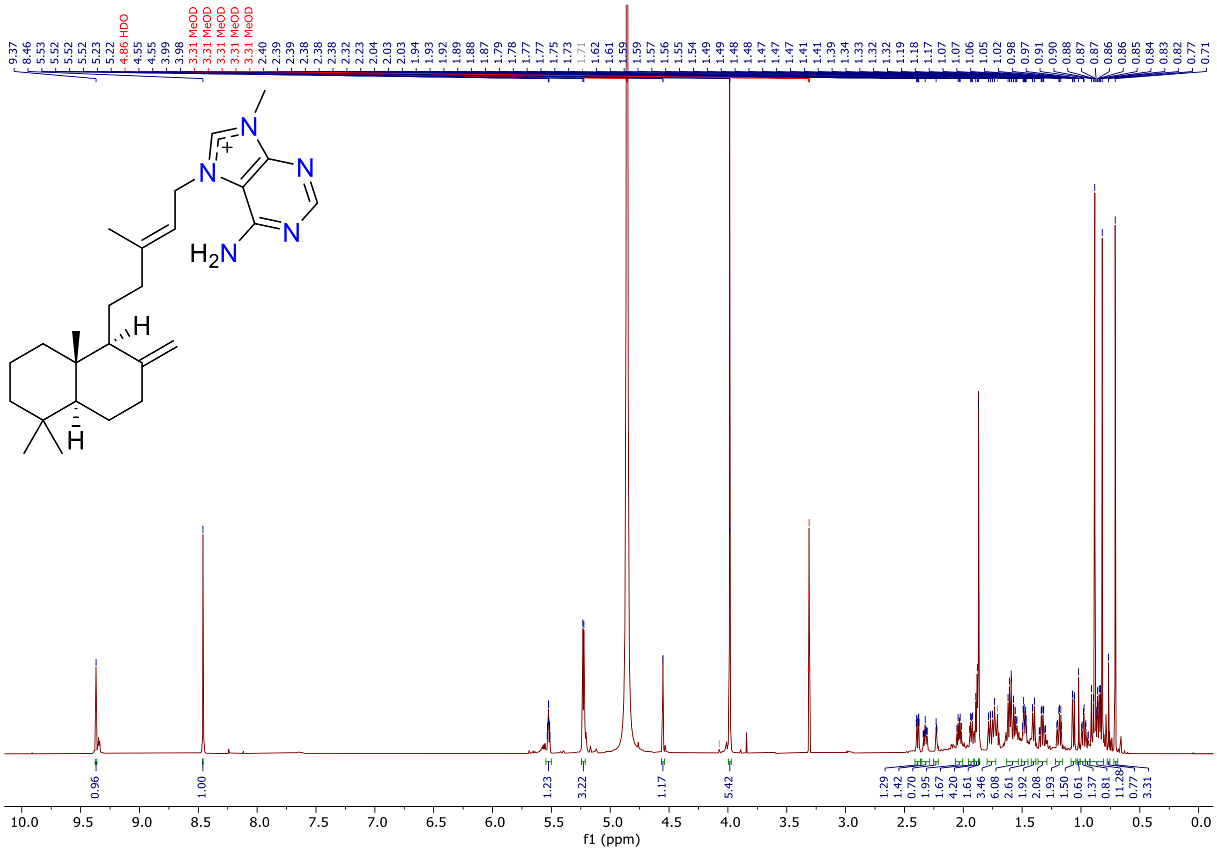

Figure S17 ^{13}C NMR (200 MHz) spectrum of agelasine D (**6**) in MeOH-d_4

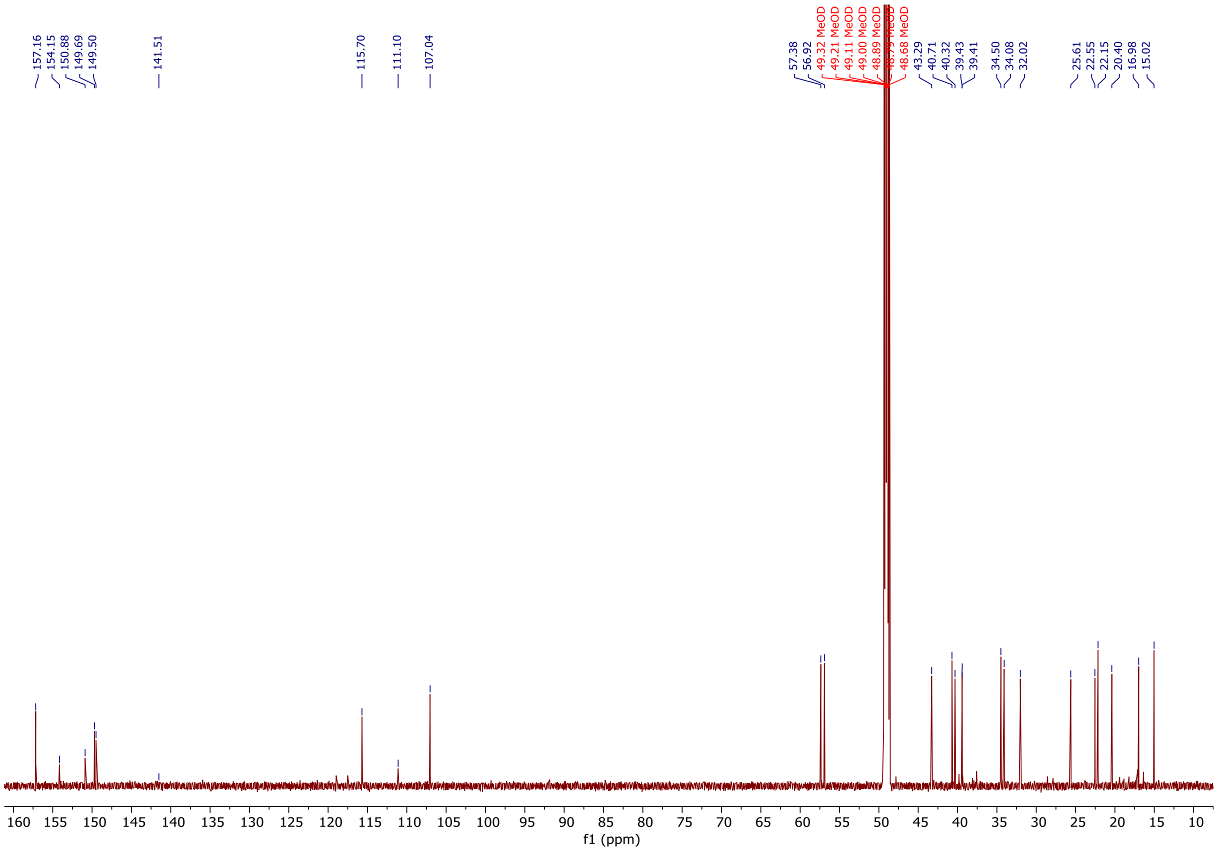

Figure S18 ECD spectra of compounds **1-3** and **6**

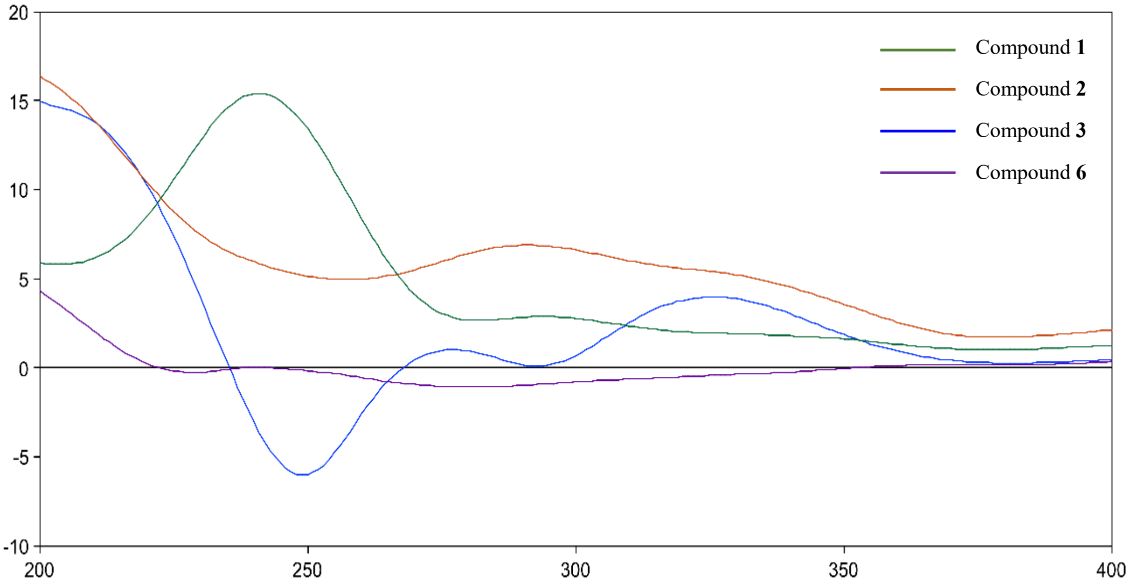

Figure S19 ^{1}H NMR (800 MHz) spectrum of mukanadin C (**4**) in MeOH-d_4

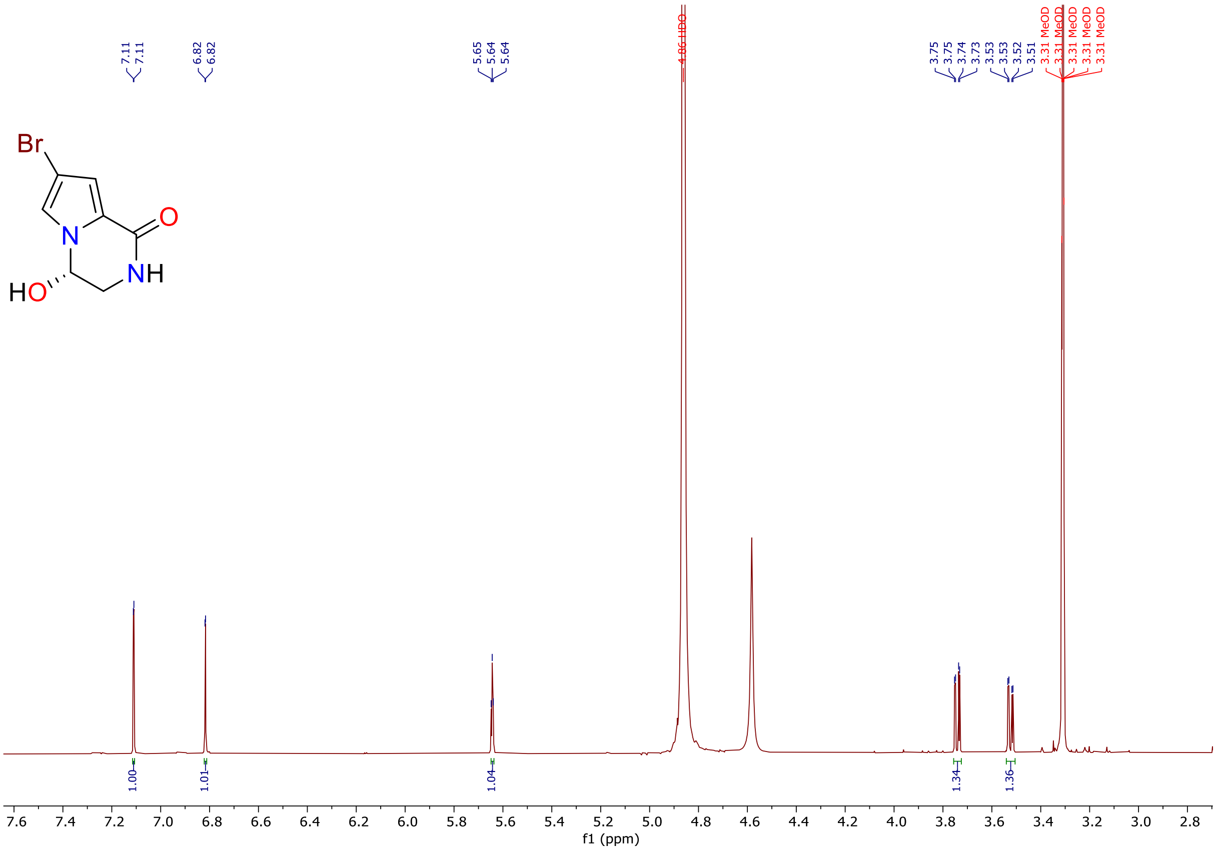

Figure S20 ^{13}C NMR (200 MHz) spectrum of mukanadin C (**4**) in MeOH-d_4

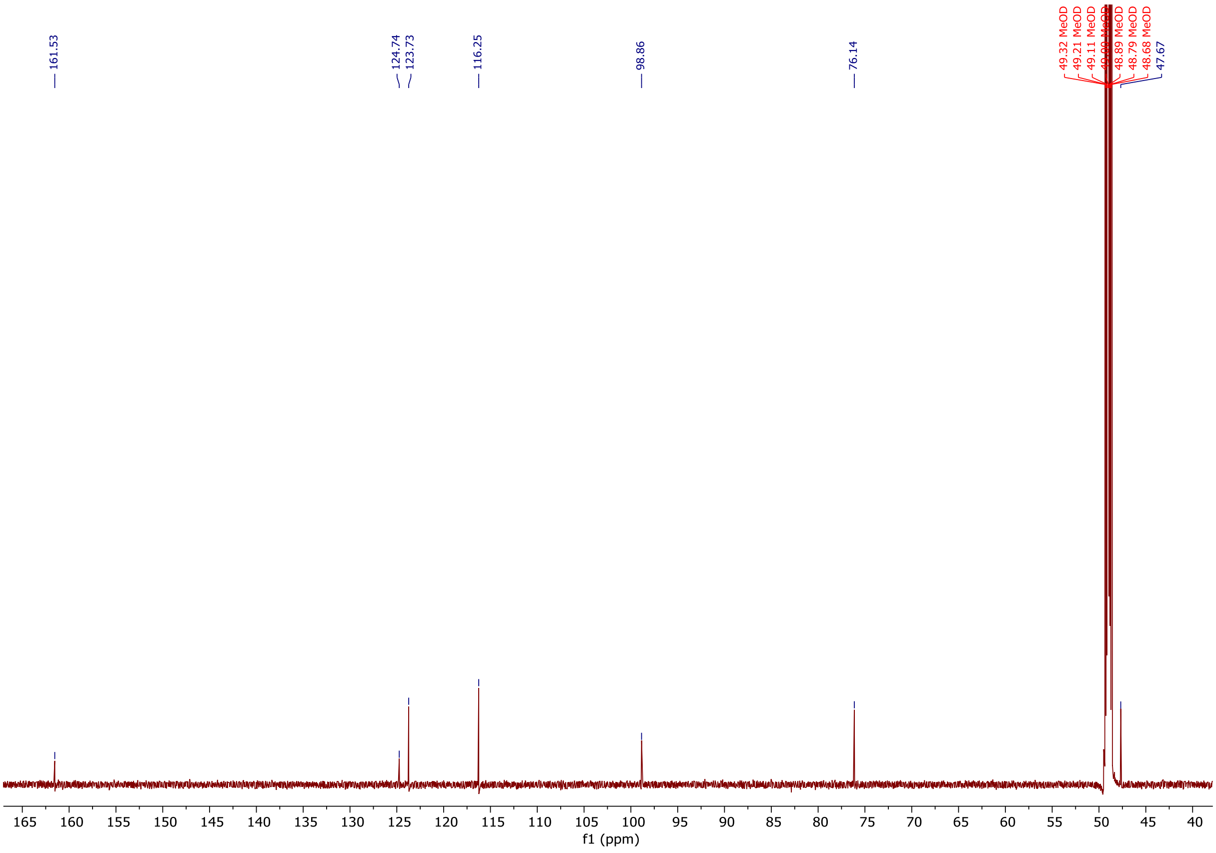

Figure S21 ^{1}H NMR (800 MHz) spectrum of 4-bromopyrrole-2-carboxylic acid (**5**) in MeOH-d_4

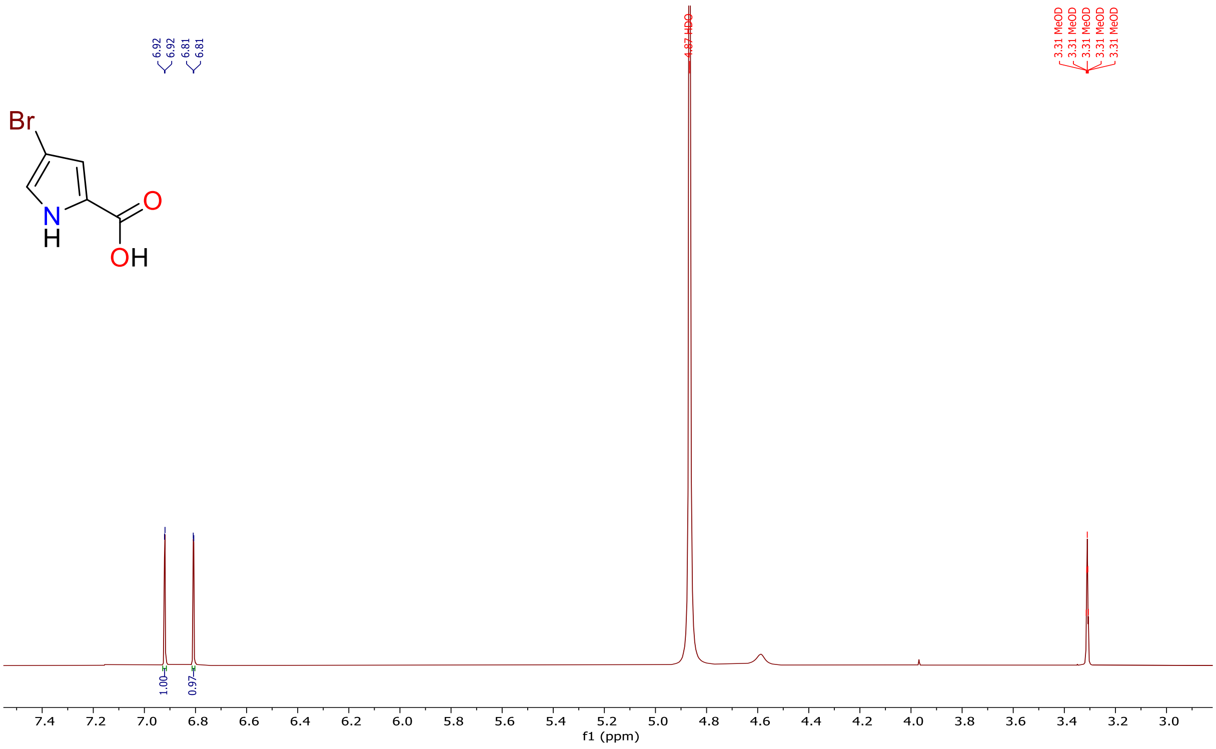

Figure S22 ^{13}C NMR (200 MHz) spectrum of 4-bromopyrrole-2-carboxylic acid (**5**) in MeOH-d_4

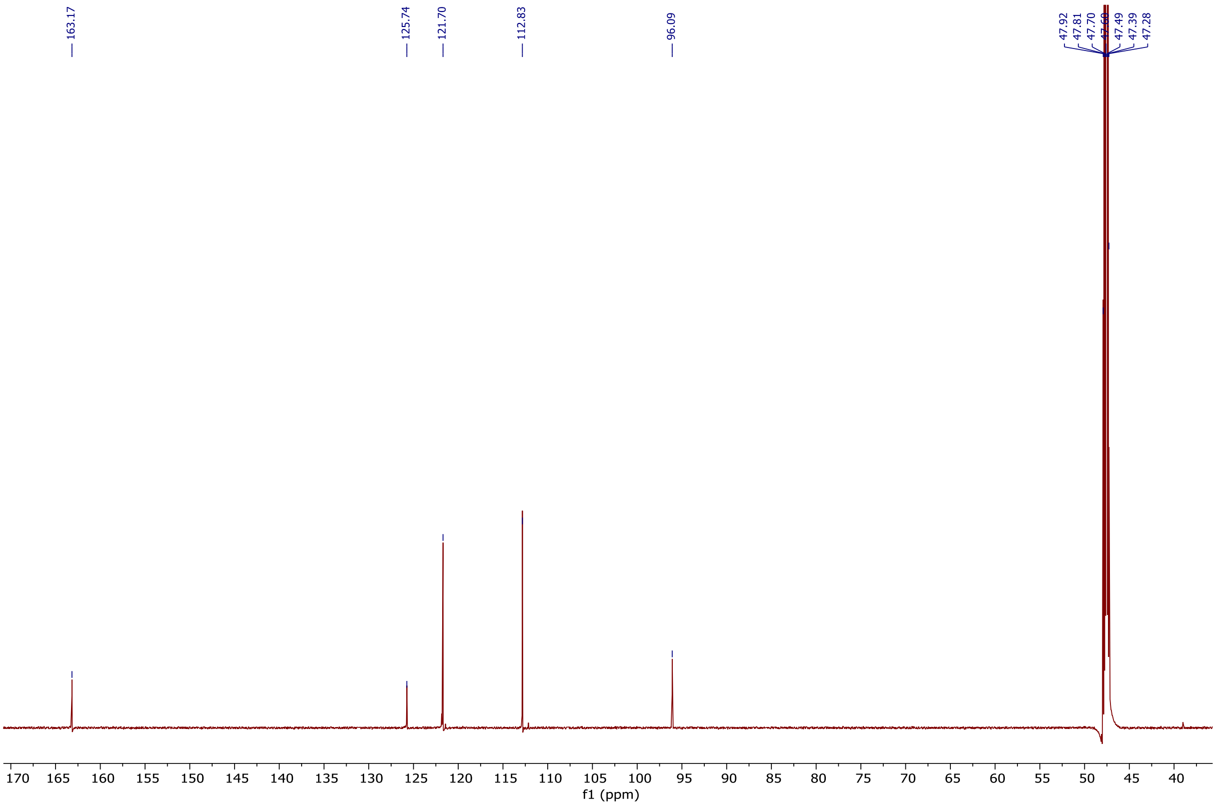

Figure S23 Stacked ^{1}H NMR spectra of agelasine D (**6**) in MeOH-d_4 at 25 °C recorded at 0, 1, 4, 24, 48, 72, 96, 120 and 144 h

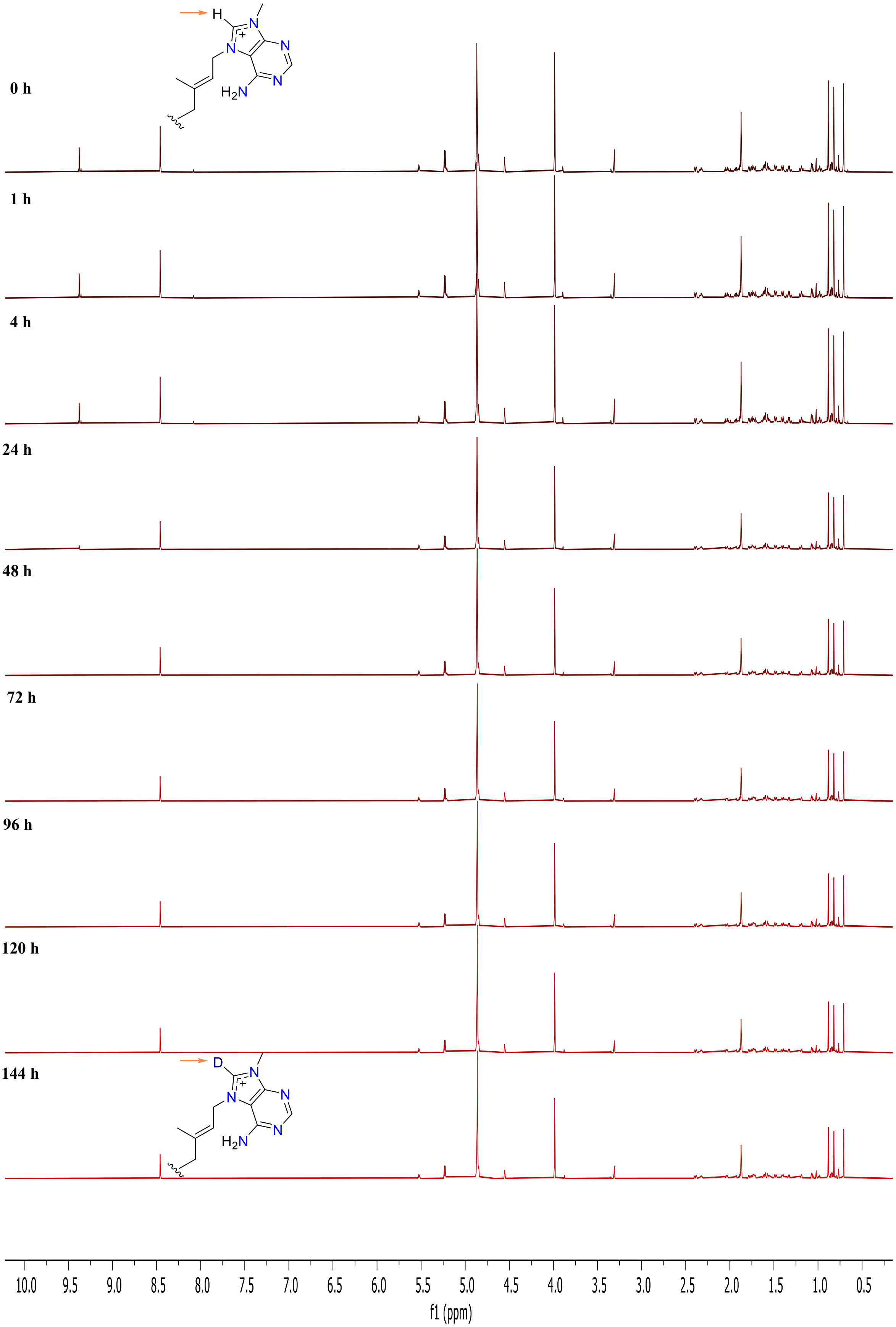

Figure S24 Stacked ^{1}H NMR spectra of agelasine B (**2**) in MeOH-d_4 at 25 °C recorded at 0, 48 and 120 h

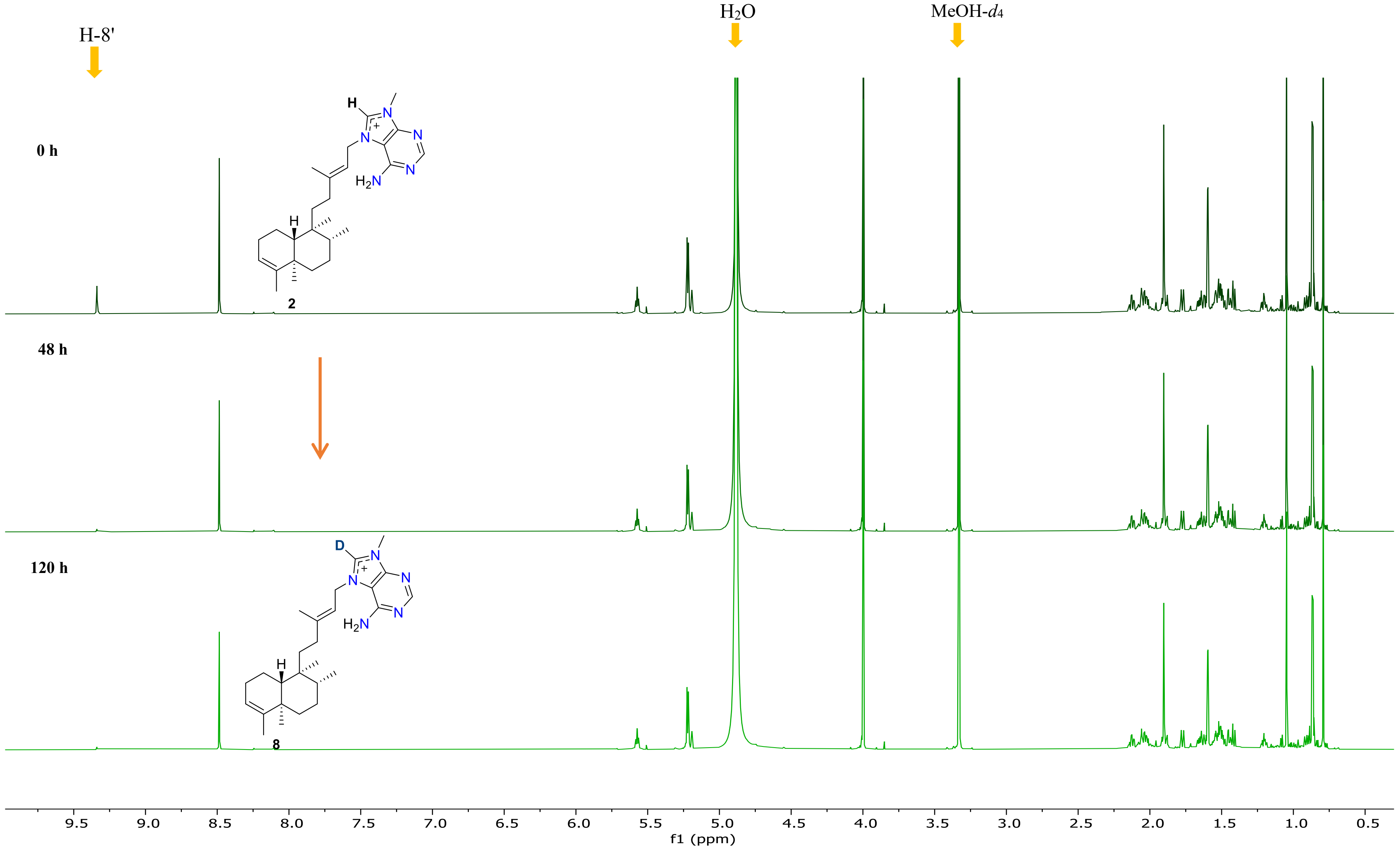

Figure S25 Stacked ^{1}H NMR spectra of oxoagelasine B (**3**) in MeOH-d_4 at 25 °C recorded at 0, 48 and 120 h

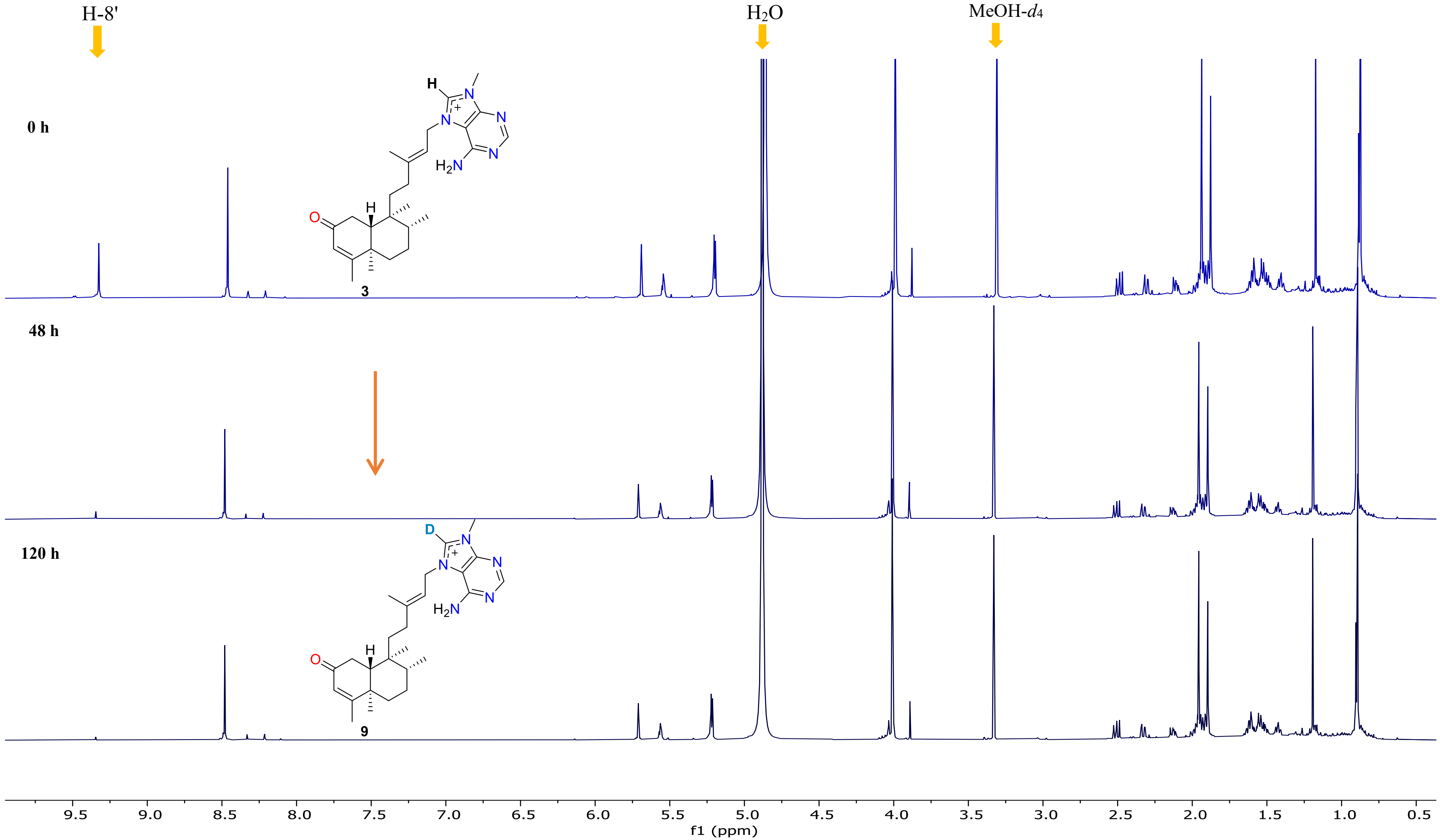

Table S3 NMR data for deuterated agelasine D (**7**) in MeOH-d_4

Position	δ_C, type	δ_H, mult., (J in Hz)	HMBC	COSY	ROESY
1a	40.3, CH_2	1.78, m	2, 3, 5, 10	2	
1b		0.98, td (12.8, 3.5)			
2a	20.4, CH_2	1.74, m	1, 4, 10	1, 3	
2b		1.57, m			
3a	43.3, CH_2	1.40, m	1, 5, 18, 19	2	
3b		1.19, td (13.5, 3.7)			
4	34.5, C	-	-	-	-
5	57.4, CH	1.06, dd (12.6, 2.8)	3, 4, 6, 7, 10	6	
6a	25.6, CH_2	1.75, m	4, 5, 8, 10	5, 7	
6b		1.33, m			
7a	39.4, CH_2	2.39, m	7, 9	6	
7b		1.93, m			
8	149.7, C	-	-	-	
9	56.9, CH	1.60, m	8, 10, 11	11	
10	40.7, C	-	-	-	
11a	22.5, CH_2	1.71, m	9, 13	12	
11b		1.55, m			
12a	39.4, CH_2	2.33, m	13	11	
12b		2.04, m			
13	149.5, C	-	-	-	-
14	115.7, CH	5.52, t (7.2)	12, 16	15	
15a	48.6, CH_2	5.23, d (7.2)	14, 5', 8'	14	
15b					
16	17.0, CH_3	1.87, s			
17a	107.0, CH_2	4.88, m	8, 9	-	
17b		4.59, brs	8, 9	-	
18	34.1, CH_3	0.87, s	3, 4, 5	-	
19	22.1, CH_3	0.82, s	3, 4, 5	-	
20	15.0, CH_3	0.71, s	10	-	
2'	157.2, CH	8.47, s	4', 6'	-	-
4'	150.9, C	-	-	-	-
5'	111.1, C	-	-	-	-
6'	145.4, C	-	-	-	-
8'	142.0, CD	-	-	-	-
NH_2		n.d.			
9-NCH$_3$	32.0, CH_3	3.98, s	4', 5'	-	-

n.d.: not detected

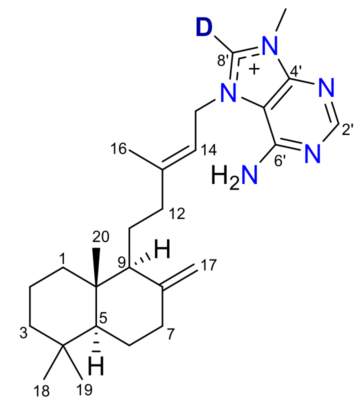

Figure S26 ^{1}H NMR (800 MHz) spectrum of deuterated agelasine D (**7**) in MeOH-*d*4

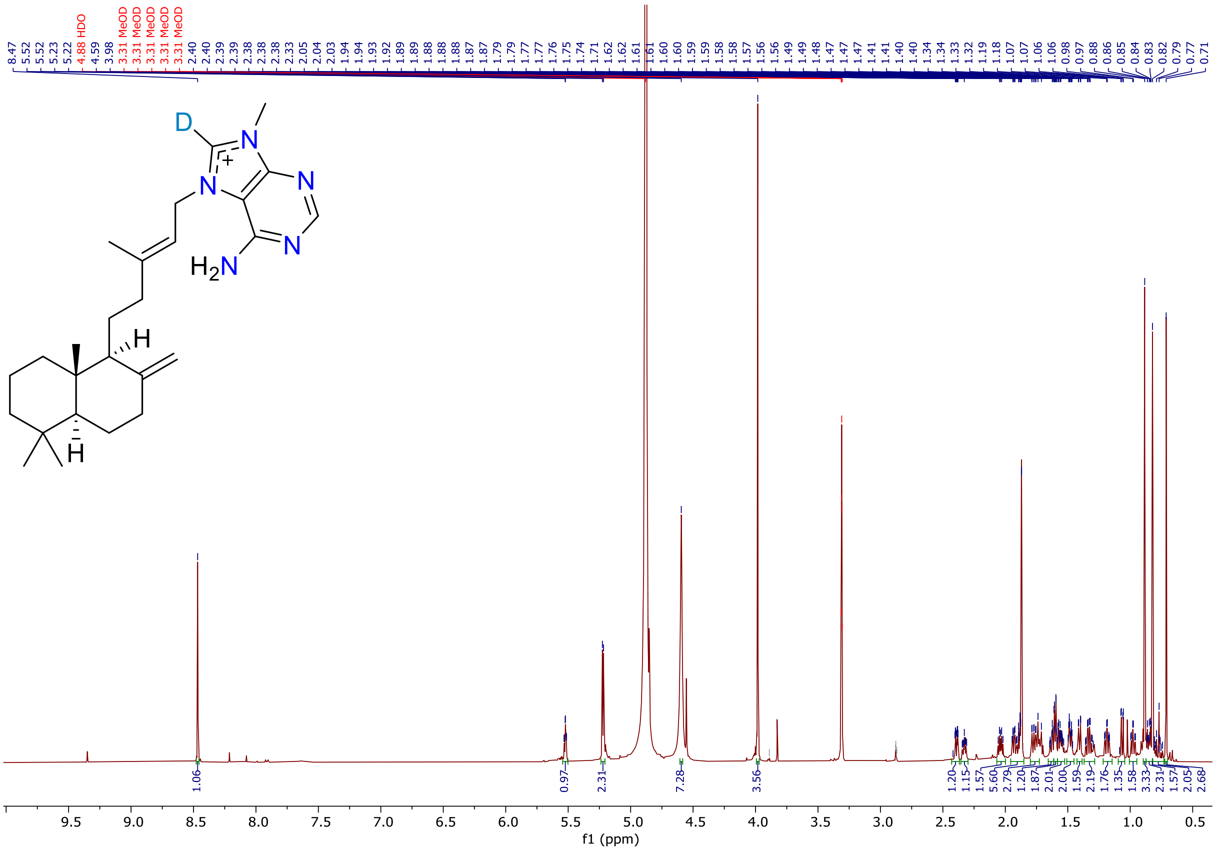

Figure S27 ^{13}C NMR (200 MHz) spectrum of deuterated agelasine D (**7**) in MeOH-*d*4

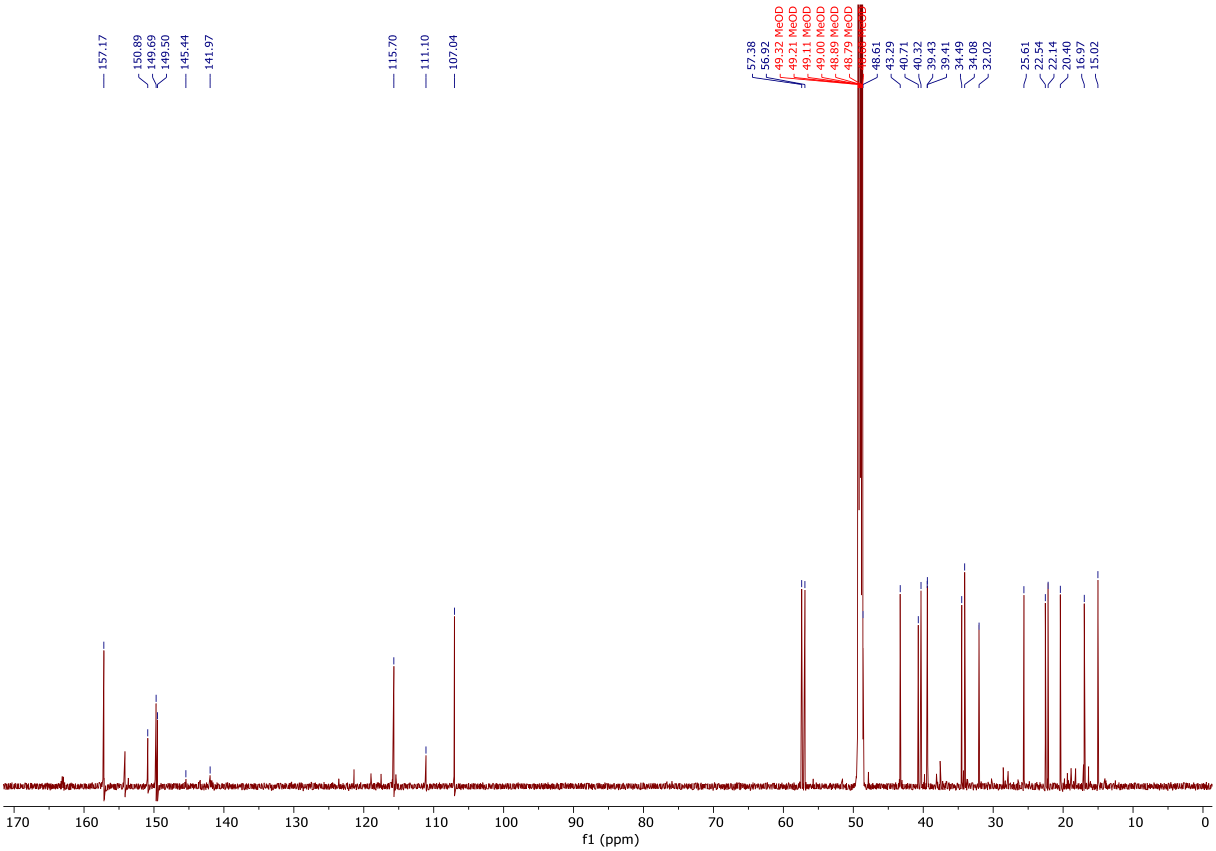

Figure S28 COSY spectrum of deuterated agelasine D (**7**) in MeOH-d_4

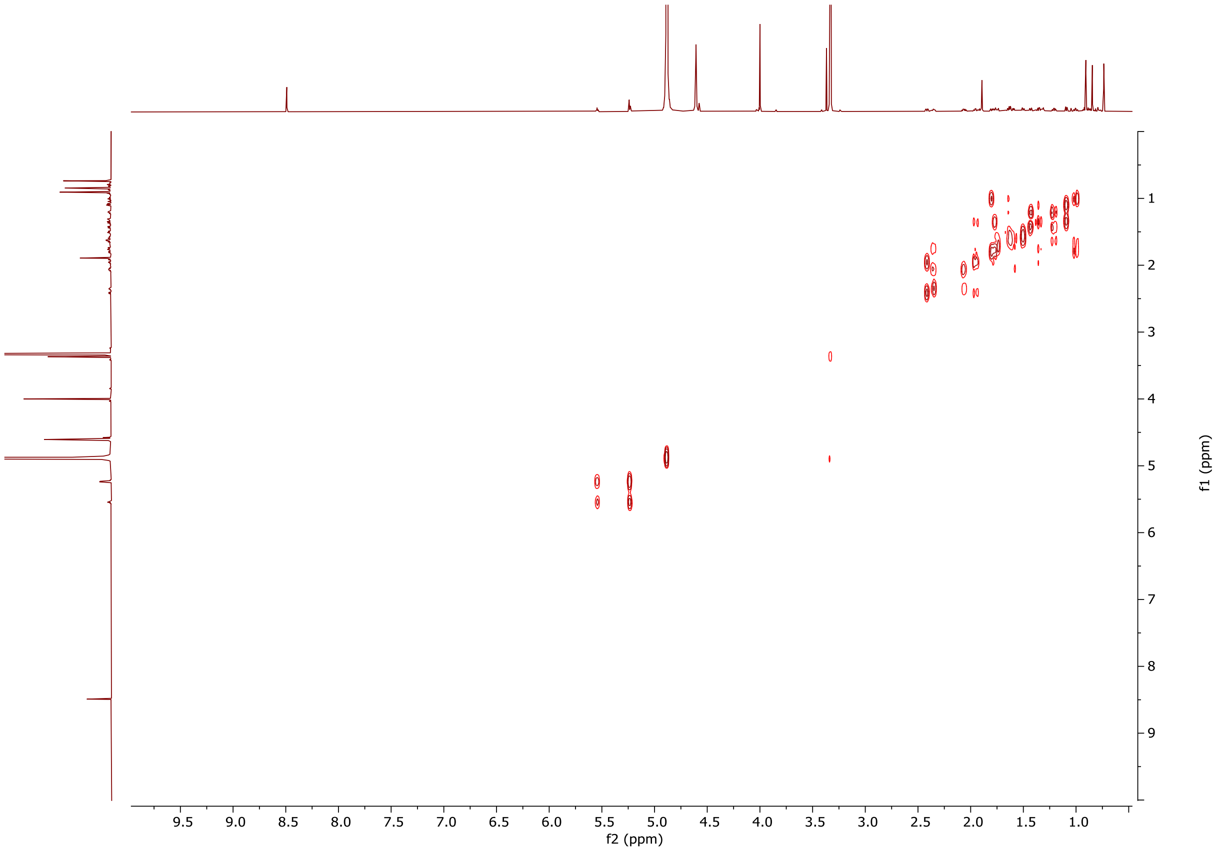

Figure S29 HSQC spectrum of deuterated agelasine D (**7**) in MeOH-d_4

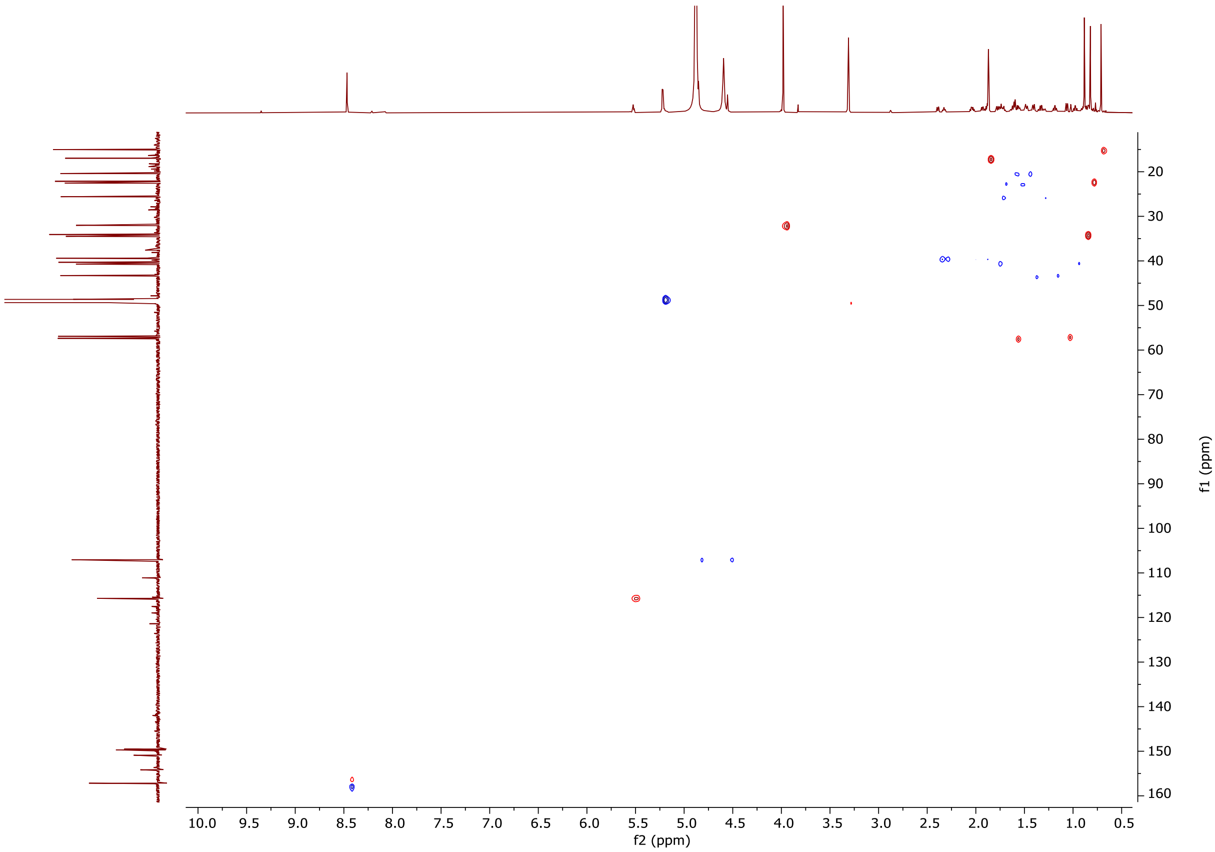

Figure S30 HMBC spectrum of deuterated agelasine D (**7**) in MeOH-*d*4

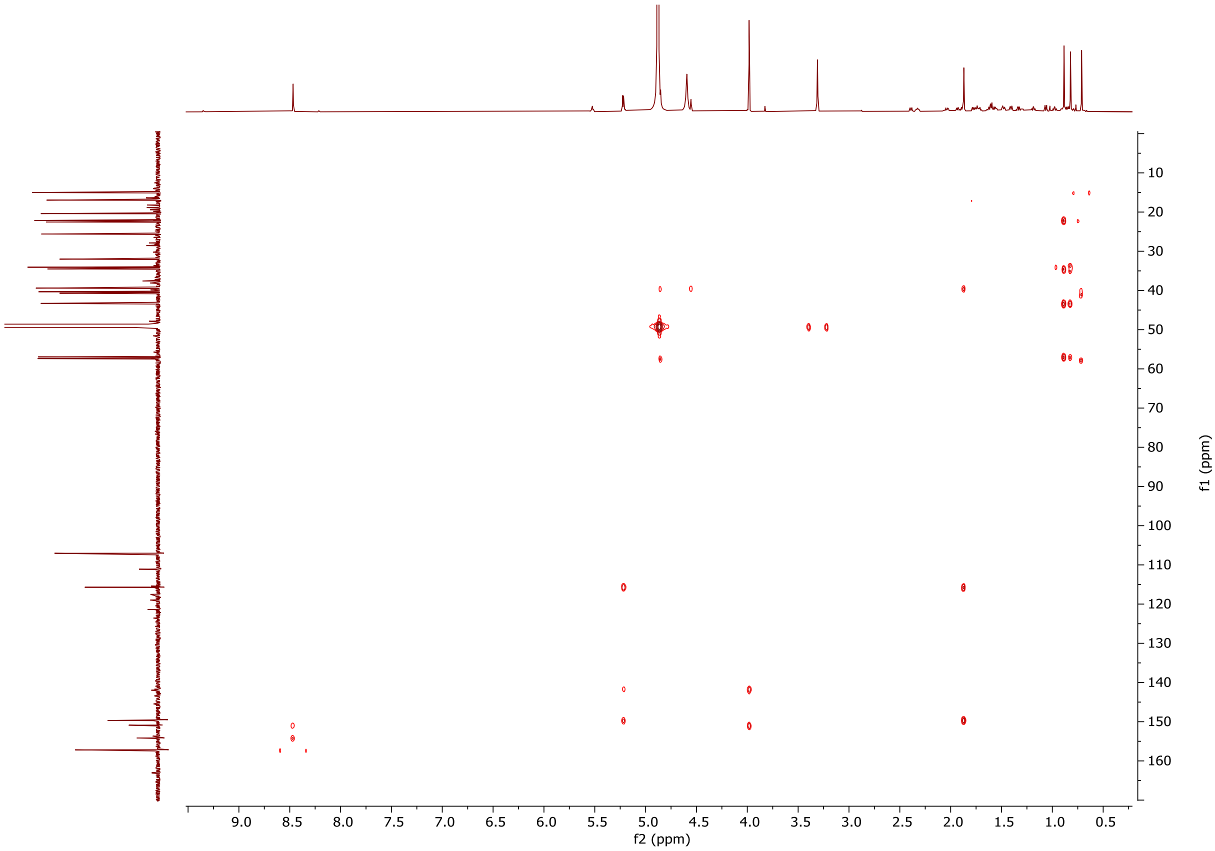

Figure S31 ROESY spectrum of deuterated agelasine D (**7**) in MeOH-*d*4

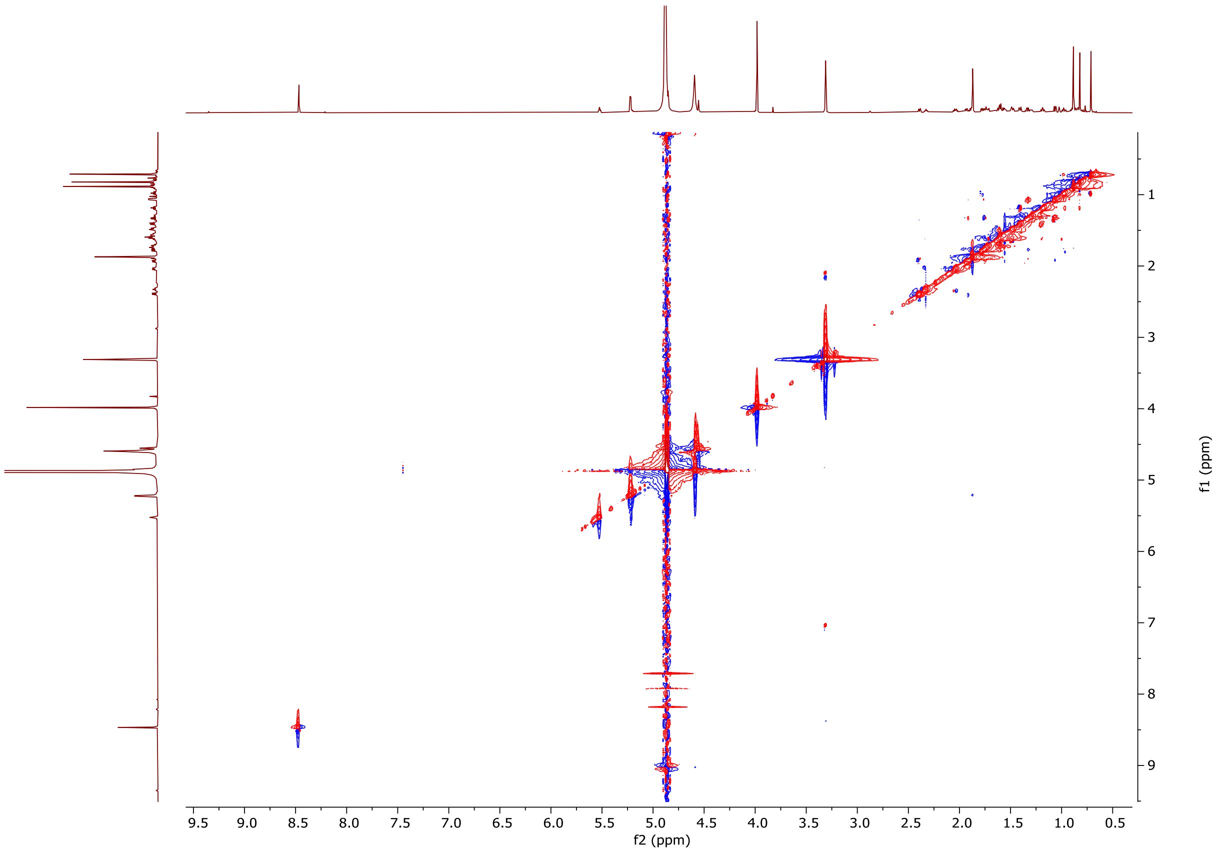

Figure S32 LC-MS data of deuterated agelasine D (**7**)

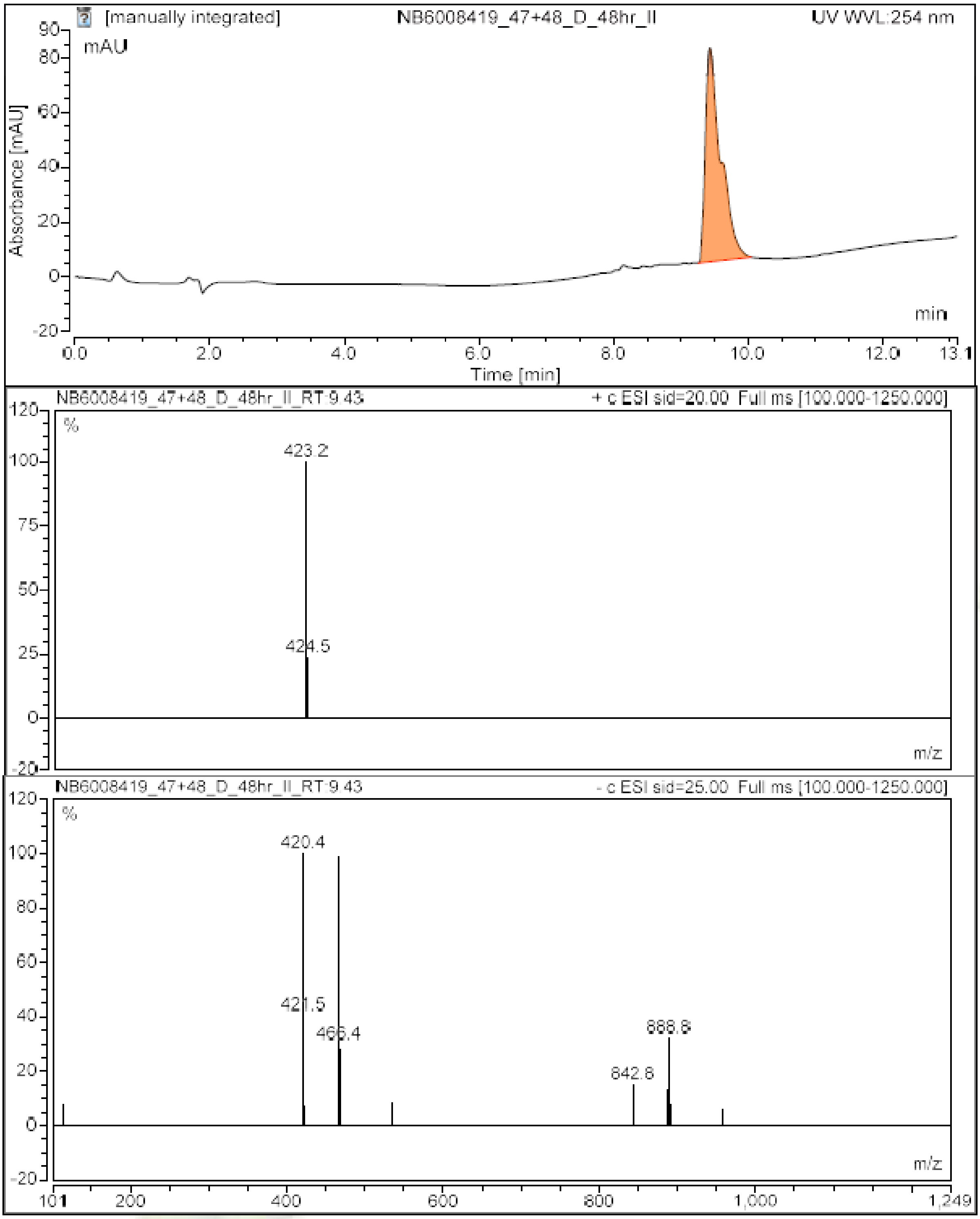

Figure S33 ^{1}H NMR (800 MHz) spectrum of deuterated agelasine B (**8**) in MeOH-d_4

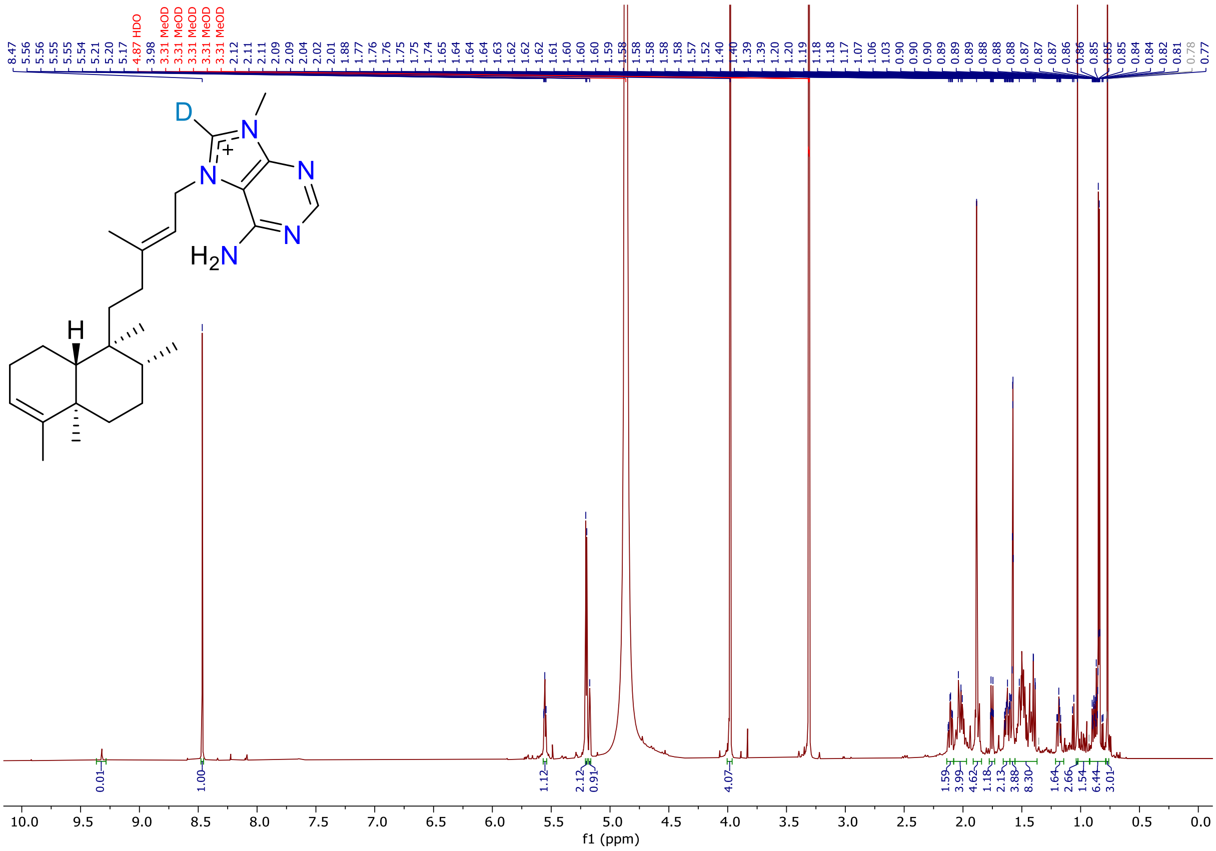

Figure S34 ^{13}C NMR (200 MHz) spectrum of deuterated agelasine B (**8**) in MeOH-d_4

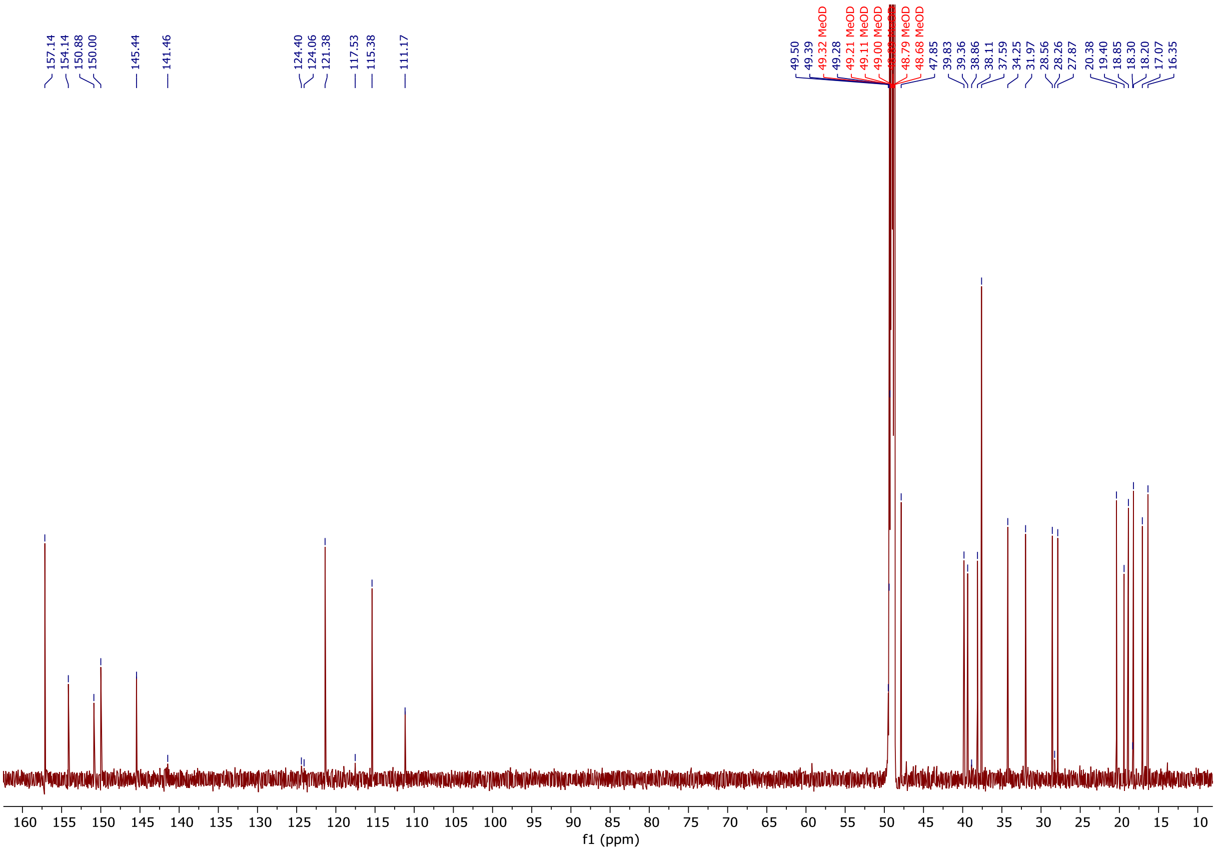

Figure S35 LC-MS data of deuterated agelasine B (**8**)

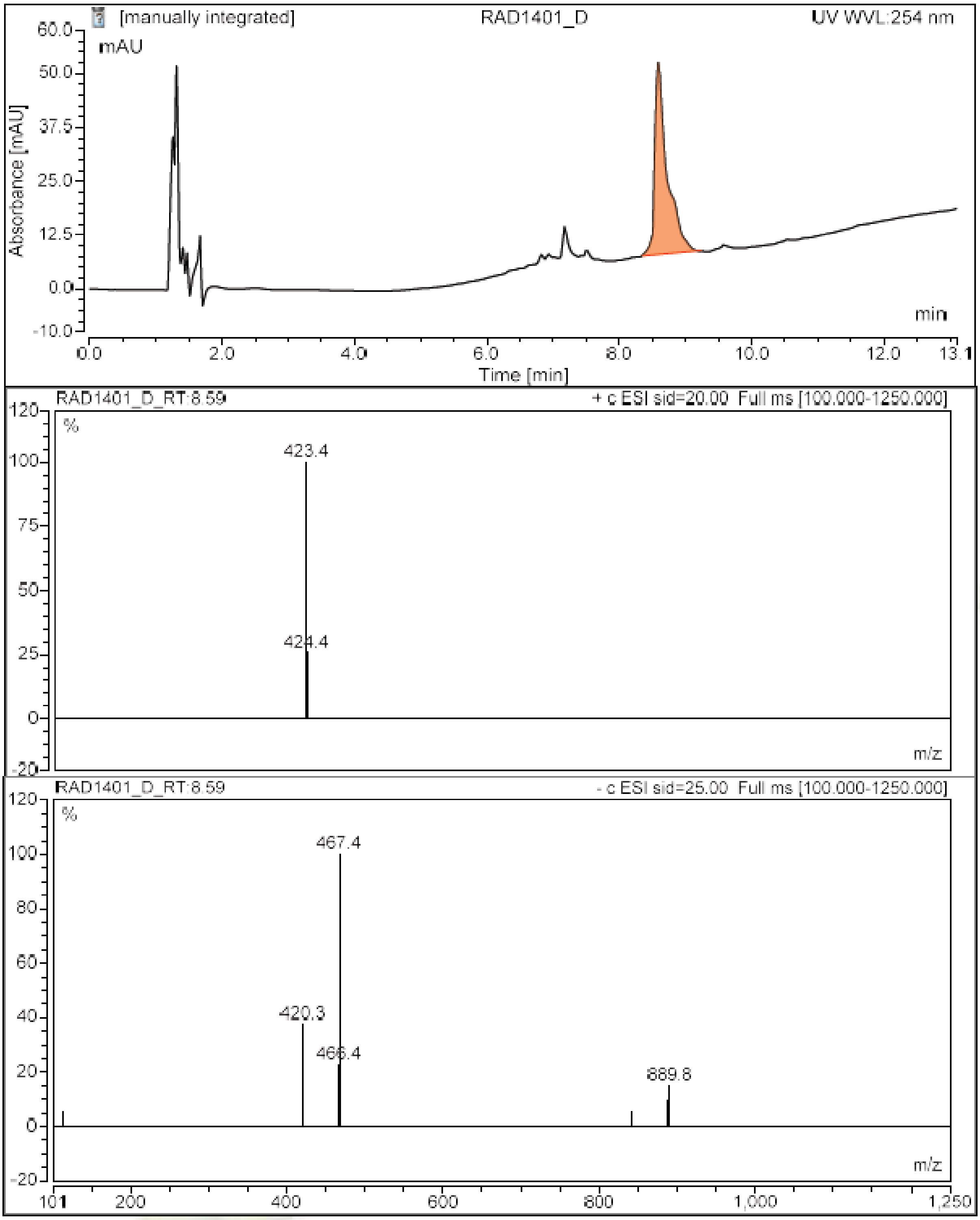

Figure S36 ^{1}H NMR (800 MHz) spectrum of deuterated oxoagelasine B (**9**) in MeOH-d_4

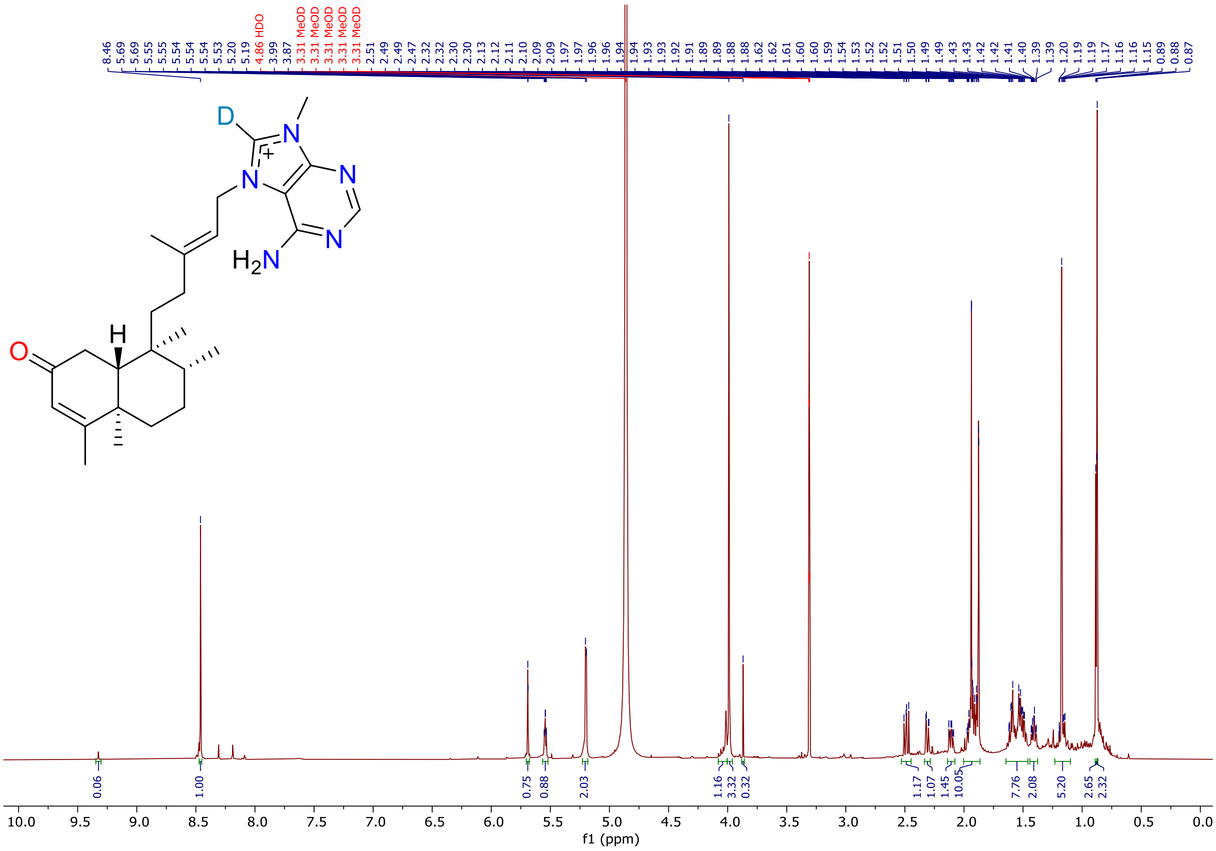

Figure S37 ^{13}C NMR (200 MHz) spectrum of deuterated oxoagelasine B (**9**) in MeOH-d_4

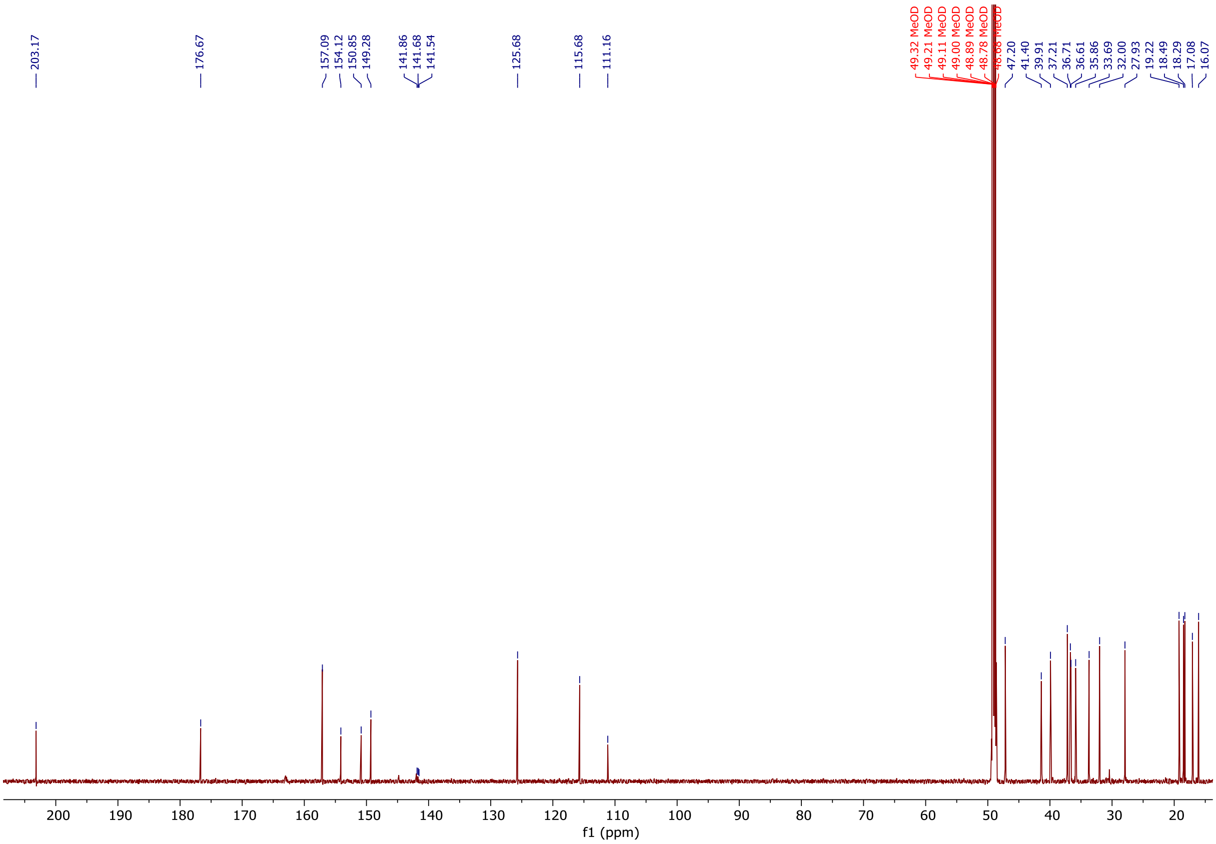

Figure S38 LC-MS data of deuterated oxoagelasine B (**9**)

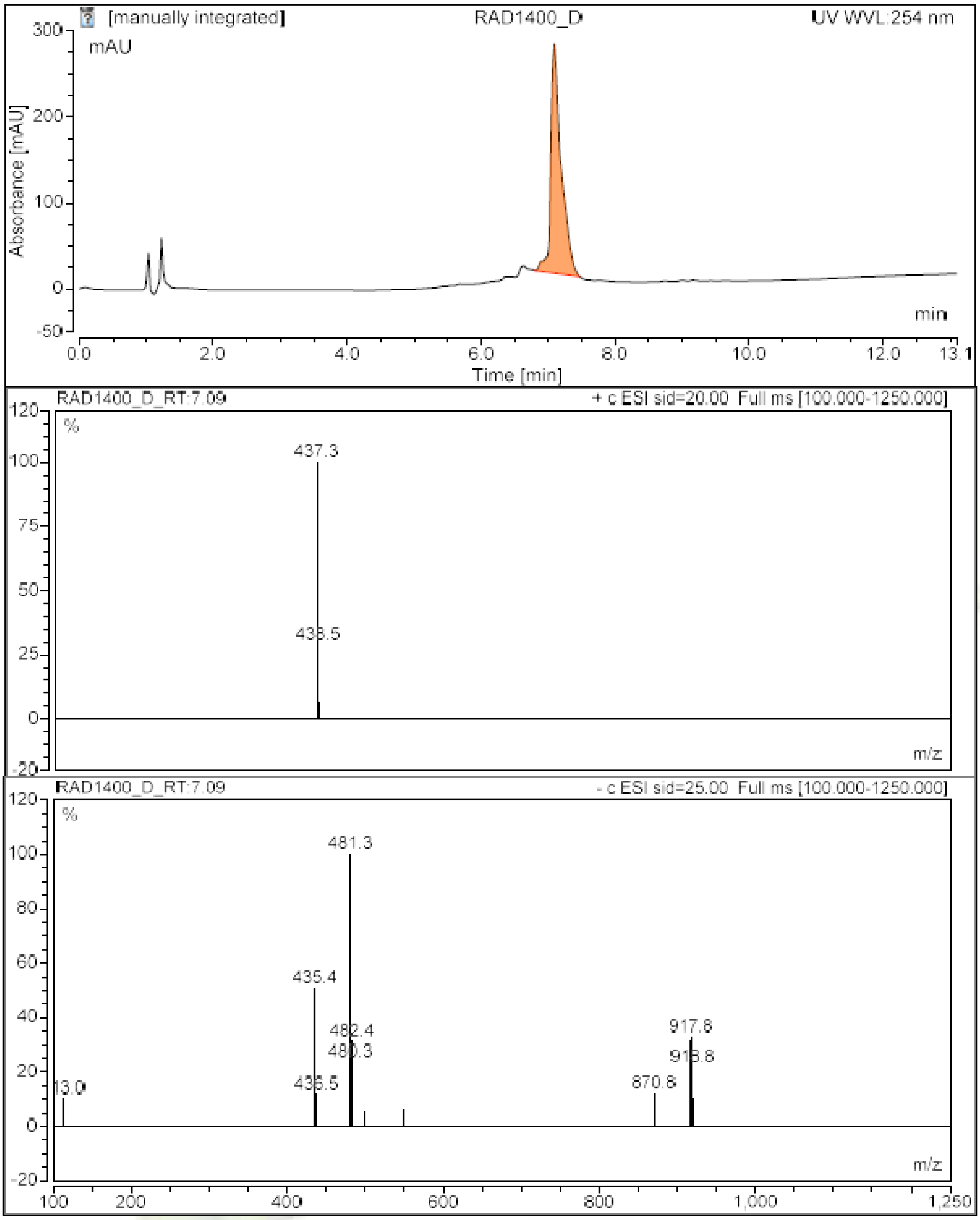

Table S4 DP4+ output for DFT GIAO ^{1}H and ^{13}C NMR shielding tensors for candidate diastereomers (**1a** = isomer 1 and **1b** = isomer 2) compared with experimental NMR data for agelasine Z (**1**) recorded in MeOH-d_4 (C-1 to C-10, C-17 to C-20 and H-1 to H-9, H_3-17 to H_3-20 highlighted red)

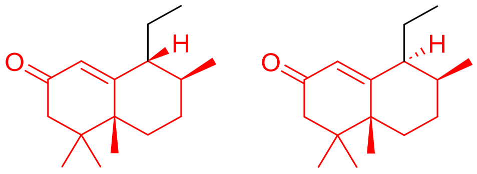

1a = isomer 1 **1b = isomer 2**

mPW1PW91		PCM		6-311+G(d,p)	
Isomer №			1	2	3
DP4+ (%)		H data	95.46%	4.54%	-
		C data	100.00%	0.00%	-
		All data	100.00%	0.00%	-
Type	sp2?	Exp	1	2	3
C	x	122.6	60.2075232	54.661804	
C	x	202.7	-22.140688	-21.76856	
C		47.2	135.56994	134.42681	
C		35.4	143.395133	142.50804	
C		45.7	137.779092	138.25299	
C		31.9	152.137405	152.86964	
C		31	153.993304	159.31934	
C		47.5	143.406312	148.08511	
C		46.1	135.834822	131.61591	
C	x	176.4	-0.9258815	-0.4770867	
C		16.4	164.485189	161.92737	
C		27.7	166.544624	167.78382	
C		28.8	163.240652	162.90691	
C		23.2	163.146937	163.31292	
H	x	5.87	25.7242068	25.79092	
H		2.42	29.5124862	28.909457	
H		2	30.0838333	29.88042	
H		2.11	29.9378313	30.113278	
H		1.43	30.1975539	29.97827	
H		1.64	30.1649879	30.396464	
H		1.64	30.3411725	30.053091	
		1.48	29.4882124	29.947543	
		2.19	29.3966412	29.413887	
		0.98	30.7486017	30.773164	
		1.01	30.8291627	30.79716	
		1.04	30.8128272	30.807024	
		1.2	30.5596602	30.481796	

Default parameters	1	2
sDP4+ (H data)	54.23%	45.77%
sDP4+ (C data)	100.00%	0.00%
sDP4+ (all data)	100.00%	0.00%
uDP4+ (H data)	94.66%	5.34%
uDP4+ (C data)	100.00%	0.00%
uDP4+ (all data)	100.00%	0.00%
DP4+ (H data)	95.46%	4.54%
DP4+ (C data)	100.00%	0.00%
DP4+ (all data)	100.00%	0.00%

Table S5 Agelasine Z (**1**) truncated diastereomer **1a** energies and Boltzmann Factors for geometry optimized (GO) conformers calculated at the B3LYP/6-31* level of theory for GIAO NMR calculations (relative energies >3.0 kcal/mol not shown)

Conformer Number	Energy (kcal/mol)	Relative Energy (kcal/mol)	Boltzmann Factor
2	-439405.1447	0	1
1	-439404.9828	0.161897423	0.760670608
3	-439404.5341	0.610566637	0.356414
6	-439404.1225	1.022212798	0.177779256
5	-439403.3933	1.75137871	0.051856771
7	-439402.4897	2.654992233	0.01126463
4	-439402.4565	2.688250231	0.010649068
9	-439402.1628	2.981924626	0.006483474

Table S6 Agelasine Z (**1**) truncated diastereomer **1b** energies, and Boltzmann Factors for geometry optimized (GO) conformers calculated at the B3LYP/6-31* level of theory for GIAO NMR calculations (relative energies >3.0 kcal/mol not shown)

Conformer Number	Energy (kcal/mol)	Relative Energy (kcal/mol)	Boltzmann Factor
1	-439404.9276	0	1
2	-439403.8828	1.044803136	0.171121221

Table S7 Agelasine Z (**1**) truncated isomer **1a** geometry optimized (GO) conformers (x, y, z coordinates, Gibbs Free energies, % population) calculated at the B3LYP/6-31* level of theory for GIAO NMR calculations

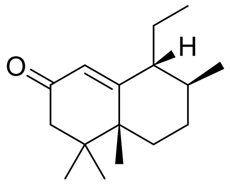

1a

	Conformer 2			Conformer 1			Conformer 3			Conformer 6		
**Gibbs free energy (hartrees)**	-700.236763			-700.236505			-700.23579			-700.235134		
**ΔG (kJ/mol)**				0.677			2.555			4.277		
**Population Proportion**	42.08%			32.02%			15.02%			7.5%		
**Element**	**X**	**Y**	**Z**	**X**	**Y**	**Z**	**X**	**Y**	**Z**	**X**	**Y**	**Z**
C	-1.719655	-1.698538	-2.229917	-2.092124	-2.218471	-3.012909	-1.903864	-2.022374	-2.741168	-1.858337	-1.999154	-2.622291
C	-1.996280	-2.711922	-1.136978	-2.516004	-3.451075	-2.240911	-2.499208	-3.346593	-2.309642	-2.440184	-3.315936	-2.138679
C	-3.431383	-3.281099	-1.193298	-3.251448	-3.113673	-0.925019	-3.351981	-3.208255	-1.029453	-3.466455	-3.158381	-0.991744
C	-4.493857	-2.101425	-1.147310	-4.497346	-2.173835	-1.237573	-4.515701	-2.148311	-1.278866	-4.600846	-2.139265	-1.437313
C	-4.081437	-0.925066	-2.046909	-4.097793	-0.996569	-2.155608	-3.990474	-0.878315	-1.984760	-3.997443	-0.864221	-2.051277
C	-2.833633	-0.791526	-2.544937	-2.992661	-1.057513	-2.930003	-2.779495	-0.849696	-2.581182	-2.754172	-0.833159	-2.573361

C	-4.617666	-1.477922	0.280354	-5.090158	-1.622461	0.089731	-5.143860	-1.732219	0.083269	-5.516617	-1.715804	-0.238712
C	-5.498882	-0.217322	0.328105	-6.160648	-0.548062	-0.095728	-6.117406	-0.557114	0.007309	-5.089993	-0.393974	0.416038
C	-5.161342	0.852891	-0.729520	-5.613367	0.652498	-0.877135	-5.461673	0.690451	-0.603546	-5.208379	0.799004	-0.561561
C	-5.103115	0.202215	-2.154459	-5.075139	0.186081	-2.260689	-4.930641	0.337565	-2.027530	-4.920577	0.348408	-2.033725
C	-6.157187	2.017444	-0.625493	-6.684669	1.746289	-0.996002	-6.458483	1.859808	-0.587231	-6.584846	1.472433	-0.447370
C	-4.856837	1.217718	-3.286492	-4.582281	1.363133	-3.137164	-4.374991	1.553667	-2.802859	-4.483707	1.535680	-2.912815
C	-4.979776	0.623834	-4.696242	-3.478908	2.243051	-2.532072	-4.407402	1.403225	-4.331435	-4.611849	1.295038	-4.422056
C	-3.595102	-4.260610	-0.012052	-2.233701	-2.429459	0.022810	-2.401288	-2.779364	0.117947	-2.697905	-2.675912	0.261071
C	-3.561970	-4.096204	-2.504350	-3.672476	-4.443855	-0.264563	-3.902293	-4.604241	-0.667389	-4.039725	-4.554929	-0.669936
C	-5.881591	-2.652448	-1.575252	-5.609243	-2.972342	-1.977536	-5.625479	-2.766019	-2.174911	-5.511433	-2.786445	-2.519499
O	-0.622169	-1.586265	-2.760880	-1.085298	-2.197109	-3.709834	-0.793230	-1.931005	-3.248061	-0.720446	-1.917050	-3.067418
H	-1.254351	-3.514455	-1.205904	-3.163457	-4.043743	-2.902902	-3.111531	-3.721540	-3.142410	-2.907875	-3.801392	-3.007079
H	-1.822750	-2.206210	-0.175651	-1.628749	-4.064073	-2.049556	-1.687540	-4.067716	-2.167225	-1.611530	-3.964784	-1.834849
H	-2.560666	0.050025	-3.174155	-2.705729	-0.241974	-3.584066	-2.389097	0.051337	-3.040621	-2.337215	0.071685	-3.004814
H	-5.025497	-2.220620	0.976240	-5.494979	-2.457366	0.674012	-5.640610	-2.602706	0.529411	-6.549827	-1.613264	-0.595663
H	-3.615222	-1.221014	0.645390	-4.289091	-1.180754	0.691342	-4.346253	-1.441301	0.773512	-5.546167	-2.516904	0.507261
H	-5.417532	0.229201	1.327839	-6.508944	-0.214989	0.890971	-6.473867	-0.326165	1.019833	-4.057979	-0.475944	0.768959
H	-6.555889	-0.495762	0.215732	-7.044644	-0.950820	-0.609717	-7.011414	-0.817757	-0.576969	-5.704081	-0.205834	1.306065
H	-4.153506	1.240919	-0.516968	-4.768356	1.060842	-0.302546	-4.598461	0.964373	0.023561	-4.442794	1.540183	-0.292941
H	-6.095067	-0.231558	-2.340950	-5.955279	-0.222213	-2.787771	-5.823318	0.007683	-2.586253	-5.885958	-0.002485	-2.432592
H	-5.861089	2.878016	-1.232384	-6.291402	2.680063	-1.408948	-5.993593	2.822173	-0.819394	-6.711511	2.283592	-1.172816
H	-6.230383	2.360879	0.413334	-7.102951	1.975172	-0.008696	-6.910680	1.951836	0.407328	-6.725890	1.898433	0.552739
H	-7.161993	1.709156	-0.942917	-7.513275	1.418250	-1.637541	-7.272604	1.696311	-1.305698	-7.392456	0.748399	-0.616955
H	-5.598983	2.017519	-3.191150	-4.254165	0.973599	-4.107334	-4.980105	2.429023	-2.551592	-5.103503	2.400511	-2.646312
H	-3.879655	1.702104	-3.160746	-5.449218	1.993779	-3.364466	-3.360146	1.790289	-2.458990	-3.455627	1.824692	-2.659002
H	-4.838087	1.399029	-5.457639	-3.163290	3.006417	-3.252141	-4.001588	2.297913	-4.817184	-4.294124	2.178767	-4.986727
H	-4.242916	-0.163812	-4.879507	-3.825391	2.766087	-1.633785	-3.832421	0.542469	-4.684453	-4.003420	0.448833	-4.755952
H	-5.975418	0.188682	-4.849038	-2.593913	1.662329	-2.253968	-5.439081	1.277002	-4.682844	-5.653053	1.084110	-4.696195
H	-3.370702	-3.790346	0.950634	-1.984792	-1.412081	-0.294762	-2.075412	-1.738360	0.027422	-2.249309	-1.687783	0.121589
H	-4.604586	-4.683077	0.044198	-2.599467	-2.383818	1.053126	-2.862474	-2.906893	1.102017	-3.339298	-2.639053	1.147278
H	-2.899916	-5.099414	-0.134553	-1.302800	-3.007803	0.038831	-1.503701	-3.408118	0.100453	-1.883845	-3.376698	0.480021
H	-2.725603	-4.800043	-2.584636	-2.776586	-5.019084	-0.002559	-3.068931	-5.267592	-0.407620	-3.237695	-5.203787	-0.299183
H	-4.484570	-4.683700	-2.530518	-4.238032	-4.286742	0.660454	-4.572931	-4.571815	0.198728	-4.811425	-4.517322	0.107591
H	-3.542545	-3.457491	-3.393374	-4.274811	-5.069314	-0.930834	-4.440740	-5.070514	-1.498231	-4.470107	-5.041942	-1.550217
H	-5.931338	-2.838771	-2.652032	-5.227817	-3.465439	-2.876593	-5.219022	-3.160151	-3.111752	-4.938994	-3.193677	-3.358214
H	-6.091370	-3.595766	-1.061748	-6.042288	-3.736929	-1.324849	-6.138985	-3.581239	-1.656131	-6.113919	-3.593340	-2.090618
H	-6.695867	-1.970309	-1.324417	-6.426299	-2.319359	-2.294623	-6.384805	-2.026993	-2.441717	-6.207473	-2.047514	-2.929113

	**Conformer 5**			**Conformer 7**			**Conformer 4**			**Conformer 9**		
**Gibbs free energy (hartrees)**	-700.233972			-700.232532			-700.232479			-700.232011		
**ΔG (kJ/mol)**	7.328			11.108			11.248			12.476		
**Population Proportion**	2.19%			0.48%			0.45%			0.27%		
**Element**	**X**	**Y**	**Z**	**X**	**Y**	**Z**	**X**	**Y**	**Z**	**X**	**Y**	**Z**
C	-1.702807	-1.905215	-2.298999	-1.885427	-2.196334	-2.761965	-1.851857	-1.882480	-2.539749	-1.917796	-2.243658	-2.763300
C	-2.000966	-2.858307	-1.158305	-2.540886	-3.481772	-2.302615	-1.954018	-2.666300	-1.246822	-2.510346	-3.492068	-2.136693
C	-3.462216	-3.359825	-1.156763	-3.373687	-3.271707	-1.019412	-3.359403	-3.274971	-1.049412	-3.450700	-3.197323	-0.945423
C	-4.467087	-2.129936	-1.140701	-4.494285	-2.169605	-1.289488	-4.461219	-2.127835	-1.070543	-4.576028	-2.170922	-1.399513
C	-4.022566	-1.017446	-2.103525	-3.925322	-0.945843	-2.047309	-4.176344	-1.072709	-2.155377	-3.965734	-0.966097	-2.139915
C	-2.780367	-0.960729	-2.629675	-2.710991	-0.983523	-2.636626	-3.018546	-1.040990	-2.849145	-2.759196	-1.036793	-2.740867
C	-4.526216	-1.436816	0.258698	-5.083783	-1.685700	0.068782	-4.508250	-1.334077	0.271754	-5.395334	-1.641909	-0.174944
C	-5.337048	-0.128834	0.263601	-5.983829	-0.453612	-0.019472	-5.496775	-0.158160	0.243318	-4.931594	-0.260511	0.301929
C	-4.965309	0.866847	-0.853117	-5.263454	0.723907	-0.691230	-5.337231	0.792407	-0.958828	-5.197675	0.819656	-0.766960
C	-4.995070	0.148856	-2.247819	-4.835031	0.294570	-2.129177	-5.240475	0.010297	-2.310484	-4.836157	0.291706	-2.202492
C	-5.855587	2.112956	-0.759261	-6.132330	1.986038	-0.618514	-6.481893	1.816927	-0.956476	-6.654338	1.308269	-0.675221
C	-4.717485	1.079227	-3.453551	-4.292118	1.453648	-3.002465	-5.076146	0.940598	-3.540958	-4.251477	1.386890	-3.119623
C	-5.977330	1.694449	-4.079914	-5.366955	2.213098	-3.796367	-4.053674	2.085868	-3.446978	-5.142279	2.610619	-3.363944
C	-3.643274	-4.276869	0.071149	-2.392322	-2.853313	0.106231	-3.357329	-4.056486	0.281114	-2.585422	-2.640919	0.210401
C	-3.663137	-4.224270	-2.426537	-3.978403	-4.632274	-0.611814	-3.586411	-4.298276	-2.191390	-4.050052	-4.538445	-0.471449
C	-5.889950	-2.632803	-1.508905	-5.643240	-2.770464	-2.146622	-5.851784	-2.777223	-1.316690	-5.572130	-2.855978	-2.377618
O	-0.612813	-1.866480	-2.855298	-0.769286	-2.164994	-3.263751	-0.845092	-1.879925	-3.236069	-0.814949	-2.245139	-3.295921
H	-1.299781	-3.697957	-1.209636	-3.178727	-3.842573	-3.122272	-1.184425	-3.445163	-1.242836	-3.052178	-4.027508	-2.929317
H	-1.779613	-2.322224	-0.223610	-1.764476	-4.238727	-2.150384	-1.715399	-1.977570	-0.423031	-1.686799	-4.147452	-1.832815
H	-2.481609	-0.155763	-3.294409	-2.275225	-0.114600	-3.116883	-2.852080	-0.325047	-3.646247	-2.329297	-0.190548	-3.265858
H	-4.957678	-2.124258	0.995934	-5.626454	-2.515689	0.538053	-4.783653	-2.005066	1.093330	-6.456440	-1.583030	-0.448220
H	-3.503988	-1.217777	0.591852	-4.263227	-1.428988	0.744962	-3.503873	-0.953210	0.498949	-5.348392	-2.364133	0.646074
H	-5.206382	0.360703	1.237714	-6.293181	-0.168650	0.994962	-5.384476	0.421786	1.169001	-3.862533	-0.291044	0.537242
H	-6.410326	-0.352223	0.188394	-6.910094	-0.671918	-0.569911	-6.526698	-0.538849	0.260758	-5.446522	0.008949	1.233153
H	-3.923348	1.185215	-0.695820	-4.339167	0.925654	-0.126225	-4.386428	1.330165	-0.833558	-4.539044	1.673782	-0.555948
H	-6.008694	-0.251117	-2.380402	-5.757431	-0.051248	-2.623545	-6.208175	-0.492151	-2.447596	-5.785951	-0.032104	-2.658915
H	-5.543794	2.897835	-1.454628	-5.616642	2.872668	-0.998468	-6.359389	2.589133	-1.721403	-6.897907	2.046710	-1.442945

H	-5.805866	2.533773	0.252410	-6.402199	2.187395	0.425236	-6.541910	2.321662	0.015219	-6.843099	1.767806	0.302424
H	-6.906166	1.872279	-0.964981	-7.066245	1.865844	-1.181497	-7.446611	1.323005	-1.131652	-7.359384	0.475243	-0.789051
H	-4.014207	1.872450	-3.165613	-3.712925	2.150677	-2.381507	-4.844411	0.316131	-4.412749	-3.293212	1.726614	-2.703805
H	-4.215452	0.502484	-4.238034	-3.592480	1.059797	-3.743167	-6.057638	1.381689	-3.754152	-4.020345	0.934710	-4.091719
H	-5.711947	2.320121	-4.939858	-4.903200	2.990310	-4.414816	-3.991860	2.607566	-4.408871	-4.673978	3.271363	-4.102265
H	-6.653300	0.909300	-4.440653	-5.898904	1.531440	-4.471774	-4.340030	2.828236	-2.694699	-6.126003	2.324222	-3.755643
H	-6.540290	2.317030	-3.379000	-6.113286	2.697681	-3.162487	-3.047575	1.739504	-3.194792	-5.299320	3.200500	-2.455177
H	-3.376065	-3.774886	1.006520	-2.027255	-1.828260	-0.012214	-3.033998	-3.439429	1.125381	-2.112248	-1.687942	-0.044408
H	-4.669380	-4.648992	0.167423	-2.845073	-2.940510	1.098573	-4.342879	-4.471206	0.521815	-3.165845	-2.499160	1.127519
H	-2.989884	-5.151698	-0.027098	-1.520030	-3.516819	0.092454	-2.660201	-4.899360	0.207893	-1.784556	-3.352990	0.441147
H	-2.863728	-4.971129	-2.493456	-3.171037	-5.320366	-0.334944	-2.724713	-4.972673	-2.254639	-3.247986	-5.186763	-0.099819
H	-4.613282	-4.766362	-2.406339	-4.642344	-4.545669	0.255780	-4.471238	-4.917333	-2.016336	-4.766699	-4.408105	0.347451
H	-3.634200	-3.626400	-3.343304	-4.539657	-5.102140	-1.425312	-3.698977	-3.815694	-3.167574	-4.554301	-5.077526	-1.279309
H	-5.978331	-2.861151	-2.575113	-5.271568	-3.198811	-3.083049	-5.954881	-3.136783	-2.344327	-5.068442	-3.323914	-3.228851
H	-6.128600	-3.543194	-0.950566	-6.170157	-3.556594	-1.597692	-5.996040	-3.630607	-0.646166	-6.160483	-3.621852	-1.862642
H	-6.664866	-1.903468	-1.265822	-6.385729	-2.014245	-2.412486	-6.675317	-2.086511	-1.130411	-6.279700	-2.123812	-2.779810

Table S8 Agelasine Z (**1**) truncated isomer **1b** geometry optimized (GO) conformers (x, y, z coordinates, Gibbs Free energies, % population) calculated at the B3LYP/6-31* level of theory for GIAO NMR calculations

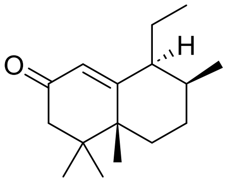

1b

	Conformer 1			Conformer 2		
**Gibbs free energy (hartrees)**	-700.236417			-700.234752		
**ΔG (kJ/mol)**				4.371		
**Population Proportion**	85.36%			14.64%		
**Element**	**X**	**Y**	**Z**	**X**	**Y**	**Z**
C	-9.017116	11.498260	-10.774623	-9.107161	11.556984	-10.718614
C	-9.646921	11.367355	-9.400211	-9.779405	11.373851	-9.369722
C	-10.000385	9.908728	-9.028580	-10.098174	9.897396	-9.037723
C	-8.693309	9.003378	-9.139977	-8.762522	9.035218	-9.129417
C	-7.995069	9.235675	-10.496947	-8.026171	9.324569	-10.451718
C	-8.184956	10.367127	-11.210443	-8.221541	10.465420	-11.147649
C	-9.065173	7.498377	-8.976801	-9.086966	7.514728	-9.026847
C	-7.974940	6.512514	-9.410083	-7.926953	6.587762	-9.405368
C	-7.588249	6.750864	-10.875494	-7.460550	6.867412	-10.839851
C	-7.030474	8.188560	-11.062985	-6.980753	8.337680	-10.980149
C	-6.669364	5.651626	-11.424471	-6.444837	5.829624	-11.332569
C	-5.563422	8.340264	-10.561483	-5.566181	8.616939	-10.368619
C	-4.948517	9.729602	-10.756681	-4.431891	8.579748	-11.401678
C	-11.120081	9.432948	-9.989746	-11.173594	9.406726	-10.040321
C	-10.593946	9.911727	-7.603888	-10.730588	9.852817	-7.630600
C	-7.683830	9.368348	-8.014124	-7.799566	9.393419	-7.961142
O	-9.159324	12.498752	-11.466231	-9.259096	12.568943	-11.391381
H	-6.983738	8.378042	-12.144736	-6.891755	8.546343	-12.054575
H	-8.937410	11.788188	-8.673673	-9.108674	11.801445	-8.610884
H	-10.535539	12.006920	-9.365941	-10.689047	11.983880	-9.352675
H	-7.693648	10.505019	-12.171487	-7.692963	10.642873	-12.082639
H	-9.341861	7.314948	-7.931710	-9.426400	7.293328	-8.007936
H	-9.953009	7.273668	-9.575885	-9.921720	7.271794	-9.691891
H	-8.353826	5.488972	-9.288851	-8.262571	5.545425	-9.324014
H	-7.089031	6.584278	-8.765301	-7.088063	6.689535	-8.703867
H	-8.521406	6.710168	-11.459168	-8.350402	6.776637	-11.482625
H	-6.352423	5.867009	-12.452426	-6.145670	6.018142	-12.370215
H	-7.195396	4.689867	-11.434166	-6.881416	4.824702	-11.290679
H	-5.767865	5.523932	-10.814915	-5.537947	5.817267	-10.717081
H	-5.478393	8.048629	-9.508510	-5.566363	9.608315	-9.903243
H	-4.958525	7.616694	-11.120098	-5.350813	7.903729	-9.562808
H	-3.894070	9.723708	-10.457809	-3.466910	8.789903	-10.925821

H	-4.993278	10.040846	-11.807408	-4.354243	7.606505	-11.896209
H	-5.460737	10.495489	-10.166742	-4.589442	9.336301	-12.179767
H	-10.752748	9.247965	-11.003664	-10.772502	9.263081	-11.048220
H	-11.606724	8.519982	-9.633029	-11.633627	8.466660	-9.720414
H	-11.897054	10.203246	-10.055159	-11.977216	10.149065	-10.106966
H	-11.535416	10.473703	-7.603385	-11.687591	10.387704	-7.644322
H	-10.820347	8.900116	-7.249238	-10.937028	8.827730	-7.303278
H	-9.933046	10.389975	-6.874305	-10.103318	10.333496	-6.873400
H	-7.378954	10.418160	-8.056371	-7.541916	10.456696	-7.950955
H	-8.117307	9.176640	-7.027839	-8.250849	9.140084	-6.996895
H	-6.775608	8.766979	-8.086165	-6.863008	8.836920	-8.034169

Table S9 Agelasine Z (**1a** - 5*R*, 8*S* and 9*R*) energies and Boltzmann Factors for geometry optimized (GO) truncated conformers calculated at the B3LYP/6-311G(d,p) level of theory for TDDFT ECD calculations (relative energies >3.0 kcal/mol not shown)

Conformer Number	Energy (kcal/mol)	Relative Energy (kcal/mol)	Boltzmann Factor
2	-439527.2762	0	1
1	-439527.1106	0.165662479	0.755846779
3	-439526.6864	0.589858828	0.369105515
6	-439526.2195	1.056725814	0.167708389
5	-439525.5104	1.765811426	0.050607452
7	-439524.753	2.523215261	0.014073993
4	-439524.5892	2.686995212	0.010671674

Table S10 Agelasine Z (**1a** - 5*R*, 8*S* and 9*R*) geometry optimized (GO) truncated conformers (x, y, z coordinates, Gibbs Free energies, % population) calculated at the B3LYP/6-311G(d,p) level of theory for TDDFT ECD calculations

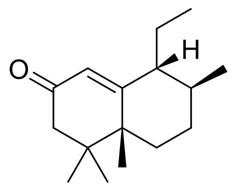

1a

	Conformer 2			Conformer 1			Conformer 3			Conformer 6		
**Gibbs free energy (hartrees)**	-700.431392			-700.431128			-700.430452			-700.429708		
**ΔG (kJ/mol)**				0.693			2.468			4.421		
**Population Proportion**	42.21%			31.91%			15.6%			7.09%		
**Element**	**X**	**Y**	**Z**	**X**	**Y**	**Z**	**X**	**Y**	**Z**	**X**	**Y**	**Z**
C	-1.727444	-1.692475	-2.221742	-2.109872	-2.219616	-3.018420	-1.925037	-2.032005	-2.757327	-1.869319	-1.997218	-2.621766
C	-2.000486	-2.711376	-1.141751	-2.520815	-3.445500	-2.240635	-2.505913	-3.349230	-2.307057	-2.441049	-3.309298	-2.133502
C	-3.434084	-3.280664	-1.195244	-3.251846	-3.109880	-0.923619	-3.351154	-3.205984	-1.024158	-3.467414	-3.155643	-0.988008
C	-4.493802	-2.100417	-1.146041	-4.497315	-2.171281	-1.234691	-4.514531	-2.147861	-1.273776	-4.601303	-2.138949	-1.435187
C	-4.080688	-0.926233	-2.042090	-4.099270	-0.998057	-2.152711	-3.991075	-0.883457	-1.983364	-3.999005	-0.867473	-2.048837
C	-2.834700	-0.793202	-2.541313	-2.997848	-1.060221	-2.930094	-2.787458	-0.860929	-2.591980	-2.757748	-0.836137	-2.572510
C	-4.610551	-1.475089	0.281052	-5.087958	-1.620684	0.091969	-5.140773	-1.730726	0.086812	-5.516955	-1.715835	-0.238848
C	-5.494286	-0.219014	0.328033	-6.158225	-0.549121	-0.094867	-6.117176	-0.561332	0.004943	-5.089627	-0.395755	0.415182
C	-5.162405	0.849265	-0.730554	-5.614218	0.650565	-0.876658	-5.467340	0.685854	-0.608147	-5.204566	0.795160	-0.562565

C	-5.098810	0.198378	-2.155837	-5.069461	0.185636	-2.257266	-4.922505	0.334830	-2.026381	-4.916612	0.344370	-2.034164
C	-6.166885	2.005431	-0.633354	-6.690689	1.736607	-1.005297	-6.474983	1.844741	-0.609359	-6.578917	1.471222	-0.453301
C	-4.845110	1.213953	-3.283873	-4.572881	1.360608	-3.131966	-4.353396	1.551295	-2.788656	-4.477996	1.529292	-2.912737
C	-4.980246	0.629390	-4.695699	-3.474476	2.243573	-2.523873	-4.404166	1.426827	-4.318350	-4.621939	1.296287	-4.421173
C	-3.593401	-4.258498	-0.013673	-2.232573	-2.428977	0.023121	-2.396247	-2.774960	0.117393	-2.702589	-2.675968	0.266928
C	-3.567377	-4.097563	-2.503738	-3.671820	-4.440236	-0.265778	-3.899646	-4.599981	-0.657329	-4.038268	-4.552836	-0.671138
C	-5.882851	-2.646268	-1.570485	-5.608336	-2.967441	-1.977317	-5.623580	-2.763254	-2.171284	-5.506997	-2.784527	-2.521868
O	-0.624769	-1.574973	-2.746565	-1.108397	-2.204686	-3.727205	-0.820690	-1.947129	-3.284166	-0.730991	-1.916765	-3.072476
H	-1.261719	-3.513121	-1.217729	-3.167810	-4.038537	-2.898116	-3.120138	-3.731349	-3.131314	-2.906501	-3.793855	-3.000092
H	-1.825353	-2.209909	-0.181800	-1.631924	-4.052347	-2.050166	-1.691569	-4.063239	-2.161649	-1.612161	-3.952797	-1.827195
H	-2.565673	0.047320	-3.170146	-2.714705	-0.244320	-3.582181	-2.402365	0.036683	-3.057897	-2.346079	0.068360	-3.004883
H	-5.015120	-2.217976	0.973610	-5.490898	-2.455792	0.671542	-5.633606	-2.600740	0.530538	-6.545914	-1.613159	-0.598238
H	-3.609784	-1.217382	0.641845	-4.288474	-1.180408	0.691739	-4.345461	-1.437274	0.774427	-5.546405	-2.516253	0.503256
H	-5.408549	0.229964	1.323080	-6.502326	-0.214263	0.889455	-6.471973	-0.327154	1.014110	-4.060942	-0.476923	0.770412
H	-6.548002	-0.499781	0.219226	-7.039993	-0.952129	-0.606549	-7.007224	-0.826609	-0.578050	-5.705233	-0.205421	1.299912
H	-4.160166	1.245125	-0.518309	-4.776006	1.064712	-0.301384	-4.613669	0.971791	0.022185	-4.438531	1.532425	-0.295857
H	-6.086227	-0.236555	-2.345053	-5.942793	-0.225159	-2.787485	-5.806016	0.006011	-2.594522	-5.878611	-0.008087	-2.431247
H	-5.877248	2.864345	-1.240938	-6.303399	2.667305	-1.423905	-6.020086	2.807096	-0.848588	-6.698800	2.282847	-1.175409
H	-6.241109	2.348833	0.402642	-7.107926	1.968479	-0.021157	-6.929603	1.940213	0.380868	-6.719752	1.893571	0.545599
H	-7.165547	1.686054	-0.950051	-7.513668	1.397061	-1.643704	-7.281310	1.663011	-1.328294	-7.384074	0.749823	-0.627877
H	-5.577277	2.017938	-3.182691	-4.241123	0.972439	-4.098642	-4.937776	2.430649	-2.517051	-5.088717	2.394364	-2.638957
H	-3.865257	1.687504	-3.159564	-5.437551	1.987840	-3.363311	-3.333224	1.763941	-2.453125	-3.448329	1.810422	-2.667513
H	-4.840495	1.407859	-5.450677	-3.162948	3.006951	-3.242103	-3.989011	2.320812	-4.792031	-4.310060	2.181439	-4.982290
H	-4.249474	-0.158186	-4.890828	-3.823584	2.762665	-1.627743	-3.847896	0.564746	-4.691284	-4.018618	0.453998	-4.767143
H	-5.976719	0.201023	-4.842603	-2.588217	1.668841	-2.246079	-5.439166	1.323403	-4.658692	-5.663917	1.087974	-4.683187
H	-3.372779	-3.785175	0.945069	-1.971184	-1.417806	-0.296307	-2.061377	-1.739817	0.020726	-2.253248	-1.689736	0.134751
H	-4.598688	-4.683411	0.038957	-2.604636	-2.373672	1.047358	-2.859169	-2.889614	1.098705	-3.347419	-2.641418	1.146983
H	-2.894422	-5.090858	-0.136696	-1.311887	-3.018917	0.047044	-1.508701	-3.414014	0.103905	-1.894140	-3.379831	0.484587
H	-2.735903	-4.804293	-2.577384	-2.776352	-5.016869	-0.016007	-3.064773	-5.262219	-0.410370	-3.234905	-5.198567	-0.305314
H	-4.490802	-4.678251	-2.525969	-4.226074	-4.281862	0.662275	-4.556293	-4.564152	0.215417	-4.805530	-4.515296	0.106453
H	-3.546474	-3.465069	-3.394383	-4.281602	-5.056898	-0.928705	-4.448632	-5.060345	-1.480714	-4.468948	-5.033765	-1.551040
H	-5.933659	-2.838121	-2.643576	-5.227996	-3.456213	-2.876069	-5.218676	-3.153175	-3.107460	-4.932514	-3.187219	-3.358061
H	-6.093520	-3.582747	-1.050890	-6.036159	-3.731273	-1.325365	-6.131868	-3.577762	-1.652523	-6.104172	-3.591717	-2.093817
H	-6.689518	-1.958835	-1.322817	-6.423752	-2.314447	-2.288256	-6.381529	-2.024832	-2.431457	-6.203177	-2.047287	-2.926247

	**Conformer 5**			**Conformer 7**			**Conformer 4**		
**Gibbs free energy (hartrees)**	-700.428578			-700.427371			-700.42711		
**ΔG (kJ/mol)**	7.388			10.557			11.242		
**Population Proportion**	2.14%			0.6%			0.45%		
**Element**	**X**	**Y**	**Z**	**X**	**Y**	**Z**	**X**	**Y**	**Z**
C	-1.710896	-1.896880	-2.291043	-1.917948	-2.208947	-2.793521	-1.859446	-1.872071	-2.530431
C	-2.006814	-2.857308	-1.164428	-2.556085	-3.485515	-2.307103	-1.959565	-2.664502	-1.250054
C	-3.466813	-3.357954	-1.160918	-3.374007	-3.266175	-1.017806	-3.363115	-3.274127	-1.052471
C	-4.468051	-2.126889	-1.140022	-4.493763	-2.164696	-1.284358	-4.462779	-2.126911	-1.070138
C	-4.022494	-1.015686	-2.098294	-3.927172	-0.945317	-2.045098	-4.177035	-1.072521	-2.150188
C	-2.782071	-0.960446	-2.626327	-2.726659	-0.995626	-2.658646	-3.019300	-1.037312	-2.841723
C	-4.519481	-1.434362	0.259678	-5.080540	-1.685431	0.074331	-4.503470	-1.331940	0.271278
C	-5.330941	-0.129432	0.266163	-5.985428	-0.460050	-0.014907	-5.492592	-0.159164	0.243608
C	-4.962936	0.864978	-0.850019	-5.270818	0.719682	-0.683452	-5.334923	0.790103	-0.957340
C	-4.991200	0.147763	-2.245970	-4.826197	0.299304	-2.118399	-5.237775	0.007345	-2.308817
C	-5.858619	2.106012	-0.761304	-6.153199	1.972085	-0.625861	-6.481430	1.810618	-0.960151
C	-4.711993	1.080375	-3.447620	-4.274727	1.471345	-2.966664	-5.072442	0.934104	-3.539734
C	-5.973347	1.679731	-4.084380	-5.336613	2.199714	-3.804384	-4.053139	2.081214	-3.445761
C	-3.644420	-4.275136	0.065814	-2.382428	-2.844169	0.095935	-3.357317	-4.055749	0.276472
C	-3.671154	-4.222272	-2.429028	-3.976558	-4.623005	-0.599729	-3.591164	-4.296731	-2.193433
C	-5.892230	-2.623227	-1.505327	-5.642884	-2.760059	-2.144318	-5.854325	-2.771364	-1.314472
O	-0.614876	-1.852021	-2.840551	-0.812506	-2.186974	-3.324015	-0.847744	-1.864224	-3.223817
H	-1.309477	-3.696654	-1.223854	-3.200438	-3.856167	-3.113284	-1.193376	-3.443450	-1.252386
H	-1.783584	-2.326910	-0.230321	-1.776010	-4.235174	-2.153811	-1.720988	-1.980748	-0.426046
H	-2.487395	-0.156956	-3.291374	-2.297601	-0.133971	-3.152947	-2.855173	-0.318586	-3.634278
H	-4.949083	-2.123200	0.991909	-5.617842	-2.517655	0.538353	-4.776961	-2.003786	1.088569
H	-3.498818	-1.216826	0.589492	-4.262025	-1.427543	0.748335	-3.500966	-0.952256	0.495221
H	-5.194850	0.360498	1.236039	-6.293508	-0.176018	0.997009	-5.376582	0.421144	1.165127
H	-6.401361	-0.353025	0.194076	-6.907592	-0.681918	-0.565163	-6.519411	-0.539744	0.262087
H	-3.924847	1.188343	-0.693857	-4.355681	0.932659	-0.112588	-4.387914	1.329518	-0.832489
H	-6.000662	-0.253559	-2.378686	-5.739756	-0.042832	-2.625137	-6.201036	-0.496813	-2.444283
H	-5.552527	2.887742	-1.458662	-5.646833	2.860191	-1.007487	-6.361814	2.576883	-1.727816
H	-5.805861	2.527946	0.246849	-6.429601	2.174713	0.413085	-6.537813	2.318618	0.007069
H	-6.905337	1.857225	-0.962769	-7.079178	1.834831	-1.193142	-7.441549	1.312027	-1.130295
H	-4.023383	1.879857	-3.152008	-3.749524	2.181325	-2.318073	-4.838897	0.310342	-4.408510

H	-4.197175	0.512477	-4.226585	-3.530193	1.106636	-3.673055	-6.052323	1.370582	-3.754565
H	-5.707676	2.308298	-4.939079	-4.874448	2.999529	-4.390122	-3.991666	2.599438	-4.406812
H	-6.633441	0.887089	-4.450241	-5.808417	1.506837	-4.508302	-4.342181	2.822164	-2.697102
H	-6.547841	2.292732	-3.388541	-6.127240	2.646174	-3.200988	-3.048586	1.738678	-3.191855
H	-3.380084	-3.772021	0.997972	-2.008863	-1.825935	-0.033101	-3.038852	-3.437354	1.117823
H	-4.667168	-4.648442	0.157886	-2.831579	-2.915680	1.087656	-4.339340	-4.473394	0.512313
H	-2.988455	-5.144694	-0.034822	-1.521018	-3.518091	0.084010	-2.656727	-4.892309	0.200619
H	-2.876683	-4.971439	-2.490518	-3.167125	-5.311438	-0.340083	-2.732758	-4.972124	-2.250869
H	-4.621725	-4.757260	-2.405217	-4.620555	-4.531418	0.278345	-4.476019	-4.909813	-2.016669
H	-3.641826	-3.629663	-3.346485	-4.553873	-5.086365	-1.401756	-3.703405	-3.818128	-3.169104
H	-5.982660	-2.853337	-2.568303	-5.273618	-3.180806	-3.081993	-5.960175	-3.128445	-2.339974
H	-6.130651	-3.528220	-0.943666	-6.164476	-3.547300	-1.597793	-5.996070	-3.622002	-0.644631
H	-6.659102	-1.889853	-1.262674	-6.383534	-2.002972	-2.400399	-6.671182	-2.078319	-1.125400
